# Identification and *in silico* analysis of functional SNPs of human TAGAP protein: A comprehensive study

**DOI:** 10.1371/journal.pone.0188143

**Published:** 2018-01-12

**Authors:** Maria Arshad, Attya Bhatti, Peter John

**Affiliations:** Atta-Ur-Rahman School of Applied Biosciences, National University of Sciences & Technology, Islamabad, Pakistan; University of Michigan, UNITED STATES

## Abstract

Genetic polymorphisms in *TAGAP* gene have been associated with many diseases including rheumatoid arthritis, multiple sclerosis and other autoimmune disorders. Identifying functional SNPs in such disease associated genes is an uphill task hence before planning larger population study, it is better to scrutinize putative functional SNPs. In this study we used various computational approaches to identify nsSNPs which are deleterious to the structure and/or function of *TAGAP* protein that might be causing these diseases. Computational analysis was performed by five different *in silico* tools including SIFT, PROVEAN, PolyPhen-2, PhD-SNP and SNPs&GO. The study concludes that mutations of Glycine → Glutamic Acid at position 120, Glycine → Tryptophan at position 141 and Valine → Methionine at position 151 are major mutations in native *TAGAP* protein which might contribute to its malfunction and ultimately causing disease. The study also proposed 3D structures of native *TAGAP* protein and its three mutants. Future studies should consider these nsSNPs as main target mutations in various diseases involving *TAGAP* malfunction. This is the first comprehensive study, where *TAGAP* gene variants were analyzed using *in silico* tools hence will be of great help while considering large scale studies and also in developing precision medicines for cure of diseases related to these polymorphisms. Furthermore, animal models of various autoimmune diseases and having these mutations might be of help in exploring their precise roles.

## Introduction

Genetic polymorphisms in human genome are mostly (90%) single nucleotide polymorphisms (SNPs) which are single base pair changes in alleles and are considered to be the most common kind of variations in DNA sequence. The SNPs in coding region of human genome are of much importance and around 500,000 SNPs fall in this region [1]. Among these the non-synonymous SNPs (nsSNPs), also named as missense SNPs, are highly significant as they are responsible for amino acid residue substitutions resulting in functional diversity of proteins in humans [2]. Functional variations can have deleterious or neutral effects on protein structure or function [3]. Damaging effects might include destabilization of protein structure, altering gene regulation [4], affecting protein charge, geometry, hydrophobicity [5], stability, dynamics, translation and inter/intra protein interactions [2,6,7], hence structural integrity of cells comes under risk [8]. Thus it can be avowed that nsSNPs might get linked with many human diseases because of these missense SNPs.

Numerous studies in the past have shown that nsSNPs are responsible for about 50% of mutations which are involved in various genetic disorders [9,10] including many inflammatory and autoimmune disorders [11–15]. A study analyzed genetic variations in ABCA1 gene and predicted their deleterious effects causing familial hypoalphalipoproteinemia and tangier disease [16]. Similar study identified missense SNPs in STEAP2 which cause its upregulation leading to prostate cancer [17]. The nsSNPs in NKX2-5 gene were found associated with congenital heart defects because of their damaging effects on structural features of the protein [18]. A recent study proposed that nsSNPs in MITF gene might cause malignant melanoma [19]. Another latest study on nsSNPs and their effects on patients with non-small cell lung cancer treated with immunotherapy suggested that the combination of deleterious SNPs and known pathogenic lesions might help in getting advantage from immunotherapy [20].This study is aimed to investigate nsSNPs of T-cell Activation Rho GTPase Activating Protein (*TAGAP*) and their effects on its structure and function. This protein, located on chromosome 6q25, acts as a molecular switch and is considered important in modulating cytoskeletal changes [21], in activation of T cells [22] and is therefore of particular interest in the context of T cell-driven autoimmune disease processes. For this purpose, we made use of various bioinformatics tools to come up with the most deleterious and damaging nsSNPs of *TAGAP* protein. 3D models of *TAGAP* protein and its mutant forms are also proposed in this study. This is the first ever study which covers an extensive *in silico* analysis of nsSNPs of *TAGAP* protein hence this work might be useful in future in developing precision medicines for the treatment of diseases caused by these genomic variations.

## Materials and methods

### Retrieving nsSNPs

Information of missense SNPs (SNP ID, protein accession number, position, residue change and global minor allele frequency (MAF) was retrieved from NCBI dbSNP database (https://www.ncbi.nlm.nih.gov/projects/SNP/) [23]. All 275 nsSNPs were filtered for investigation.

### Identifying the most damaging nsSNPs

We utilized five different bioinformatics tools to predict functional effects of nsSNPs recruited from dbSNP database. These algorithmic programs included: SIFT-Sorting Intolerant From Tolerant [http://sift.jcvi.org/www/SIFT_seq_submit2.html] [24,25], PROVEAN-Protein Variation Effect Analyzer [http://provean.jcvi.org/index.php] [26], PolyPhen-2-Polymorphism Phenotyping v2 [http://genetics.bwh.harvard.edu/pph2/] [27], PhD-SNP -Predictor of human Deleterious Single Nucleotide Polymorphisms [http://snps.biofold.org/phd-snp/phd-snp.html] [28] and SNPs&GO [http://snps.biofold.org/snps-and-go/snps-and-go.html] [29]. The SNPs predicted deleterious by at least four *in silico* tools were considered high risk nsSNPs and investigated further.

### Identifying structural and functional properties

For sorting disease associated or neutral amino acid substitutions in humans, MutPred v1.2 was consulted which is a web based application tool that effectively screens amino acid substitutions [30]. It also helps predicting molecular cause of the disease. MutPred is based upon gain/loss of 14 different functional and structural properties like loss of a phosphorylation site or gain of helical propensity. Protein sequence (FASTA format) of *TAGAP* and its amino acid substitutions were submitted. Output provides p-value where; *p* < 0.05 and *p* < 0.01 were considered as confident and very confident hypotheses, respectively.

### Analyzing protein stability

To check the stability of target protein, I-Mutant 2.0 was used which is a support vector machine based web server that helps in predicting any change in stability of protein after getting mutated. The tool uses data derived from ProTherm which is currently the most comprehensive database of experimental data on protein mutations. It predicts reliability index (RI) of the results ranging from 0–10, where 10 being the highest reliability [31,32]. We submitted *TAGAP* protein sequence to predict effects of the most damaging nsSNPs on the protein. Conditions for all submissions were set at temperature 25°C and pH 7.0.

### Analyzing protein evolutionary conservation

ConSurf is a bioinformatics tool that we used to estimate evolutionary conservation of amino acid positions using protein sequence [33]. Analysis is based on phylogenetic relations between homologous sequences [34–36]. Degree of conservation of amino acid residues was estimated using 50 homologous sequences. We selected those highly conserved residues for further analysis which were located at the sites of high risk nsSNPs.

### 3D protein modeling

The 3D models for wild type TAGAP protein and TAGAP mutated with high risk nsSNPs were generated using two homology modeling tools; Phyre2 and I-TASSER [37,38–40]. The resultant structures were viewed by Chimera 1.11 which is an extensive program for interactive visualization and analysis of molecular structures and related data [41] and later verified by ERRAT which is a program for verifying protein structures [42]. Afterwards TM-align was used to compare wild type protein structure with mutant protein structures. This algorithm computes template modeling-score (TM-score) and root mean square deviation (RMSD) along with superposition of the structures. TM-score gives the values in 0 and 1, where 1 indicates perfect match between two structures. While higher RMSD indicates greater variation between wild type and mutant structures [43,44].

### Predicting post translational modification (PTM) sites

The putative methylation sites in the *TAGAP* protein sequence were predicted by PSSMe and BPB-PPMS. The former tool identifies methylation sites based on information gain feature optimization method. The higher support vector machines (SVMs) probability indicates higher probability of lysine (or arginine) to get methylated. False-positive predictions were controlled by focusing on sites with stringency setting higher than 50% [45]. Methodology of BPB-PPMS is based on Bi-profile Bayes combined with SVMs having threshold value of 0.5 [46]. Likely phosphorylation sites in *TAGAP* protein at serine, threonine and tyrosine residues were predicted using NetPhos 3.1and GPS 3.0. NetPhos 3.1 uses ensembles of neural networks to complete this task and residues having scores >0.5 threshold are considered phosphorylated [47]. Likewise in GPS 3.0, higher value depicts higher potential of the residue to get phosphorylated [48]. Putative protein ubiquitylation sites were predicted by UbPred and BDM-PUB. In UbPred, lysine residues with a score of ≥ 0.62 were considered ubiquitylated [10] and in BDM-PUB, balanced cut-off option was selected [30].

## Results and discussion

### nsSNPs retrieved from dbSNP database

**Fig 1:**
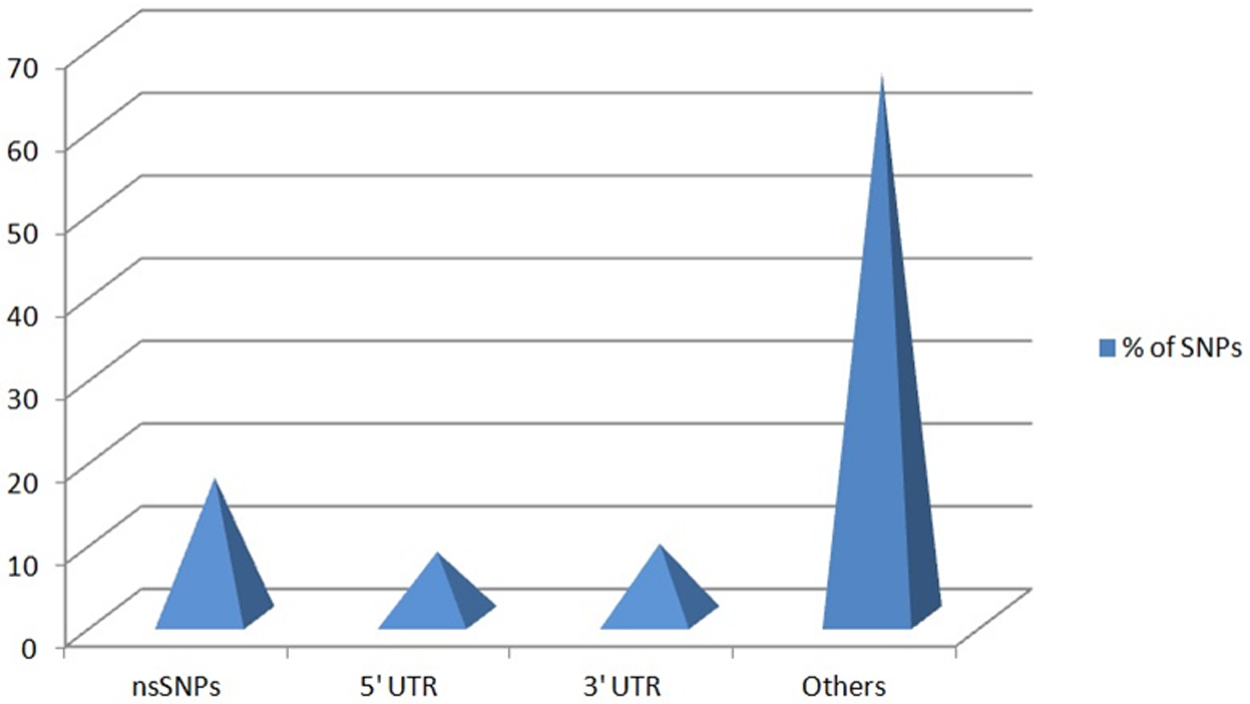
Clustered Pyramid showing the percentages of the SNPs in *TAGAP* gene. (nsSNPs: 17%; 5’UTR SNPs: 8%; 3’UTR SNPs: 9%; Other SNPs: 66%).

**Fig 2:**
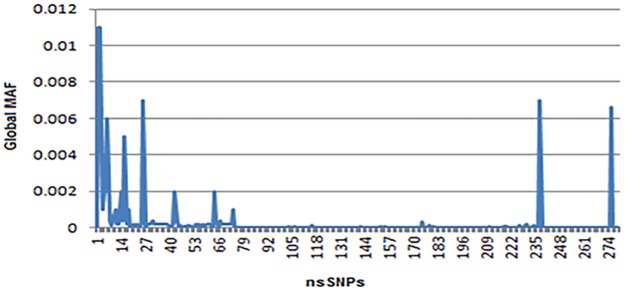
Graphical representation of global MAFs of nsSNPs.

We used dbSNP database to retrieve SNPs of interest as it is the most extensive SNP database [23]. There were total 1721 SNPs, of which 275 were nsSNPs, 147 occurred in 5’UTR, 162 in 3’UTR region, and the rest were of other types (Fig 1). We selected only nsSNPs for our investigation, details are provided in S1 Table. The global MAFs of these SNPs are shown in graphical form in Fig 2.

### Deleterious nsSNPs identified in *TAGAP*

**Fig 3:**
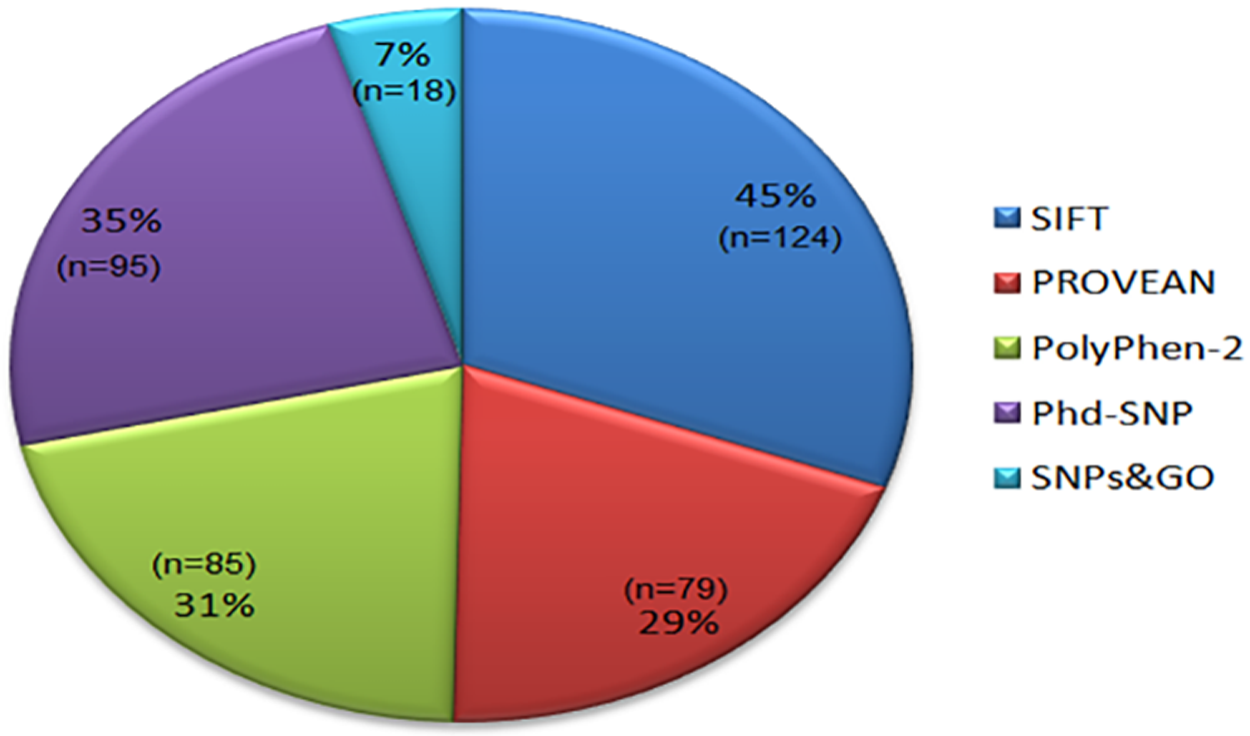
Pie chart showing percentage of damaging nsSNPs identified. The distribution of damaging nsSNPs by percentage (%) and number (n) identified by five *in silico* tools; SIFT, PROVEAN, PolyPhen-2, Phd-SNP and SNPs&GO.

We subjected all nsSNPs to five different *in silico* nsSNP prediction algorithms to investigate whether these SNPs have any effect on structure or function of *TAGAP* protein. *In silico* tools used for this purpose were: SIFT, PROVEAN, PolyPhen-2, PhD-SNP and SNPs&GO. According to SIFT results, nsSNPs scoring tolerance index (TI) of ≤0.05 are considered intolerant. In PROVEAN, the variants are predicted as deleterious when final score is below threshold value of -2.5 and neutral when it is above this value. PolyPhen-2 results predicted probably damaging, possibly damaging and benign nsSNPs, with probably damaging as being the most confident prediction as compared to other two. These predictions are based on position specific independent count score difference, where score 1 is considered the most damaging. The PhD-SNP predicted 95 nsSNPs as diseased while SNPs&GO revealed the most unique results showing only 18 nsSNPs as diseased (Fig 3, S2 Table).

We shortlisted those nsSNPs which are common in at least 4 of these algorithmic tools, and also which scored 0 in SIFT and 1 in PolyPhen-2 so that only the highly deleterious SNPs would be analyzed. Total 14 nsSNPs out of 275 met the criteria and we classified them as high risk. Interestingly, 9 of these nsSNPs lie in the only RhoGAP domain of *TAGAP* protein (Table 1) and all additional investigations were held for only these 9 nsSNPs.

**Table 1:** High risk nsSNPs identified by five *in silico* programs.

Amino acid Change	SIFT		PROVEAN		PolyPhen-2 (HumDiv)		PhD-SNP		SNPs&GO	
	Pred	TI	Sc	(cutoff = -2.5)	Effect	Sc	Pred	RI	Pred	RI
C94R	Intolerant	0	-11.11	Deleterious	Pro-damg	1	Diseased	7	Neutral	1
L100F*	Intolerant	0	-3.769	Deleterious	Pro-damg	1	Diseased	2	Neutral	7
T118M*	Intolerant	0	-5.67	Deleterious	Pro-damg	1	Diseased	4	Neutral	4
G120E*	Intolerant	0	-7.671	Deleterious	Pro-damg	1	Diseased	8	Diseased	4
F122L*	Intolerant	0	-5.753	Deleterious	Pro-damg	1	Diseased	7	Diseased	1
A126T*	Intolerant	0	-3.269	Deleterious	Pro-damg	1	Diseased	2	Neutral	8
E136K*	Intolerant	0	-3.702	Deleterious	Pro-damg	1	Diseased	0	Neutral	7
G141W*	Intolerant	0	-7.671	Deleterious	Pro-damg	1	Diseased	6	Diseased	0
V151M*	Intolerant	0	-2.596	Deleterious	Pro-damg	1	Diseased	2	Neutral	3
N205S*	Intolerant	0	-4.878	Deleterious	Pro-damg	1	Diseased	5	Diseased	3
S476F	Intolerant	0	-4.967	Deleterious	Pro-damg	1	Diseased	1	Neutral	8
S490P	Intolerant	0	-2.6	Deleterious	Pro-damg	1	Diseased	1	Diseased	2
F511S	Intolerant	0	-3.352	Deleterious	Pro-damg	1	Diseased	7	Neutral	2
F718S	Intolerant	0	-5.07	Deleterious	Pro-damg	1	Diseased	8	Diseased	3

* shows the positions in the RhoGAP domain region of the *TAGAP* protein,

Pred = prediction, Sc = score, Pro-damg = probably damaging.

### Functional and structural modifications of *TAGAP* predicted by MutPred

The shortlisted 9 nsSNPs were submitted to this server and the resultant probability scores are given in Table 2. The structural and functional alterations predicted include loss of disorder, catalytic residue, glycosylation and gain of phosphorylation, solvent accessibility, ubiquitination and molecular recognition features (MoRF) binding. Their P values are provided in S3 Table. According to these predictions, it can be stated that several nsSNPs might be the reason behind any possible structural and functional modifications of *TAGAP* protein.

**Table 2:** Probability scores of deleterious mutations.

Mutation	P-value	Mutation	P-value
T118M	0.618	G141W	0.663
L100F	0.573	V151M	0.676
F122L	0.846	A126T	0.804
G120E	0.902	E136K	0.498
N205S	0.896		

### Stability modification prediction

We predicted any stability alterations in the *TAGAP* protein with the help of I-Mutant which completes this task by considering the single-site mutations [21,32]. The 9 nsSNPs that have been found in the RhoGAP domain were submitted to I-Mutant 2.0 server to predict their RI and free energy change values. Results revealed that all these nsSNPs decrease stability of *TAGAP* protein (Table 3). Hence these polymorphisms in the RhoGAP domain might cause maximum damage to the protein by affecting its stability. According to some studies, decreased protein stability causes increase in degradation, misfolding and aggregation of proteins [49–51].

**Table 3:** I-MUTANT 2.0 and TM-align predictions for nsSNPs in RhoGAP domain of *TAGAP*.

nsSNP ID	Amino Acid Change	Stability	RI	DDG	TM-Score	RMSD
*rs748659041*	100, L → F	Decrease	9	-0.62	1	0
*rs368265576*	118, T → M	Decrease	6	-0.29	0.98778	0.83
*rs764717611*	120, G → E	Decrease	2	-0.84	0.78894	1.98
*rs763380333*	122, F → L	Decrease	3	-0.6	0.98778	0.83
*rs780953963*	126, A → T	Decrease	7	-1.04	0.7912	1.87
*rs866898464*	136, E → K	Decrease	7	-1.12	0.98778	0.83
*rs765146154*	141, G → W	Decrease	7	-0.58	0.78894	1.98
*rs777042268*	151, V → M	Decrease	9	-1.53	0.78851	1.99
*rs778438807*	205, N → S	Decrease	2	-2.45	0.7928	1.84

DDG: free energy change value. 0.0 < TM-score < 0.30, random structural similarity 0–0.3 and 0.5 < TM-score < 1.00, in about the same fold 0.5–1.

### Conservation profile of deleterious nsSNPs in *TAGAP*

Evolutionary information is essential to detect mutations which might affect human health [52]. Using ConSurf web server, we calculated the evolutionary conservation of amino acid residues of *TAGAP* protein to further explore the possible effects of 9 most deleterious nsSNPs. Results were obtained in the form of structural representation of the protein (S1 Fig). ConSurf identifies structural and functional residues by combining evolutionary conservation data with solvent accessibility predictions. Highly conserved residues are predicted as either functional or structural based on their location either on protein surface or inside its core [33]. Amino acids which are involved in vital biological processes for example in interactions among different proteins, are more conserved than others. Taking this into consideration, those nsSNPs which are located at these conserved regions are considered immensely damaging to protein as compared to those at non-conserved sites [9,53].

Results obtained via ConSurf represented all residues of *TAGAP* showing their structural and functional conservation levels. But we focused only on those residues which matched their positions with 9 high risk nsSNPs which we have identified. The results predicted L100, T118, E136, G141, V151 and N205 as functional residues making them highly conserved and exposed. While G120, F122 and A126 are predicted as structural residues which mean that they are highly conserved and buried (Table 4). The results further confirmed these 9 high risk nsSNPs as being really deleterious to the structure and/or function of *TAGAP* protein.

**Table 4:** ConSurf predictions showing conservation profile of amino acids in *TAGAP*.

SNP ID	Residue & Position	Conservation Score	Prediction
*rs748659041*	L100	8	Highly conserved and exposed (f)
*rs368265576*	T118	9	Highly conserved and exposed (f)
*rs764717611*	G120	9	Highly conserved and buried (s)
*rs763380333*	F122	9	Highly conserved and buried (s)
*rs780953963*	A126	9	Highly conserved and buried (s)
*rs866898464*	E136	9	Highly conserved and exposed (f)
*rs777042268*	G141	8	Highly conserved and exposed (f)
*rs778438807*	V151	9	Highly conserved and exposed (f)
*rs765146154*	N205	9	Highly conserved and exposed (f)

(f): predicted functional residue, (s): predicted structural residue.

### Comparative modeling of wild type *TAGAP* and its mutants

To determine whether the 9 high risk nsSNPs alter the wild type structure of *TAGAP* protein, we first used Phyre2 to generate 3D structures of wild type protein and 9 mutants. Each nsSNP was individually substituted into the wild type sequence of *TAGAP* and the sequences were submitted to Phyre2 homology modeling tool [37]. Phyre2 used c1xa6A as the template for predicting 3D models and the structures were then visualized by Chimera 1.11 [41]. We extended our analysis by calculating the TM-scores and RMSD values for each mutant model. The TM-score is used to evaluate the topological similarity between wild type and mutant models, while RMSD helps in measuring average distance between α-carbon backbones of wild type and mutant models [43–44]. The greater the RMSD value the greater is the deviation of mutant structure from that of the wild type (Table 3). The mutant model for V151M showed the maximum RMSD value of 1.99 followed by those of G120E and G141W having RMSD value 1.98. Interestingly, the mutant model L100F showed no variation from wild type structure as depicted by its RMSD value 0. The nsSNP models of T118M, F122L and E136K showed very slight variation from wild type *TAGAP* protein model (RMSD = 0.83).

**Fig 4:**
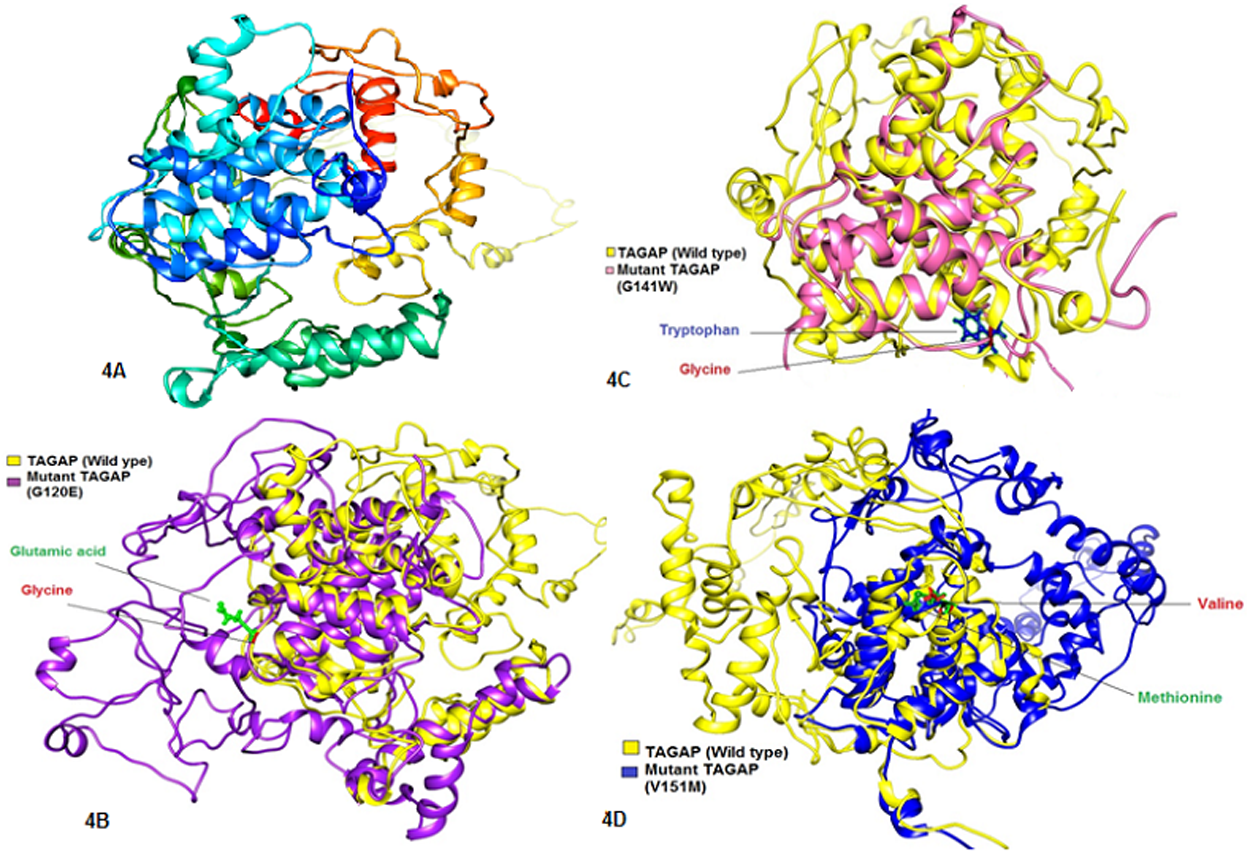
Comparison of wild type *TAGAP* protein structure with its mutant forms. (A) 3D model of wild type *TAGAP* protein. (B) Superimposed structures of wild type *TAGAP* protein and its mutant having mutation from Glycine to Glutamic Acid at position 120. (C) Superimposed structures of wild type *TAGAP* protein and its mutant having mutation from Glycine to Tryptophan at position 141. (D) Superimposed structures of wild type *TAGAP* protein and its mutant having mutation from Valine to Methionine at position 151.

Based on higher RMSD values, we finally selected only three mutants; G120E, G141W and V151M to remodel them using I-TASSER (being the most advanced modeling tool) [38–41] so that we could come up with the most reliable protein structures. The templates used by this server were: 5c5sA, 3cxlA, 3flc2A, 3msxB and 5ircA. I-TASSER produced 5 models each for *TAGAP* and its mutants. We selected only that model having minimum C-score and also had higher ERRAT values when submitted to ERRAT program (G120E; C-score = -2.95, ERRAT = 84, G141W; C-score = -3.01, ERRAT = 91.6, V151M; C-score = -2.95, ERRAT = 89.4). Hence these three mutant models were finally superimposed over the wild type protein model (Fig 4). Similar studies have been carried out on various genes and proteins like GDH protein, *MBL2* gene etc using different bioinformatics tools [54,55]. Such studies can offer novel therapeutic markers for a range of diseases.

### Predicted post translational modifications

PTMs are important in regulating structures and functions of proteins hence are involved in many biological events, for example, protein-protein interactions and cell signaling etc [56,57]. In this study, we sought to investigate whether the high risk nsSNPs have any effect on PTMs in *TAGAP*. For this, we used various *in silico* tools to predict probable PTM sites in the *TAGAP* protein.

#### Methylation

**Fig 5:**
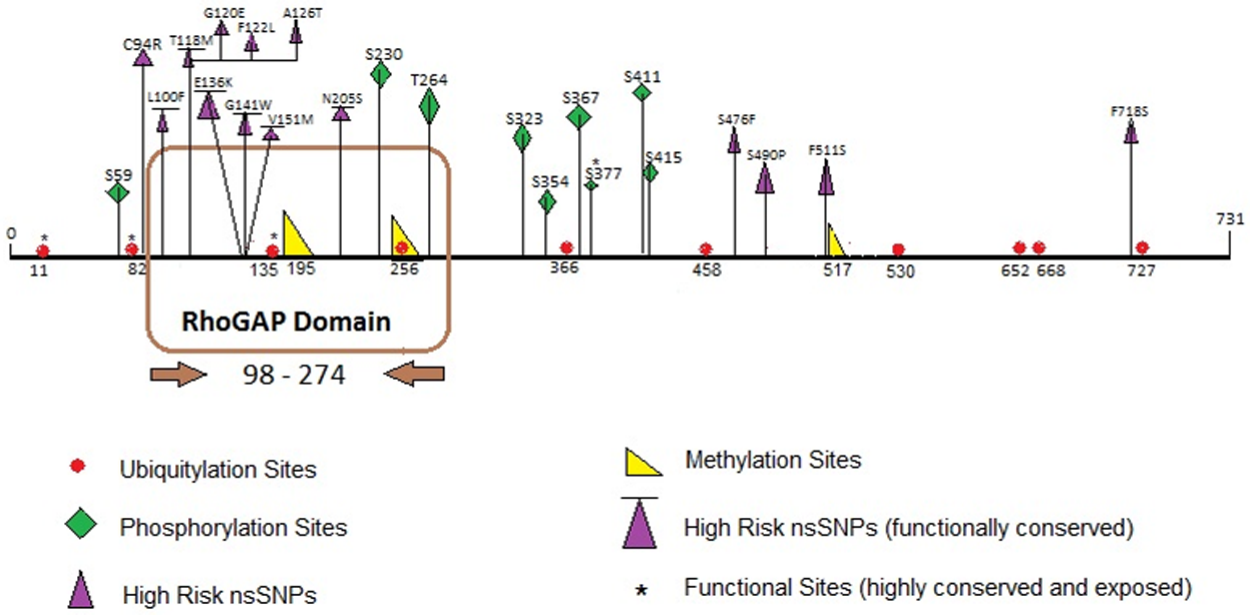
Putative PTM sites and high risk nsSNPs in *TAGAP* protein. Schematic illustration of locations of high risk nsSNPs and PTMs (ubiquitylation, phosphorylation and methylation) in *TAGAP* protein. The nsSNPs that are also predicted as functionally conserved by ConSurf are all present in the RhoGAP domain of the protein starting from L100F to N205S. The site K256 is predicted to undergo both methylation and ubiquitylation.

Methylation of lysine residues in certain histones effect their binding with the neighboring DNA and this alters the expression of genes on that DNA. The PSSMe tool predicted a total of 21 lysine residue sites that can get methylated, whereas BPB-PPMS predicted only 6 residues that undergo methylation. Only 3 lysine residues at positions 195, 256 and 517 were common findings of both PSSMe and BPB-PPMS tools (Fig 5).

#### Phosphorylation

Phosphorylation of proteins is an important regulatory mechanism as it acts as their molecular switch to perform various functions like conformational changes in protein structure, during signal transduction pathways, activating some proteins and deactivating others [58–61]. The putative phosphorylation sites in the *TAGAP* were predicted by using NetPhos 3.1 and GPS 3.0 servers which made predictions for 17 and 3 kinases respectively (S4 Table). NetPhos 3.1 predicted total 97 residues (Ser:76, Thr:18, Tyr:3) having potential of getting phosphorylated. On the contrary, GPS 3.0 predicted only 10 residues, of which 9 were seriene specific sites and 1 was threonine specific phosphorylation site. No phosphorylation at tyrosine residues was predicted by GPS 3.0. The common sites predicted by both servers are shown in Fig 5. According to ConSurf results, only S377 of these sites is a highly conserved and exposed residue making it a functional residue hence depicting its significance.

#### Ubiquitylation

Ubiquitylation is a PTM which serves as a degradation mechanism for proteins and helps in DNA damage repair [62]. These modification sites in the *TAGAP* were predicted by UbPred and BDM-PUB tools. UbPred predicted 12 lysine residues in the *TAGAP* protein that experience ubiquitylation while BDM-PUB predicted that 33 lysine residues undergo ubiquitylation. Total 10 residues were common predictions by both UbPred and BDM-PUB (Fig 5). Only 3 of these putative ubiquitylation sites; K11, K82 and K135 were predicted to be important functional residues (highly conserved and exposed) according to the ConSurf results (S1 Fig). All putative PTM sites in *TAGAP* protein along with the high risk nsSNPs identified in this study are illustrated in Fig 5 while the results of all the PTMs are provided in S4 Table.

A few of these PTMs also coincided in position with nsSNPs (S1 Table) in the *TAGAP*, i.e. K82, S367 and S411 (Table 5) and of these, K82 is highly conserved among *TAGAP* homologues according to the ConSurf results.

**Table 5:** Low risk nsSNPs identified considering PTMs and ConSurf predictions.

SNP ID	Mutation	Deleterious Predictions	PTM	ConSurf Prediction
*rs375785212*	K82N	3	Ubiquitylation	F
*rs776525307*	S367I	4	Phosphorylation	E
*rs182059529*	S411C	1	Phosphorylation	E

e: exposed residue (highly conserved and buried), f: functional residue (highly conserved and exposed).

Though the upshots of *TAGAP* methylation, phosphorylation and ubiquitylation have not been reported yet, various studies have shown that these modifications can significantly alter the protein function by varying its location, stability or inter-protein interactions etc. It is possible that ubiquitylation of lysine residues in the *TAGAP* at sites K52, K82, K114 and K518 and phosphorylation of serine residues at sites S367 and S411 are vital for some of the protein’s essential functions, and that the missense SNPs: K52T, K82N, K114E, K518T, S367I and S411C somehow impair those functions. Conversely, these nsSNPs might also destabilize the protein that might eventually enhance the harms of PTM impairment.

## Conclusions

This study suggests that structure and/or function of *TAGAP* protein can be disturbed by various nsSNPs. In native protein of *TAGAP* gene, three major mutations found were: Glycine → Glutamic Acid at position 120 (*rs764717611*), Glycine → Tryptophan at position 141 (*rs777042268*) and Valine → Methionine at position 151 (*rs778438807*). These mutations occur in RhoGAP domain of *TAGAP* protein hence are of particular concern as this is the only functional domain of the protein. Therefore, these nsSNPs can be strongly considered as key candidates in causing diseases related to *TAGAP* malfunction and hence will help in effective drug discovery and developing precision medicines. Thorough investigations and wet lab experimentation are needed to explore the effects of these polymorphisms on structure and function of the protein. Also, various diseased animal models comprising these major mutations in the *TAGAP* protein might be very supportive in exploring their job in the disease.

## Supporting information

S1 Fig ConSurf prediction showing conservation profile of amino acids in *TAGAP* protein. (PDF)

S1 Table All 275 nsSNPs including their ID, allele change, protein accession number, position, amino acid change and global maf. (DOCX)

S2 Table The results for all 275 nsSNPs by five *in silico* tools. (DOCX)

S3 Table Effects of nsSNPs on structural & functional properties of *TAGAP* by MutPred server. (DOCX)

S4 Table Post translational modifications. (DOCX)
