# Supplementary material for: Identification and in silico analysis of functional SNPs of human TAGAP protein: A comprehensive study
Source: PLoS One. 2018 Jan 12;13(1):e0188143. doi: 10.1371/journal.pone.0188143 (PMC5766082; doi:10.1371/journal.pone.0188143)
Supplement: S1 Fig — (PDF) [file pone.0188143.s001.pdf]

ConSurf Results

Supplementary Figure 1. Consurf Prediction showing Conservation Profile of Amino Acids in TAGAP protein

```
1                     11                    21                    31                    41
M K L R S S H N A S   K T L N A N N M E T   L I E C Q S E G D I   K E H P L L A S C E   S E D S I C Q L I E
e e e e e e e e b e   e e b e e e e b e b   b b e b e e e e e b   e e b e b b b e b e   e e e b b b e b b e
f f f   f             f f             s f         s   f f         f             f       f f f             f

51                    61                    71                    81                    91
V K K R K K V L S W   P F L M R R L S P A   S D F S G A L E T D   L K A S L F D Q P L   S I I C G D S D T L
b e e e e e e e e b   b b b b e e b e e e   e e b e e e b e e e   b e e e b b e e e b   b b b b e e e e e e
  f f f f f f     s           f             f f           f         f     s s f f f s         s           f

101                   111                   121                   131                   141
P R P I Q D I L T I   L C L K G P S T E G   I F R R A A N E K A   R K E L K E E L N S   G D A V D L E R L P
e e e b e e b b b b   b b b e b e e e e b   b b e e b b e e e b   e e e b e e e b e e   e e e b e b e e b e
f   f s f f s s       s     f s f f f f s   s s f f s s f f f s   f f f s f f   s f     f     s           f

151                   161                   171                   181                   191
V H L L A V V F K D   F L R S I P R K L L   S S D L F E E W M G   A L E M Q D E E D R   I E A L K Q V A D K
e e b b b b b b e e   b b e e b b e e b b   b b e b b e e b b e   b b e e e e e e e e   b e b b e e b b e e
f f s s s   s   f f   s s f   s s   f s s   s     s       s s     s           f     f   s f   s     s

201                   211                   221                   231                   241
L P R P N L L L L K   H L V Y V L H L I S   K N S E V N R M D S   S N L A I C I G P N   M L T L E N D Q S L
e e e e e b e b b e   e b b b b b b b b b   e e e e b e e b e b   e e b b b b b b e e   b b b e e e e e e b
f f   f f     s s     f       s s       s     f           s f     f f s s s s   s f f     s

251                   261                   271                   281                   291
S F E A Q K D L N N   K V K T L V E F L I   D N C F E I F G E N   I P V H S S I T S D   D S L E H T D S S D
e b e b e e e b e e   e b e e b b e b b b   e e b b e b b b e e   b e e e b e b b b e   e b b e e e e e e e
f   f s f       f     f s f   s s f s   s   f f     f s     f                     s f     s   f f f f f f f

301                   311                   321                   331                   341
V S T L Q N D S A Y   D S N D P D V E S N   S S S G I S S P S R   Q P Q V P M A T A A   G L D S A G P Q D A
b b e b e e e e e e   e e e e e e e e e e   e e e e e e e e e e   e e e e e e e e b e   e e e e e e e e e e
  s f   f f f f f f   f f f f f f

351                   361                   371                   381                   391
R E V S P E P I V S   T V A R L K S S L A   Q P D R R Y S E P S   M P S S Q E C L E S   R V T N Q T L T K S
e e e e e e e b b b   b b b e b e e e b e   e e e e e e e e e e   e e e b e e b b e e   e e e e e e b e e e
                                                f f f   f f f                                             f

401                   411                   421                   431                   441
E G D F P V P R V G   S R L E S E E A E D   P F P E E V F P A V   Q G K T K R P V D L   K I K N L A P G S V
e e e b e b e e e b   e e b e e e e e e e   e b e e e b b e e b   e e e e e e e e e b   e b e e b e e e b e
  f                                     f         f     s

451                   461                   471                   481                   491
L P R A L V L K A F   S S S S L D A S S D   S S P V A S P S S P   K R N F F S R H Q S   F T T K T E K G K P
b e e e e b e e b b   e e b e b e e e e e   e e e e e e e e e e   e e e b b e e e e e   b e e e e e e e e e
                      f     f                 f f     f f f         f         f f f f   s     f         f

501                   511                   521                   531                   541
S R E I K K H S M S   F T F A P H K K V L   T K N L S A G S G K   S Q D F T R D H V P   R G V R K E S Q L A
e e e b e e e b b b   b b b b e e e e b e   e e e e e e e e e e   e e e e e e e e b e   e e e e e e b e e b
  f f     f f s   s   s s       f                   f                                                 f f

551                   561                   571                   581                   591
G R I V Q E N G C E   T H N Q T A R G F C   L R P H A L S V D D   V F Q G A D W E R P   G S P P S Y E E A M
e e b b e e e e e e   e e e e b b e e b e   b e b b b b e b e e   b b e e b e e e e e   e e e e e e e e b b
f f       f                                             f         s s                       f f f f   f s
```

```
601        611        621        631        641
QGPAARLVAS ESQTVGSMTV GSMRARMLEA HCLLPPLPPA HHVEDSRHRG
ebebbebeee eeeebeebbb bbbebbbbee eeeeeeeebe eeeeeeeeee
             ffs f       f

651        661        671        681        691
SKEPLPGHGL SPLPERWKQS RTVHASGDSL GHVSGPGRPE LLPLRTVSES
eeeeeeeeeb beeeeeeeeb eebbbbbeee eebebeeeee beebeeeeee
                       f                        ff ff

701        711        721        731
VQRNKRDCLV RRCSQPVFEA DQFQYAKESY I
eeeeeeebbe eebeeebbeb eebeebeeee e
              ff       f     fff f
```

The conservation scale:

1	2	3	4	5	6	7	8	9

Variable Average Conserved

e - An exposed residue according to the .

b - A buried residue according to the .

f - A predicted functional residue (highly conserved and exposed).

s - A predicted structural residue (highly conserved and buried).

X - Insufficient data - the calculation for this site was performed on less than 10% of the sequences.
