# Supplementary material for: Identification and in silico analysis of functional SNPs of human TAGAP protein: A comprehensive study
Source: PLoS One. 2018 Jan 12;13(1):e0188143. doi: 10.1371/journal.pone.0188143 (PMC5766082; doi:10.1371/journal.pone.0188143)
Supplement: S1 Table — (DOCX) [file pone.0188143.s002.docx]

| **SNP ID** | **Allele Change** | **Protein Accession** | **Position** | **Residue Change**  **All 275 nsSNPs including their ID, allele change, protein accession number, its position, amino acid change and global maf.** | **Global MAF** |
| --- | --- | --- | --- | --- | --- |
| [rs35263580](https://www.ncbi.nlm.nih.gov/projects/SNP/snp_ref.cgi?rs=35263580) | GGC ⇒ GAC | [NP_473455.2](https://www.ncbi.nlm.nih.gov/entrez/query.fcgi?cmd=Search&db=protein&term=NP_473455.2) | 346 | G [Gly] ⇒ D [Asp] | 0.011 |
| [rs41267765](https://www.ncbi.nlm.nih.gov/projects/SNP/snp_ref.cgi?rs=41267765) | GAG ⇒ AAG | [NP_473455.2](https://www.ncbi.nlm.nih.gov/entrez/query.fcgi?cmd=Search&db=protein&term=NP_473455.2) | 147 | E [Glu] ⇒ K [Lys] | 0.011 |
| [rs75182303](https://www.ncbi.nlm.nih.gov/projects/SNP/snp_ref.cgi?rs=75182303) | ACA ⇒ TCA | [NP_473455.2](https://www.ncbi.nlm.nih.gov/entrez/query.fcgi?cmd=Search&db=protein&term=NP_473455.2) | [396](https://www.ncbi.nlm.nih.gov/sites/protein/NP_001265662.1?report=graph&v=283:383&content=5&m=333!&mn=rs75182303&dispmax=1&currpage=1) | T [Thr] ⇒ S [Ser] | 0.001 |
| [rs112777023](https://www.ncbi.nlm.nih.gov/projects/SNP/snp_ref.cgi?rs=112777023) | CCA ⇒ ACA | [NP_473455.2](https://www.ncbi.nlm.nih.gov/entrez/query.fcgi?cmd=Search&db=protein&term=NP_473455.2) | [347](https://www.ncbi.nlm.nih.gov/sites/protein/NP_001265662.1?report=graph&v=234:334&content=5&m=284!&mn=rs112777023&dispmax=1&currpage=1) | P [Pro] ⇒ T [Thr] | 0.002 |
| [rs112913154](https://www.ncbi.nlm.nih.gov/projects/SNP/snp_ref.cgi?rs=112913154) | CAT ⇒ TAT | [NP_473455.2](https://www.ncbi.nlm.nih.gov/entrez/query.fcgi?cmd=Search&db=protein&term=NP_473455.2) | [284](https://www.ncbi.nlm.nih.gov/sites/protein/NP_001265662.1?report=graph&v=171:271&content=5&m=221!&mn=rs112913154&dispmax=1&currpage=1) | H [His] ⇒ Y [Tyr] | 0.002 |
| [rs116639718](https://www.ncbi.nlm.nih.gov/projects/SNP/snp_ref.cgi?rs=116639718) | GGT ⇒ GAT | [NP_473455.2](https://www.ncbi.nlm.nih.gov/entrez/query.fcgi?cmd=Search&db=protein&term=NP_473455.2) | 542 | G [Gly] ⇒ D [Asp] | 0.006 |
| [rs138323645](https://www.ncbi.nlm.nih.gov/projects/SNP/snp_ref.cgi?rs=138323645) | CCT ⇒ TCT | [NP_473455.2](https://www.ncbi.nlm.nih.gov/entrez/query.fcgi?cmd=Search&db=protein&term=NP_473455.2) | [428](https://www.ncbi.nlm.nih.gov/sites/protein/NP_001265662.1?report=graph&v=315:415&content=5&m=365!&mn=rs138323645&dispmax=1&currpage=1) | P [Pro] ⇒ S [Ser] | 0.0004 |
| [rs139309613](https://www.ncbi.nlm.nih.gov/projects/SNP/snp_ref.cgi?rs=139309613) | GCG ⇒ ACG | [NP_473455.2](https://www.ncbi.nlm.nih.gov/entrez/query.fcgi?cmd=Search&db=protein&term=NP_473455.2) | [345](https://www.ncbi.nlm.nih.gov/sites/protein/NP_001265662.1?report=graph&v=232:332&content=5&m=282!&mn=rs139309613&dispmax=1&currpage=1) | A [Ala] ⇒ T [Thr] | 0.0001 |
| [rs139378811](https://www.ncbi.nlm.nih.gov/projects/SNP/snp_ref.cgi?rs=139378811) | GAG ⇒ AAG | [NP_473455.2](https://www.ncbi.nlm.nih.gov/entrez/query.fcgi?cmd=Search&db=protein&term=NP_473455.2) | [192](https://www.ncbi.nlm.nih.gov/sites/protein/NP_001265662.1?report=graph&v=79:179&content=5&m=129!&mn=rs139378811&dispmax=1&currpage=1) | E [Glu] ⇒ K [Lys] | 0.0004 |
| [rs139559821](https://www.ncbi.nlm.nih.gov/projects/SNP/snp_ref.cgi?rs=139559821) | TGC ⇒ GGC | [NP_473455.2](https://www.ncbi.nlm.nih.gov/entrez/query.fcgi?cmd=Search&db=protein&term=NP_473455.2) | [632](https://www.ncbi.nlm.nih.gov/sites/protein/NP_001265662.1?report=graph&v=519:619&content=5&m=569!&mn=rs139559821&dispmax=1&currpage=1) | C [Cys] ⇒ G [Gly] | 0.001 |
| [rs140113362](https://www.ncbi.nlm.nih.gov/projects/SNP/snp_ref.cgi?rs=140113362) | AAG ⇒ ACG | [NP_473455.2](https://www.ncbi.nlm.nih.gov/entrez/query.fcgi?cmd=Search&db=protein&term=NP_473455.2) | [52](https://www.ncbi.nlm.nih.gov/sites/protein/NP_473455.2?report=graph&v=2:102&content=5&m=52!&mn=rs140113362&dispmax=1&currpage=1) | K [Lys] ⇒ T [Thr] | 0.0002 |
| [rs140990019](https://www.ncbi.nlm.nih.gov/projects/SNP/snp_ref.cgi?rs=140990019) | AGT ⇒ CGT | [NP_473455.2](https://www.ncbi.nlm.nih.gov/entrez/query.fcgi?cmd=Search&db=protein&term=NP_473455.2) | [44](https://www.ncbi.nlm.nih.gov/sites/protein/NP_473455.2?report=graph&v=1:94&content=5&m=44!&mn=rs140990019&dispmax=1&currpage=1) | S [Ser] ⇒ R [Arg] | 0.0002 |
| [rs142920707](https://www.ncbi.nlm.nih.gov/projects/SNP/snp_ref.cgi?rs=142920707) | GCA ⇒ GTA | [NP_473455.2](https://www.ncbi.nlm.nih.gov/entrez/query.fcgi?cmd=Search&db=protein&term=NP_473455.2) | [604](https://www.ncbi.nlm.nih.gov/sites/protein/NP_001265662.1?report=graph&v=491:591&content=5&m=541!&mn=rs142920707&dispmax=1&currpage=1) | [A [Ala] ⇒ V [Val]](https://www.ncbi.nlm.nih.gov/sites/protein/NP_001265662.1?report=graph&v=491:591&content=5&m=541!&mn=rs142920707&dispmax=1&currpage=1) | 0.002 |
| [rs142994072](https://www.ncbi.nlm.nih.gov/projects/SNP/snp_ref.cgi?rs=142994072) | GAG ⇒ GAC | [NP_473455.2](https://www.ncbi.nlm.nih.gov/entrez/query.fcgi?cmd=Search&db=protein&term=NP_473455.2) | [187](https://www.ncbi.nlm.nih.gov/sites/protein/NP_001265662.1?report=graph&v=74:174&content=5&m=124!&mn=rs142994072&dispmax=1&currpage=1) | E [Glu] ⇒ D [Asp] | 0.0004 |
| [rs144047559](https://www.ncbi.nlm.nih.gov/projects/SNP/snp_ref.cgi?rs=144047559) | GGC ⇒ GCC | [NP_473455.2](https://www.ncbi.nlm.nih.gov/entrez/query.fcgi?cmd=Search&db=protein&term=NP_473455.2) | [410](https://www.ncbi.nlm.nih.gov/sites/protein/NP_001265662.1?report=graph&v=297:397&content=5&m=347!&mn=rs144047559&dispmax=1&currpage=1) | G [Gly] ⇒ A [Ala] | 0.005 |
| [rs144116675](https://www.ncbi.nlm.nih.gov/projects/SNP/snp_ref.cgi?rs=144116675) | ACG ⇒ GCG | [NP_473455.2](https://www.ncbi.nlm.nih.gov/entrez/query.fcgi?cmd=Search&db=protein&term=NP_473455.2) | [118](https://www.ncbi.nlm.nih.gov/sites/protein/NP_001265662.1?report=graph&v=5:105&content=5&m=55!&mn=rs144116675&dispmax=1&currpage=1) | T [Thr] ⇒ A [Ala] | 0.0002 |
| [rs144641395](https://www.ncbi.nlm.nih.gov/projects/SNP/snp_ref.cgi?rs=144641395) | AAA ⇒ ACA | [NP_473455.2](https://www.ncbi.nlm.nih.gov/entrez/query.fcgi?cmd=Search&db=protein&term=NP_473455.2) | [518](https://www.ncbi.nlm.nih.gov/sites/protein/NP_001265662.1?report=graph&v=405:505&content=5&m=455!&mn=rs144641395&dispmax=1&currpage=1) | K [Lys] ⇒ T [Thr] | 0.001 |
| [rs144691673](https://www.ncbi.nlm.nih.gov/projects/SNP/snp_ref.cgi?rs=144691673) | TAT ⇒ TGT | [NP_473455.2](https://www.ncbi.nlm.nih.gov/entrez/query.fcgi?cmd=Search&db=protein&term=NP_473455.2) | [725](https://www.ncbi.nlm.nih.gov/sites/protein/NP_001265662.1?report=graph&v=612:712&content=5&m=662!&mn=rs144691673&dispmax=1&currpage=1) | Y [Tyr] ⇒ C [Cys] | 0.000008 |
| [rs144719803](https://www.ncbi.nlm.nih.gov/projects/SNP/snp_ref.cgi?rs=144719803) | GTG ⇒ GGG | [NP_473455.2](https://www.ncbi.nlm.nih.gov/entrez/query.fcgi?cmd=Search&db=protein&term=NP_473455.2) | [359](https://www.ncbi.nlm.nih.gov/sites/protein/NP_001265662.1?report=graph&v=246:346&content=5&m=296!&mn=rs144719803&dispmax=1&currpage=1) | V [Val] ⇒ G [Gly] | 0.00008 |
| [rs144830124](https://www.ncbi.nlm.nih.gov/projects/SNP/snp_ref.cgi?rs=144830124) | CGG ⇒ CAG | [NP_473455.2](https://www.ncbi.nlm.nih.gov/entrez/query.fcgi?cmd=Search&db=protein&term=NP_473455.2) | [706](https://www.ncbi.nlm.nih.gov/sites/protein/NP_001265662.1?report=graph&v=593:693&content=5&m=643!&mn=rs144830124&dispmax=1&currpage=1) | R [Arg] ⇒ Q [Gln] | 0.0002 |
| [rs145572935](https://www.ncbi.nlm.nih.gov/projects/SNP/snp_ref.cgi?rs=145572935) | GTG ⇒ ATG | [NP_473455.2](https://www.ncbi.nlm.nih.gov/entrez/query.fcgi?cmd=Search&db=protein&term=NP_473455.2) | [554](https://www.ncbi.nlm.nih.gov/sites/protein/NP_001265662.1?report=graph&v=441:541&content=5&m=491!&mn=rs145572935&dispmax=1&currpage=1) | V [Val] ⇒ M [Met] | 0.00008 |
| [rs146522698](https://www.ncbi.nlm.nih.gov/projects/SNP/snp_ref.cgi?rs=146522698) | CCT ⇒ GCT | [NP_473455.2](https://www.ncbi.nlm.nih.gov/entrez/query.fcgi?cmd=Search&db=protein&term=NP_473455.2) | [315](https://www.ncbi.nlm.nih.gov/sites/protein/NP_001265662.1?report=graph&v=202:302&content=5&m=252!&mn=rs146522698&dispmax=1&currpage=1) | P [Pro] ⇒ A [Ala] | 0.0002 |
| [rs146899715](https://www.ncbi.nlm.nih.gov/projects/SNP/snp_ref.cgi?rs=146899715) | GTG ⇒ GCG | [NP_473455.2](https://www.ncbi.nlm.nih.gov/entrez/query.fcgi?cmd=Search&db=protein&term=NP_473455.2) | [215](https://www.ncbi.nlm.nih.gov/sites/protein/NP_001265662.1?report=graph&v=102:202&content=5&m=152!&mn=rs146899715&dispmax=1&currpage=1) | V [Val] ⇒ A [Ala] | 0.00008 |
| [rs148545546](https://www.ncbi.nlm.nih.gov/projects/SNP/snp_ref.cgi?rs=148545546) | GCC ⇒ GAC | [NP_473455.2](https://www.ncbi.nlm.nih.gov/entrez/query.fcgi?cmd=Search&db=protein&term=NP_473455.2) | [350](https://www.ncbi.nlm.nih.gov/sites/protein/NP_001265662.1?report=graph&v=237:337&content=5&m=287!&mn=rs148545546&dispmax=1&currpage=1) | A [Ala] ⇒ D [Asp] | 0.0002 |
| [rs148570036](https://www.ncbi.nlm.nih.gov/projects/SNP/snp_ref.cgi?rs=148570036) | CAT ⇒ CGT | [NP_473455.2](https://www.ncbi.nlm.nih.gov/entrez/query.fcgi?cmd=Search&db=protein&term=NP_473455.2) | [674](https://www.ncbi.nlm.nih.gov/sites/protein/NP_001265662.1?report=graph&v=561:661&content=5&m=611!&mn=rs148570036&dispmax=1&currpage=1) | H [His] ⇒ R [Arg] | 0.007 |
| [rs148912994](https://www.ncbi.nlm.nih.gov/projects/SNP/snp_ref.cgi?rs=148912994) | GAG ⇒ AAG | [NP_473455.2](https://www.ncbi.nlm.nih.gov/entrez/query.fcgi?cmd=Search&db=protein&term=NP_473455.2) | [588](https://www.ncbi.nlm.nih.gov/sites/protein/NP_001265662.1?report=graph&v=475:575&content=5&m=525!&mn=rs148912994&dispmax=1&currpage=1) | [E [Glu] ⇒ K [Lys]](https://www.ncbi.nlm.nih.gov/sites/protein/NP_001265662.1?report=graph&v=475:575&content=5&m=525!&mn=rs148912994&dispmax=1&currpage=1) | 0.00002 |
| [rs149576847](https://www.ncbi.nlm.nih.gov/projects/SNP/snp_ref.cgi?rs=149576847) | CGT ⇒ CAT | [NP_473455.2](https://www.ncbi.nlm.nih.gov/entrez/query.fcgi?cmd=Search&db=protein&term=NP_473455.2) | [131](https://www.ncbi.nlm.nih.gov/sites/protein/NP_001265662.1?report=graph&v=18:118&content=5&m=68!&mn=rs149576847&dispmax=1&currpage=1) | R [Arg] ⇒ H [His] | 0.0002 |
| [rs150468963](https://www.ncbi.nlm.nih.gov/projects/SNP/snp_ref.cgi?rs=150468963) | GCC ⇒ CCC | [NP_473455.2](https://www.ncbi.nlm.nih.gov/entrez/query.fcgi?cmd=Search&db=protein&term=NP_473455.2) | [459](https://www.ncbi.nlm.nih.gov/sites/protein/NP_001265662.1?report=graph&v=346:446&content=5&m=396!&mn=rs150468963&dispmax=1&currpage=1) | A [Ala] ⇒ P [Pro] | 0.0002 |
| [rs180730813](https://www.ncbi.nlm.nih.gov/projects/SNP/snp_ref.cgi?rs=180730813) | TTG ⇒ TCG | [NP_473455.2](https://www.ncbi.nlm.nih.gov/entrez/query.fcgi?cmd=Search&db=protein&term=NP_473455.2) | [81](https://www.ncbi.nlm.nih.gov/sites/protein/NP_001265662.1?report=graph&v=1:68&content=5&m=18!&mn=rs180730813&dispmax=1&currpage=1) | L [Leu] ⇒ S [Ser] | 0.0002 |
| [rs181275709](https://www.ncbi.nlm.nih.gov/projects/SNP/snp_ref.cgi?rs=181275709) | TCA ⇒ CCA | [NP_473455.2](https://www.ncbi.nlm.nih.gov/entrez/query.fcgi?cmd=Search&db=protein&term=NP_473455.2) | [26](https://www.ncbi.nlm.nih.gov/sites/protein/NP_473455.2?report=graph&v=1:76&content=5&m=26!&mn=rs181275709&dispmax=1&currpage=1) | S [Ser] ⇒ P [Pro] | 0.0004 |
| [rs182059529](https://www.ncbi.nlm.nih.gov/projects/SNP/snp_ref.cgi?rs=182059529) | TCT ⇒ TGT | [NP_473455.2](https://www.ncbi.nlm.nih.gov/entrez/query.fcgi?cmd=Search&db=protein&term=NP_473455.2) | [411](https://www.ncbi.nlm.nih.gov/sites/protein/NP_001265662.1?report=graph&v=298:398&content=5&m=348!&mn=rs182059529&dispmax=1&currpage=1) | S [Ser] ⇒ C [Cys] | 0.0002 |
| [rs188045239](https://www.ncbi.nlm.nih.gov/projects/SNP/snp_ref.cgi?rs=188045239) | GTC ⇒ GCC | [NP_473455.2](https://www.ncbi.nlm.nih.gov/entrez/query.fcgi?cmd=Search&db=protein&term=NP_473455.2) | [673](https://www.ncbi.nlm.nih.gov/sites/protein/NP_001265662.1?report=graph&v=560:660&content=5&m=610!&mn=rs188045239&dispmax=1&currpage=1) | V [Val] ⇒ A [Ala] | 0.0002 |
| [rs193056043](https://www.ncbi.nlm.nih.gov/projects/SNP/snp_ref.cgi?rs=193056043) | CCT ⇒ GCT | [NP_473455.2](https://www.ncbi.nlm.nih.gov/entrez/query.fcgi?cmd=Search&db=protein&term=NP_473455.2) | [636](https://www.ncbi.nlm.nih.gov/sites/protein/NP_001265662.1?report=graph&v=523:623&content=5&m=573!&mn=rs193056043&dispmax=1&currpage=1) | P [Pro] ⇒ A [Ala] | 0.0002 |
| [rs199576594](https://www.ncbi.nlm.nih.gov/projects/SNP/snp_ref.cgi?rs=199576594) | TTT ⇒ CTT | [NP_473455.2](https://www.ncbi.nlm.nih.gov/entrez/query.fcgi?cmd=Search&db=protein&term=NP_473455.2) | [158](https://www.ncbi.nlm.nih.gov/sites/protein/NP_001265662.1?report=graph&v=45:145&content=5&m=95!&mn=rs199576594&dispmax=1&currpage=1) | F [Phe] ⇒ L [Leu] | 0.0002 |
| rs200038955 | GAG ⇒ AAG | [NP_473455.2](https://www.ncbi.nlm.nih.gov/entrez/query.fcgi?cmd=Search&db=protein&term=NP_473455.2) | 187 | E [Glu] ⇒ K [Lys] | 0.0002 |
| [rs200734731](https://www.ncbi.nlm.nih.gov/projects/SNP/snp_ref.cgi?rs=200734731) | GTG ⇒ ATG | [NP_473455.2](https://www.ncbi.nlm.nih.gov/entrez/query.fcgi?cmd=Search&db=protein&term=NP_473455.2) | [362](https://www.ncbi.nlm.nih.gov/sites/protein/NP_001265662.1?report=graph&v=249:349&content=5&m=299!&mn=rs200734731&dispmax=1&currpage=1) | V [Val] ⇒ M [Met] | 0.0002 |
| [rs201023356](https://www.ncbi.nlm.nih.gov/projects/SNP/snp_ref.cgi?rs=201023356) | CGG ⇒ CAG | [NP_473455.2](https://www.ncbi.nlm.nih.gov/entrez/query.fcgi?cmd=Search&db=protein&term=NP_473455.2) | [167](https://www.ncbi.nlm.nih.gov/sites/protein/NP_001265662.1?report=graph&v=54:154&content=5&m=104!&mn=rs201023356&dispmax=1&currpage=1) | R [Arg] ⇒ Q [Gln] | 0.0002 |
| [rs201728690](https://www.ncbi.nlm.nih.gov/projects/SNP/snp_ref.cgi?rs=201728690) | CTC ⇒ CCC | [NP_473455.2](https://www.ncbi.nlm.nih.gov/entrez/query.fcgi?cmd=Search&db=protein&term=NP_473455.2) | [709](https://www.ncbi.nlm.nih.gov/sites/protein/NP_001265662.1?report=graph&v=596:696&content=5&m=646!&mn=rs201728690&dispmax=1&currpage=1) | L [Leu] ⇒ P [Pro] | 0.0002 |
| [rs367921611](https://www.ncbi.nlm.nih.gov/projects/SNP/snp_ref.cgi?rs=367921611) | CCC ⇒ GCC | [NP_473455.2](https://www.ncbi.nlm.nih.gov/entrez/query.fcgi?cmd=Search&db=protein&term=NP_473455.2) | [103](https://www.ncbi.nlm.nih.gov/sites/protein/NP_001265662.1?report=graph&v=1:90&content=5&m=40!&mn=rs367921611&dispmax=1&currpage=1) | P [Pro] ⇒ A [Ala] | 0.00008 |
| [rs368265576](https://www.ncbi.nlm.nih.gov/projects/SNP/snp_ref.cgi?rs=368265576) | ACG ⇒ ATG | [NP_473455.2](https://www.ncbi.nlm.nih.gov/entrez/query.fcgi?cmd=Search&db=protein&term=NP_473455.2) | [118](https://www.ncbi.nlm.nih.gov/sites/protein/NP_001265662.1?report=graph&v=5:105&content=5&m=55!&mn=rs368265576&dispmax=1&currpage=1) | T [Thr] ⇒ M [Met] | 0.00003 |
| [rs369913945](https://www.ncbi.nlm.nih.gov/projects/SNP/snp_ref.cgi?rs=369913945) | AGT ⇒ GGT | [NP_473455.2](https://www.ncbi.nlm.nih.gov/entrez/query.fcgi?cmd=Search&db=protein&term=NP_473455.2) | [479](https://www.ncbi.nlm.nih.gov/sites/protein/NP_001265662.1?report=graph&v=366:466&content=5&m=416!&mn=rs369913945&dispmax=1&currpage=1) | S [Ser] ⇒ G [Gly] | 0.0002 |
| [rs370044563](https://www.ncbi.nlm.nih.gov/projects/SNP/snp_ref.cgi?rs=370044563) | GAC ⇒ AAC | [NP_473455.2](https://www.ncbi.nlm.nih.gov/entrez/query.fcgi?cmd=Search&db=protein&term=NP_473455.2) | [257](https://www.ncbi.nlm.nih.gov/sites/protein/NP_001265662.1?report=graph&v=144:244&content=5&m=194!&mn=rs370044563&dispmax=1&currpage=1) | D [Asp] ⇒ N [Asn] | 0.002 |
| [rs371408856](https://www.ncbi.nlm.nih.gov/projects/SNP/snp_ref.cgi?rs=371408856) | CAG ⇒ CGG | [NP_473455.2](https://www.ncbi.nlm.nih.gov/entrez/query.fcgi?cmd=Search&db=protein&term=NP_473455.2) | [583](https://www.ncbi.nlm.nih.gov/sites/protein/NP_001265662.1?report=graph&v=470:570&content=5&m=520!&mn=rs371408856&dispmax=1&currpage=1) | Q [Gln] ⇒ R [Arg] | 0.0003 |
| [rs371800256](https://www.ncbi.nlm.nih.gov/projects/SNP/snp_ref.cgi?rs=371800256) | ATT ⇒ ACT | [NP_473455.2](https://www.ncbi.nlm.nih.gov/entrez/query.fcgi?cmd=Search&db=protein&term=NP_473455.2) | [92](https://www.ncbi.nlm.nih.gov/sites/protein/NP_001265662.1?report=graph&v=1:79&content=5&m=29!&mn=rs371800256&dispmax=1&currpage=1) | I [Ile] ⇒ T [Thr] | 0.00008 |
| [rs372170128](https://www.ncbi.nlm.nih.gov/projects/SNP/snp_ref.cgi?rs=372170128) | GTA ⇒ ATA | [NP_473455.2](https://www.ncbi.nlm.nih.gov/entrez/query.fcgi?cmd=Search&db=protein&term=NP_473455.2) | [643](https://www.ncbi.nlm.nih.gov/sites/protein/NP_001265662.1?report=graph&v=530:630&content=5&m=580!&mn=rs372170128&dispmax=1&currpage=1) | V [Val] ⇒ I [Ile] | 0.0001 |
| [rs373293958](https://www.ncbi.nlm.nih.gov/projects/SNP/snp_ref.cgi?rs=373293958) | GAA ⇒ AAA | [NP_473455.2](https://www.ncbi.nlm.nih.gov/entrez/query.fcgi?cmd=Search&db=protein&term=NP_473455.2) | [23](https://www.ncbi.nlm.nih.gov/sites/protein/NP_473455.2?report=graph&v=1:73&content=5&m=23!&mn=rs373293958&dispmax=1&currpage=1) | E [Glu] ⇒ K [Lys] | 0.00002 |
| [rs373518592](https://www.ncbi.nlm.nih.gov/projects/SNP/snp_ref.cgi?rs=373518592) | GAC ⇒ AAC | [NP_473455.2](https://www.ncbi.nlm.nih.gov/entrez/query.fcgi?cmd=Search&db=protein&term=NP_473455.2) | [173](https://www.ncbi.nlm.nih.gov/sites/protein/NP_001265662.1?report=graph&v=60:160&content=5&m=110!&mn=rs373518592&dispmax=1&currpage=1) | D [Asp] ⇒ N [Asn] | 0.00008 |
| [rs375463404](https://www.ncbi.nlm.nih.gov/projects/SNP/snp_ref.cgi?rs=375463404) | GCC ⇒ ACC | [NP_473455.2](https://www.ncbi.nlm.nih.gov/entrez/query.fcgi?cmd=Search&db=protein&term=NP_473455.2) | [15](https://www.ncbi.nlm.nih.gov/sites/protein/NP_473455.2?report=graph&v=1:65&content=5&m=15!&mn=rs375463404&dispmax=1&currpage=1) | A [Ala] ⇒ T [Thr] | 0.00008 |
| [rs375785212](https://www.ncbi.nlm.nih.gov/projects/SNP/snp_ref.cgi?rs=375785212) | AAA ⇒ AAT | [NP_473455.2](https://www.ncbi.nlm.nih.gov/entrez/query.fcgi?cmd=Search&db=protein&term=NP_473455.2) | [82](https://www.ncbi.nlm.nih.gov/sites/protein/NP_001265662.1?report=graph&v=1:69&content=5&m=19!&mn=rs375785212&dispmax=1&currpage=1) | K [Lys] ⇒ N [Asn] | 0.0001 |
| [rs376345765](https://www.ncbi.nlm.nih.gov/projects/SNP/snp_ref.cgi?rs=376345765) | CAC ⇒ TAC | [NP_473455.2](https://www.ncbi.nlm.nih.gov/entrez/query.fcgi?cmd=Search&db=protein&term=NP_473455.2) | [152](https://www.ncbi.nlm.nih.gov/sites/protein/NP_001265662.1?report=graph&v=39:139&content=5&m=89!&mn=rs376345765&dispmax=1&currpage=1) | H [His] ⇒ Y [Tyr] | 0.00008 |
| [rs376487437](https://www.ncbi.nlm.nih.gov/projects/SNP/snp_ref.cgi?rs=376487437) | GCG ⇒ GTG | [NP_473455.2](https://www.ncbi.nlm.nih.gov/entrez/query.fcgi?cmd=Search&db=protein&term=NP_473455.2) | [630](https://www.ncbi.nlm.nih.gov/sites/protein/NP_001265662.1?report=graph&v=517:617&content=5&m=567!&mn=rs376487437&dispmax=1&currpage=1) | A [Ala] ⇒ V [Val] | 0.00008 |
| [rs377118382](https://www.ncbi.nlm.nih.gov/projects/SNP/snp_ref.cgi?rs=377118382) | CAC ⇒ TAC | [NP_473455.2](https://www.ncbi.nlm.nih.gov/entrez/query.fcgi?cmd=Search&db=protein&term=NP_473455.2) | [648](https://www.ncbi.nlm.nih.gov/sites/protein/NP_001265662.1?report=graph&v=535:635&content=5&m=585!&mn=rs377118382&dispmax=1&currpage=1) | H [His] ⇒ Y [Tyr] | 0.00002 |
| [rs530252270](https://www.ncbi.nlm.nih.gov/projects/SNP/snp_ref.cgi?rs=530252270) | ACT ⇒ TCT | [NP_473455.2](https://www.ncbi.nlm.nih.gov/entrez/query.fcgi?cmd=Search&db=protein&term=NP_473455.2) | [672](https://www.ncbi.nlm.nih.gov/sites/protein/NP_001265662.1?report=graph&v=559:659&content=5&m=609!&mn=rs530252270&dispmax=1&currpage=1) | T [Thr] ⇒ S [Ser] | 0.0002 |
| [rs530412401](https://www.ncbi.nlm.nih.gov/projects/SNP/snp_ref.cgi?rs=530412401) | GTG ⇒ GAG | [NP_473455.2](https://www.ncbi.nlm.nih.gov/entrez/query.fcgi?cmd=Search&db=protein&term=NP_473455.2) | [608](https://www.ncbi.nlm.nih.gov/sites/protein/NP_001265662.1?report=graph&v=495:595&content=5&m=545!&mn=rs530412401&dispmax=1&currpage=1) | V [Val] ⇒ E [Glu] | 0.000008 |
| [rs534119067](https://www.ncbi.nlm.nih.gov/projects/SNP/snp_ref.cgi?rs=534119067) | AAT ⇒ AGT | [NP_473455.2](https://www.ncbi.nlm.nih.gov/entrez/query.fcgi?cmd=Search&db=protein&term=NP_473455.2) | [8](https://www.ncbi.nlm.nih.gov/sites/protein/NP_473455.2?report=graph&v=1:58&content=5&m=8!&mn=rs534119067&dispmax=1&currpage=1) | N [Asn] ⇒ S [Ser] | 0.0002 |
| [rs537442916](https://www.ncbi.nlm.nih.gov/projects/SNP/snp_ref.cgi?rs=537442916) | TAT ⇒ CAT | [NP_473455.2](https://www.ncbi.nlm.nih.gov/entrez/query.fcgi?cmd=Search&db=protein&term=NP_473455.2) | [725](https://www.ncbi.nlm.nih.gov/sites/protein/NP_001265662.1?report=graph&v=612:712&content=5&m=662!&mn=rs537442916&dispmax=1&currpage=1) | Y [Tyr] ⇒ H [His] | 0.00003 |
| [rs539913868](https://www.ncbi.nlm.nih.gov/projects/SNP/snp_ref.cgi?rs=539913868) | ATT ⇒ ACT | [NP_473455.2](https://www.ncbi.nlm.nih.gov/entrez/query.fcgi?cmd=Search&db=protein&term=NP_473455.2) | [270](https://www.ncbi.nlm.nih.gov/sites/protein/NP_001265662.1?report=graph&v=157:257&content=5&m=207!&mn=rs539913868&dispmax=1&currpage=1) | I [Ile] ⇒ T [Thr] | 0.0002 |
| [rs544406093](https://www.ncbi.nlm.nih.gov/projects/SNP/snp_ref.cgi?rs=544406093) | AGC ⇒ AGA | [NP_473455.2](https://www.ncbi.nlm.nih.gov/entrez/query.fcgi?cmd=Search&db=protein&term=NP_473455.2) | [5](https://www.ncbi.nlm.nih.gov/sites/protein/NP_473455.2?report=graph&v=1:55&content=5&m=5!&mn=rs544406093&dispmax=1&currpage=1) | S [Ser] ⇒ R [Arg] | 0.00007 |
| [rs553216931](https://www.ncbi.nlm.nih.gov/projects/SNP/snp_ref.cgi?rs=553216931) | AAC ⇒ AGC | [NP_473455.2](https://www.ncbi.nlm.nih.gov/entrez/query.fcgi?cmd=Search&db=protein&term=NP_473455.2) | [563](https://www.ncbi.nlm.nih.gov/sites/protein/NP_001265662.1?report=graph&v=450:550&content=5&m=500!&mn=rs553216931&dispmax=1&currpage=1) | N [Asn] ⇒ S [Ser] | 0.0002 |
| [rs553431311](https://www.ncbi.nlm.nih.gov/projects/SNP/snp_ref.cgi?rs=553431311) | CGT ⇒ TGT | [NP_473455.2](https://www.ncbi.nlm.nih.gov/entrez/query.fcgi?cmd=Search&db=protein&term=NP_473455.2) | [131](https://www.ncbi.nlm.nih.gov/sites/protein/NP_001265662.1?report=graph&v=18:118&content=5&m=68!&mn=rs553431311&dispmax=1&currpage=1) | R [Arg] ⇒ C [Cys] | 0.0002 |
| [rs555114313](https://www.ncbi.nlm.nih.gov/projects/SNP/snp_ref.cgi?rs=555114313) | TCT ⇒ TGT | [NP_473455.2](https://www.ncbi.nlm.nih.gov/entrez/query.fcgi?cmd=Search&db=protein&term=NP_473455.2) | [469](https://www.ncbi.nlm.nih.gov/sites/protein/NP_001265662.1?report=graph&v=356:456&content=5&m=406!&mn=rs555114313&dispmax=1&currpage=1) | S [Ser] ⇒ C [Cys] | 0.0002 |
| [rs556216423](https://www.ncbi.nlm.nih.gov/projects/SNP/snp_ref.cgi?rs=556216423) | GGA ⇒ AGA | [NP_473455.2](https://www.ncbi.nlm.nih.gov/entrez/query.fcgi?cmd=Search&db=protein&term=NP_473455.2) | [659](https://www.ncbi.nlm.nih.gov/sites/protein/NP_001265662.1?report=graph&v=546:646&content=5&m=596!&mn=rs556216423&dispmax=1&currpage=1) | G [Gly] ⇒ R [Arg] | 0.000008 |
| [rs560226623](https://www.ncbi.nlm.nih.gov/projects/SNP/snp_ref.cgi?rs=560226623) | GTG ⇒ ATG | [NP_473455.2](https://www.ncbi.nlm.nih.gov/entrez/query.fcgi?cmd=Search&db=protein&term=NP_473455.2) | [266](https://www.ncbi.nlm.nih.gov/sites/protein/NP_001265662.1?report=graph&v=153:253&content=5&m=203!&mn=rs560226623&dispmax=1&currpage=1) | V [Val] ⇒ M [Met] | 0.002 |
| [rs560653809](https://www.ncbi.nlm.nih.gov/projects/SNP/snp_ref.cgi?rs=560653809) | CCT ⇒ CAT | [NP_473455.2](https://www.ncbi.nlm.nih.gov/entrez/query.fcgi?cmd=Search&db=protein&term=NP_473455.2) | [636](https://www.ncbi.nlm.nih.gov/sites/protein/NP_001265662.1?report=graph&v=523:623&content=5&m=573!&mn=rs560653809&dispmax=1&currpage=1) | P [Pro] ⇒ H [His] | 0.0004 |
| [rs562716751](https://www.ncbi.nlm.nih.gov/projects/SNP/snp_ref.cgi?rs=562716751) | CGA ⇒ CAA | [NP_473455.2](https://www.ncbi.nlm.nih.gov/entrez/query.fcgi?cmd=Search&db=protein&term=NP_001265662.1) | [351](https://www.ncbi.nlm.nih.gov/sites/protein/NP_001265662.1?report=graph&v=238:338&content=5&m=288!&mn=rs562716751&dispmax=1&currpage=1) | R [Arg] ⇒ Q [Gln] | 0.000008 |
| [rs567097355](https://www.ncbi.nlm.nih.gov/projects/SNP/snp_ref.cgi?rs=567097355) | CAG ⇒ AAG | [NP_473455.2](https://www.ncbi.nlm.nih.gov/entrez/query.fcgi?cmd=Search&db=protein&term=NP_001265662.1) | [583](https://www.ncbi.nlm.nih.gov/sites/protein/NP_001265662.1?report=graph&v=470:570&content=5&m=520!&mn=rs567097355&dispmax=1&currpage=1) | Q [Gln] ⇒ K [Lys] | 0.0004 |
| [rs568588813](https://www.ncbi.nlm.nih.gov/projects/SNP/snp_ref.cgi?rs=568588813) | GCT ⇒ GGT | [NP_473455.2](https://www.ncbi.nlm.nih.gov/entrez/query.fcgi?cmd=Search&db=protein&term=NP_473455.2) | [9](https://www.ncbi.nlm.nih.gov/sites/protein/NP_473455.2?report=graph&v=1:59&content=5&m=9!&mn=rs568588813&dispmax=1&currpage=1) | A [Ala] ⇒ G [Gly] | 0.0002 |
| [rs569120071](https://www.ncbi.nlm.nih.gov/projects/SNP/snp_ref.cgi?rs=569120071) | ATG ⇒ GTG | [NP_473455.2](https://www.ncbi.nlm.nih.gov/entrez/query.fcgi?cmd=Search&db=protein&term=NP_473455.2) | [18](https://www.ncbi.nlm.nih.gov/sites/protein/NP_473455.2?report=graph&v=1:68&content=5&m=18!&mn=rs569120071&dispmax=1&currpage=1) | M [Met] ⇒ V [Val] | 0.0002 |
| [rs569240044](https://www.ncbi.nlm.nih.gov/projects/SNP/snp_ref.cgi?rs=569240044) | CCT ⇒ CTT | [NP_473455.2](https://www.ncbi.nlm.nih.gov/entrez/query.fcgi?cmd=Search&db=protein&term=NP_473455.2) | [315](https://www.ncbi.nlm.nih.gov/sites/protein/NP_001265662.1?report=graph&v=202:302&content=5&m=252!&mn=rs569240044&dispmax=1&currpage=1) | P [Pro] ⇒ L [Leu] | 0.0002 |
| [rs573417625](https://www.ncbi.nlm.nih.gov/projects/SNP/snp_ref.cgi?rs=573417625) | AAA ⇒ GAA | [NP_473455.2](https://www.ncbi.nlm.nih.gov/entrez/query.fcgi?cmd=Search&db=protein&term=NP_473455.2) | [114](https://www.ncbi.nlm.nih.gov/sites/protein/NP_001265662.1?report=graph&v=1:101&content=5&m=51!&mn=rs573417625&dispmax=1&currpage=1) | K [Lys] ⇒ E [Glu] | 0.0002 |
| [rs574958409](https://www.ncbi.nlm.nih.gov/projects/SNP/snp_ref.cgi?rs=574958409) | TCG ⇒ TTG | [NP_473455.2](https://www.ncbi.nlm.nih.gov/entrez/query.fcgi?cmd=Search&db=protein&term=NP_473455.2) | [464](https://www.ncbi.nlm.nih.gov/sites/protein/NP_001265662.1?report=graph&v=351:451&content=5&m=401!&mn=rs574958409&dispmax=1&currpage=1) | S [Ser] ⇒ L [Leu] | 0.0002 |
| [rs575712277](https://www.ncbi.nlm.nih.gov/projects/SNP/snp_ref.cgi?rs=575712277) | CCC ⇒ TCC | [NP_473455.2](https://www.ncbi.nlm.nih.gov/entrez/query.fcgi?cmd=Search&db=protein&term=NP_473455.2) | [480](https://www.ncbi.nlm.nih.gov/sites/protein/NP_001265662.1?report=graph&v=367:467&content=5&m=417!&mn=rs575712277&dispmax=1&currpage=1) | P [Pro] ⇒ S [Ser] | 0.0002 |
| [rs577701787](https://www.ncbi.nlm.nih.gov/projects/SNP/snp_ref.cgi?rs=577701787) | GAC ⇒ GGC | [NP_473455.2](https://www.ncbi.nlm.nih.gov/entrez/query.fcgi?cmd=Search&db=protein&term=NP_473455.2) | [297](https://www.ncbi.nlm.nih.gov/sites/protein/NP_001265662.1?report=graph&v=184:284&content=5&m=234!&mn=rs577701787&dispmax=1&currpage=1) | D [Asp] ⇒ G [Gly] | 0.001 |
| [rs745508567](https://www.ncbi.nlm.nih.gov/projects/SNP/snp_ref.cgi?rs=745508567) | CTG ⇒ ATG | [NP_473455.2](https://www.ncbi.nlm.nih.gov/entrez/query.fcgi?cmd=Search&db=protein&term=NP_473455.2) | [194](https://www.ncbi.nlm.nih.gov/sites/protein/NP_001265662.1?report=graph&v=81:181&content=5&m=131!&mn=rs745508567&dispmax=1&currpage=1) | L [Leu] ⇒ M [Met] | 0.00004 |
| [rs745819016](https://www.ncbi.nlm.nih.gov/projects/SNP/snp_ref.cgi?rs=745819016) | AAA ⇒ ACA | [NP_473455.2](https://www.ncbi.nlm.nih.gov/entrez/query.fcgi?cmd=Search&db=protein&term=NP_473455.2) | [668](https://www.ncbi.nlm.nih.gov/sites/protein/NP_001265662.1?report=graph&v=555:655&content=5&m=605!&mn=rs745819016&dispmax=1&currpage=1) | K [Lys] ⇒ T [Thr] | 0.000008 |
| [rs746408693](https://www.ncbi.nlm.nih.gov/projects/SNP/snp_ref.cgi?rs=746408693) | TCG ⇒ TTG | [NP_473455.2](https://www.ncbi.nlm.nih.gov/entrez/query.fcgi?cmd=Search&db=protein&term=NP_473455.2) | [577](https://www.ncbi.nlm.nih.gov/sites/protein/NP_001265662.1?report=graph&v=464:564&content=5&m=514!&mn=rs746408693&dispmax=1&currpage=1) | S [Ser] ⇒ L [Leu] | 0.000008 |
| [rs746865393](https://www.ncbi.nlm.nih.gov/projects/SNP/snp_ref.cgi?rs=746865393) | GCG ⇒ GTG | [NP_473455.2](https://www.ncbi.nlm.nih.gov/entrez/query.fcgi?cmd=Search&db=protein&term=NP_473455.2) | [143](https://www.ncbi.nlm.nih.gov/sites/protein/NP_001265662.1?report=graph&v=30:130&content=5&m=80!&mn=rs746865393&dispmax=1&currpage=1) | A [Ala] ⇒ V [Val] | 0.000008 |
| [rs746948813](https://www.ncbi.nlm.nih.gov/projects/SNP/snp_ref.cgi?rs=746948813) | GGC ⇒ AGC | [NP_473455.2](https://www.ncbi.nlm.nih.gov/entrez/query.fcgi?cmd=Search&db=protein&term=NP_473455.2) | [346](https://www.ncbi.nlm.nih.gov/sites/protein/NP_001265662.1?report=graph&v=233:333&content=5&m=283!&mn=rs746948813&dispmax=1&currpage=1) | G [Gly] ⇒ S [Ser] | 0.000008 |
| [rs747203170](https://www.ncbi.nlm.nih.gov/projects/SNP/snp_ref.cgi?rs=747203170) | TTC ⇒ CTC | [NP_473455.2](https://www.ncbi.nlm.nih.gov/entrez/query.fcgi?cmd=Search&db=protein&term=NP_473455.2) | [582](https://www.ncbi.nlm.nih.gov/sites/protein/NP_001265662.1?report=graph&v=469:569&content=5&m=519!&mn=rs747203170&dispmax=1&currpage=1) | F [Phe] ⇒ L [Leu] | 0.000008 |
| [rs747252106](https://www.ncbi.nlm.nih.gov/projects/SNP/snp_ref.cgi?rs=747252106) | GAC ⇒ AAC | [NP_473455.2](https://www.ncbi.nlm.nih.gov/entrez/query.fcgi?cmd=Search&db=protein&term=NP_473455.2) | [537](https://www.ncbi.nlm.nih.gov/sites/protein/NP_001265662.1?report=graph&v=424:524&content=5&m=474!&mn=rs747252106&dispmax=1&currpage=1) | D [Asp] ⇒ N [Asn] | 0.000008 |
| [rs747602006](https://www.ncbi.nlm.nih.gov/projects/SNP/snp_ref.cgi?rs=747602006) | GAT ⇒ AAT | [NP_473455.2](https://www.ncbi.nlm.nih.gov/entrez/query.fcgi?cmd=Search&db=protein&term=NP_473455.2) | [373](https://www.ncbi.nlm.nih.gov/sites/protein/NP_001265662.1?report=graph&v=260:360&content=5&m=310!&mn=rs747602006&dispmax=1&currpage=1) | D [Asp] ⇒ N [Asn] | 0.00002 |
| [rs747793651](https://www.ncbi.nlm.nih.gov/projects/SNP/snp_ref.cgi?rs=747793651) | CCG ⇒ CAG | [NP_473455.2](https://www.ncbi.nlm.nih.gov/entrez/query.fcgi?cmd=Search&db=protein&term=NP_473455.2) | [716](https://www.ncbi.nlm.nih.gov/sites/protein/NP_001265662.1?report=graph&v=603:703&content=5&m=653!&mn=rs747793651&dispmax=1&currpage=1) | P [Pro] ⇒ Q [Gln] | 0.000008 |
| [rs747847015](https://www.ncbi.nlm.nih.gov/projects/SNP/snp_ref.cgi?rs=747847015) | CAG ⇒ CGG | [NP_473455.2](https://www.ncbi.nlm.nih.gov/entrez/query.fcgi?cmd=Search&db=protein&term=NP_473455.2) | [702](https://www.ncbi.nlm.nih.gov/sites/protein/NP_001265662.1?report=graph&v=589:689&content=5&m=639!&mn=rs747847015&dispmax=1&currpage=1) | Q [Gln] ⇒ R [Arg] | 0.00003 |
| [rs748068691](https://www.ncbi.nlm.nih.gov/projects/SNP/snp_ref.cgi?rs=748068691) | AGG ⇒ AGT | [NP_473455.2](https://www.ncbi.nlm.nih.gov/entrez/query.fcgi?cmd=Search&db=protein&term=NP_473455.2) | [148](https://www.ncbi.nlm.nih.gov/sites/protein/NP_001265662.1?report=graph&v=35:135&content=5&m=85!&mn=rs748068691&dispmax=1&currpage=1) | R [Arg] ⇒ S [Ser] | 0.000008 |
| [rs748117200](https://www.ncbi.nlm.nih.gov/projects/SNP/snp_ref.cgi?rs=748117200) | CGG ⇒ TGG | [NP_473455.2](https://www.ncbi.nlm.nih.gov/entrez/query.fcgi?cmd=Search&db=protein&term=NP_473455.2) | [408](https://www.ncbi.nlm.nih.gov/sites/protein/NP_001265662.1?report=graph&v=295:395&content=5&m=345!&mn=rs748117200&dispmax=1&currpage=1) | R [Arg] ⇒ W [Trp] | 0.00002 |
| [rs748319319](https://www.ncbi.nlm.nih.gov/projects/SNP/snp_ref.cgi?rs=748319319) | GAC ⇒ AAC | [NP_473455.2](https://www.ncbi.nlm.nih.gov/entrez/query.fcgi?cmd=Search&db=protein&term=NP_473455.2) | [314](https://www.ncbi.nlm.nih.gov/sites/protein/NP_001265662.1?report=graph&v=201:301&content=5&m=251!&mn=rs748319319&dispmax=1&currpage=1) | D [Asp] ⇒ N [Asn] | 0.000008 |
| [rs748465896](https://www.ncbi.nlm.nih.gov/projects/SNP/snp_ref.cgi?rs=748465896) | CAT ⇒ TAT | [NP_473455.2](https://www.ncbi.nlm.nih.gov/entrez/query.fcgi?cmd=Search&db=protein&term=NP_473455.2) | [488](https://www.ncbi.nlm.nih.gov/sites/protein/NP_001265662.1?report=graph&v=375:475&content=5&m=425!&mn=rs748465896&dispmax=1&currpage=1) | H [His] ⇒ Y [Tyr] | 0.00002 |
| [rs748633493](https://www.ncbi.nlm.nih.gov/projects/SNP/snp_ref.cgi?rs=748633493) | CGG ⇒ CAG | [NP_473455.2](https://www.ncbi.nlm.nih.gov/entrez/query.fcgi?cmd=Search&db=protein&term=NP_473455.2) | [453](https://www.ncbi.nlm.nih.gov/sites/protein/NP_001265662.1?report=graph&v=340:440&content=5&m=390!&mn=rs748633493&dispmax=1&currpage=1) | R [Arg] ⇒ Q [Gln] | 0.00003 |
| [rs748659041](https://www.ncbi.nlm.nih.gov/projects/SNP/snp_ref.cgi?rs=748659041) | CTC ⇒ TTC | [NP_473455.2](https://www.ncbi.nlm.nih.gov/entrez/query.fcgi?cmd=Search&db=protein&term=NP_473455.2) | [100](https://www.ncbi.nlm.nih.gov/sites/protein/NP_001265662.1?report=graph&v=1:87&content=5&m=37!&mn=rs748659041&dispmax=1&currpage=1) | L [Leu] ⇒ F [Phe] | 0.00002 |
| [rs748963742](https://www.ncbi.nlm.nih.gov/projects/SNP/snp_ref.cgi?rs=748963742) | TAC ⇒ CAC | [NP_473455.2](https://www.ncbi.nlm.nih.gov/entrez/query.fcgi?cmd=Search&db=protein&term=NP_473455.2) | [376](https://www.ncbi.nlm.nih.gov/sites/protein/NP_001265662.1?report=graph&v=263:363&content=5&m=313!&mn=rs748963742&dispmax=1&currpage=1) | Y [Tyr] ⇒ H [His] | 0.00002 |
| [rs749729744](https://www.ncbi.nlm.nih.gov/projects/SNP/snp_ref.cgi?rs=749729744) | GCG ⇒ GGG | [NP_473455.2](https://www.ncbi.nlm.nih.gov/entrez/query.fcgi?cmd=Search&db=protein&term=NP_473455.2) | [526](https://www.ncbi.nlm.nih.gov/sites/protein/NP_001265662.1?report=graph&v=413:513&content=5&m=463!&mn=rs749729744&dispmax=1&currpage=1) | A [Ala] ⇒ G [Gly] | 0.000008 |
| [rs749826063](https://www.ncbi.nlm.nih.gov/projects/SNP/snp_ref.cgi?rs=749826063) | TTC ⇒ TGC | [NP_473455.2](https://www.ncbi.nlm.nih.gov/entrez/query.fcgi?cmd=Search&db=protein&term=NP_473455.2) | [491](https://www.ncbi.nlm.nih.gov/sites/protein/NP_001265662.1?report=graph&v=378:478&content=5&m=428!&mn=rs749826063&dispmax=1&currpage=1) | F [Phe] ⇒ C [Cys] | 0.000008 |
| [rs750084508](https://www.ncbi.nlm.nih.gov/projects/SNP/snp_ref.cgi?rs=750084508) | GGC ⇒ TGC | [NP_473455.2](https://www.ncbi.nlm.nih.gov/entrez/query.fcgi?cmd=Search&db=protein&term=NP_473455.2) | [602](https://www.ncbi.nlm.nih.gov/sites/protein/NP_001265662.1?report=graph&v=489:589&content=5&m=539!&mn=rs750084508&dispmax=1&currpage=1) | G [Gly] ⇒ C [Cys] | 0.00002 |
| [rs750610271](https://www.ncbi.nlm.nih.gov/projects/SNP/snp_ref.cgi?rs=750610271) | GGC ⇒ GAC | [NP_473455.2](https://www.ncbi.nlm.nih.gov/entrez/query.fcgi?cmd=Search&db=protein&term=NP_473455.2) | [498](https://www.ncbi.nlm.nih.gov/sites/protein/NP_001265662.1?report=graph&v=385:485&content=5&m=435!&mn=rs750610271&dispmax=1&currpage=1) | G [Gly] ⇒ D [Asp] | 0.00002 |
| [rs751021592](https://www.ncbi.nlm.nih.gov/projects/SNP/snp_ref.cgi?rs=751021592) | CAG ⇒ CTG | [NP_473455.2](https://www.ncbi.nlm.nih.gov/entrez/query.fcgi?cmd=Search&db=protein&term=NP_473455.2) | [333](https://www.ncbi.nlm.nih.gov/sites/protein/NP_001265662.1?report=graph&v=220:320&content=5&m=270!&mn=rs751021592&dispmax=1&currpage=1) | Q [Gln] ⇒ L [Leu] | 0.000008 |
| [rs751052061](https://www.ncbi.nlm.nih.gov/projects/SNP/snp_ref.cgi?rs=751052061) | TCG ⇒ ACG | [NP_473455.2](https://www.ncbi.nlm.nih.gov/entrez/query.fcgi?cmd=Search&db=protein&term=NP_001265662.1) | [84](https://www.ncbi.nlm.nih.gov/sites/protein/NP_001265662.1?report=graph&v=1:71&content=5&m=21!&mn=rs751052061&dispmax=1&currpage=1) | S [Ser] ⇒ T [Thr] | 0.000008 |
| [rs751285505](https://www.ncbi.nlm.nih.gov/projects/SNP/snp_ref.cgi?rs=751285505) | AGC ⇒ AGG | [NP_473455.2](https://www.ncbi.nlm.nih.gov/entrez/query.fcgi?cmd=Search&db=protein&term=NP_473455.2) | [501](https://www.ncbi.nlm.nih.gov/sites/protein/NP_001265662.1?report=graph&v=388:488&content=5&m=438!&mn=rs751285505&dispmax=1&currpage=1) | S [Ser] ⇒ R [Arg] | 0.00002 |
| [rs751645528](https://www.ncbi.nlm.nih.gov/projects/SNP/snp_ref.cgi?rs=751645528) | GTG ⇒ ATG | [NP_473455.2](https://www.ncbi.nlm.nih.gov/entrez/query.fcgi?cmd=Search&db=protein&term=NP_473455.2) | [710](https://www.ncbi.nlm.nih.gov/sites/protein/NP_001265662.1?report=graph&v=597:697&content=5&m=647!&mn=rs751645528&dispmax=1&currpage=1) | V [Val] ⇒ M [Met] | 0.000008 |
| [rs751773024](https://www.ncbi.nlm.nih.gov/projects/SNP/snp_ref.cgi?rs=751773024) | TCA ⇒ CCA | [NP_473455.2](https://www.ncbi.nlm.nih.gov/entrez/query.fcgi?cmd=Search&db=protein&term=NP_473455.2) | [10](https://www.ncbi.nlm.nih.gov/sites/protein/NP_473455.2?report=graph&v=1:60&content=5&m=10!&mn=rs751773024&dispmax=1&currpage=1) | S [Ser] ⇒ P [Pro] | 0.000008 |
| [rs751800889](https://www.ncbi.nlm.nih.gov/projects/SNP/snp_ref.cgi?rs=751800889) | CGG ⇒ TGG | [NP_473455.2](https://www.ncbi.nlm.nih.gov/entrez/query.fcgi?cmd=Search&db=protein&term=NP_473455.2) | [203](https://www.ncbi.nlm.nih.gov/sites/protein/NP_001265662.1?report=graph&v=90:190&content=5&m=140!&mn=rs751800889&dispmax=1&currpage=1) | R [Arg] ⇒ W [Trp] | 0.000008 |
| [rs751891541](https://www.ncbi.nlm.nih.gov/projects/SNP/snp_ref.cgi?rs=751891541) | GTC ⇒ ATC | [NP_473455.2](https://www.ncbi.nlm.nih.gov/entrez/query.fcgi?cmd=Search&db=protein&term=NP_473455.2) | [157](https://www.ncbi.nlm.nih.gov/sites/protein/NP_001265662.1?report=graph&v=44:144&content=5&m=94!&mn=rs751891541&dispmax=1&currpage=1) | V [Val] ⇒ I [Ile] | 0.00002 |
| [rs752195559](https://www.ncbi.nlm.nih.gov/projects/SNP/snp_ref.cgi?rs=752195559) | ATT ⇒ GTT | [NP_473455.2](https://www.ncbi.nlm.nih.gov/entrez/query.fcgi?cmd=Search&db=protein&term=NP_473455.2) | [110](https://www.ncbi.nlm.nih.gov/sites/protein/NP_001265662.1?report=graph&v=1:97&content=5&m=47!&mn=rs752195559&dispmax=1&currpage=1) | I [Ile] ⇒ V [Val] | 0.000008 |
| [rs752343825](https://www.ncbi.nlm.nih.gov/projects/SNP/snp_ref.cgi?rs=752343825) | GTG ⇒ ATG | [NP_473455.2](https://www.ncbi.nlm.nih.gov/entrez/query.fcgi?cmd=Search&db=protein&term=NP_473455.2) | [450](https://www.ncbi.nlm.nih.gov/sites/protein/NP_001265662.1?report=graph&v=337:437&content=5&m=387!&mn=rs752343825&dispmax=1&currpage=1) | V [Val] ⇒ M [Met] | 0.00005 |
| [rs753717176](https://www.ncbi.nlm.nih.gov/projects/SNP/snp_ref.cgi?rs=753717176) | TTC ⇒ TTA | [NP_473455.2](https://www.ncbi.nlm.nih.gov/entrez/query.fcgi?cmd=Search&db=protein&term=NP_473455.2) | [268](https://www.ncbi.nlm.nih.gov/sites/protein/NP_001265662.1?report=graph&v=155:255&content=5&m=205!&mn=rs753717176&dispmax=1&currpage=1) | F [Phe] ⇒ L [Leu] | 0.000008 |
| [rs753873808](https://www.ncbi.nlm.nih.gov/projects/SNP/snp_ref.cgi?rs=753873808) | GAA ⇒ AAA | [NP_473455.2](https://www.ncbi.nlm.nih.gov/entrez/query.fcgi?cmd=Search&db=protein&term=NP_473455.2) | [597](https://www.ncbi.nlm.nih.gov/sites/protein/NP_001265662.1?report=graph&v=484:584&content=5&m=534!&mn=rs753873808&dispmax=1&currpage=1) | E [Glu] ⇒ K [Lys] | 0.000008 |
| [rs753904397](https://www.ncbi.nlm.nih.gov/projects/SNP/snp_ref.cgi?rs=753904397) | CGG ⇒ TGG | [NP_473455.2](https://www.ncbi.nlm.nih.gov/entrez/query.fcgi?cmd=Search&db=protein&term=NP_473455.2) | [167](https://www.ncbi.nlm.nih.gov/sites/protein/NP_001265662.1?report=graph&v=54:154&content=5&m=104!&mn=rs753904397&dispmax=1&currpage=1) | R [Arg] ⇒ W [Trp] | 0.00004 |
| rs754331104 | CAC ⇒ TAC | [NP_473455.2](https://www.ncbi.nlm.nih.gov/entrez/query.fcgi?cmd=Search&db=protein&term=NP_473455.2) | [642](https://www.ncbi.nlm.nih.gov/sites/protein/NP_001265662.1?report=graph&v=529:629&content=5&m=579!&mn=rs754331104&dispmax=1&currpage=1) | H [His] ⇒ Y [Tyr] | 0.00002 |
| [rs754875937](https://www.ncbi.nlm.nih.gov/projects/SNP/snp_ref.cgi?rs=754875937) | CGA ⇒ CAA | [NP_473455.2](https://www.ncbi.nlm.nih.gov/entrez/query.fcgi?cmd=Search&db=protein&term=NP_473455.2) | [552](https://www.ncbi.nlm.nih.gov/sites/protein/NP_001265662.1?report=graph&v=439:539&content=5&m=489!&mn=rs754875937&dispmax=1&currpage=1) | R [Arg] ⇒ Q [Gln] | 0.000008 |
| [rs755089373](https://www.ncbi.nlm.nih.gov/projects/SNP/snp_ref.cgi?rs=755089373) | CCT ⇒ GCT | [NP_473455.2](https://www.ncbi.nlm.nih.gov/entrez/query.fcgi?cmd=Search&db=protein&term=NP_001265662.1) | [69](https://www.ncbi.nlm.nih.gov/sites/protein/NP_001265662.1?report=graph&v=1:56&content=5&m=6!&mn=rs755089373&dispmax=1&currpage=1) | P [Pro] ⇒ A [Ala] | 0.00003 |
| [rs755149090](https://www.ncbi.nlm.nih.gov/projects/SNP/snp_ref.cgi?rs=755149090) | CCA ⇒ ACA | [NP_473455.2](https://www.ncbi.nlm.nih.gov/entrez/query.fcgi?cmd=Search&db=protein&term=NP_473455.2) | [421](https://www.ncbi.nlm.nih.gov/sites/protein/NP_001265662.1?report=graph&v=308:408&content=5&m=358!&mn=rs755149090&dispmax=1&currpage=1) | P [Pro] ⇒ T [Thr] | 0.000008 |
| [rs755749973](https://www.ncbi.nlm.nih.gov/projects/SNP/snp_ref.cgi?rs=755749973) | GCC ⇒ ACC | [NP_473455.2](https://www.ncbi.nlm.nih.gov/entrez/query.fcgi?cmd=Search&db=protein&term=NP_473455.2) | [599](https://www.ncbi.nlm.nih.gov/sites/protein/NP_001265662.1?report=graph&v=486:586&content=5&m=536!&mn=rs755749973&dispmax=1&currpage=1) | A [Ala] ⇒ T [Thr] | 0.000008 |
| [rs756130035](https://www.ncbi.nlm.nih.gov/projects/SNP/snp_ref.cgi?rs=756130035) | CAC ⇒ GAC | [NP_473455.2](https://www.ncbi.nlm.nih.gov/entrez/query.fcgi?cmd=Search&db=protein&term=NP_473455.2) | [631](https://www.ncbi.nlm.nih.gov/sites/protein/NP_001265662.1?report=graph&v=518:618&content=5&m=568!&mn=rs756130035&dispmax=1&currpage=1) | H [His] ⇒ D [Asp] | 0.000008 |
| [rs756500896](https://www.ncbi.nlm.nih.gov/projects/SNP/snp_ref.cgi?rs=756500896) | CGG ⇒ TGG | [NP_473455.2](https://www.ncbi.nlm.nih.gov/entrez/query.fcgi?cmd=Search&db=protein&term=NP_473455.2) | [706](https://www.ncbi.nlm.nih.gov/sites/protein/NP_001265662.1?report=graph&v=593:693&content=5&m=643!&mn=rs756500896&dispmax=1&currpage=1) | R [Arg] ⇒ W [Trp] | 0.000008 |
| [rs756704870](https://www.ncbi.nlm.nih.gov/projects/SNP/snp_ref.cgi?rs=756704870) | CCC ⇒ TCC | [NP_473455.2](https://www.ncbi.nlm.nih.gov/entrez/query.fcgi?cmd=Search&db=protein&term=NP_473455.2) | [332](https://www.ncbi.nlm.nih.gov/sites/protein/NP_001265662.1?report=graph&v=219:319&content=5&m=269!&mn=rs756704870&dispmax=1&currpage=1) | P [Pro] ⇒ S [Ser] | 0.000008 |
| [rs756759950](https://www.ncbi.nlm.nih.gov/projects/SNP/snp_ref.cgi?rs=756759950) | AGC ⇒ AAC | [NP_473455.2](https://www.ncbi.nlm.nih.gov/entrez/query.fcgi?cmd=Search&db=protein&term=NP_473455.2) | [670](https://www.ncbi.nlm.nih.gov/sites/protein/NP_001265662.1?report=graph&v=557:657&content=5&m=607!&mn=rs756759950&dispmax=1&currpage=1) | S [Ser] ⇒ N [Asn] | 0.0001 |
| [rs757784048](https://www.ncbi.nlm.nih.gov/projects/SNP/snp_ref.cgi?rs=757784048) | TGT ⇒ CGT | [NP_473455.2](https://www.ncbi.nlm.nih.gov/entrez/query.fcgi?cmd=Search&db=protein&term=NP_473455.2) | [708](https://www.ncbi.nlm.nih.gov/sites/protein/NP_001265662.1?report=graph&v=595:695&content=5&m=645!&mn=rs757784048&dispmax=1&currpage=1) | C [Cys] ⇒ R [Arg] | 0.000008 |
| [rs758007058](https://www.ncbi.nlm.nih.gov/projects/SNP/snp_ref.cgi?rs=758007058) | GTG ⇒ TTG | [NP_473455.2](https://www.ncbi.nlm.nih.gov/entrez/query.fcgi?cmd=Search&db=protein&term=NP_473455.2) | [701](https://www.ncbi.nlm.nih.gov/sites/protein/NP_001265662.1?report=graph&v=588:688&content=5&m=638!&mn=rs758007058&dispmax=1&currpage=1) | V [Val] ⇒ L [Leu] | 0.000008 |
| [rs758029090](https://www.ncbi.nlm.nih.gov/projects/SNP/snp_ref.cgi?rs=758029090) | CCC ⇒ CTC | [NP_473455.2](https://www.ncbi.nlm.nih.gov/entrez/query.fcgi?cmd=Search&db=protein&term=NP_473455.2) | [335](https://www.ncbi.nlm.nih.gov/sites/protein/NP_001265662.1?report=graph&v=222:322&content=5&m=272!&mn=rs758029090&dispmax=1&currpage=1) | P [Pro] ⇒ L [Leu] | 0.000008 |
| [rs758060464](https://www.ncbi.nlm.nih.gov/projects/SNP/snp_ref.cgi?rs=758060464) | ACT ⇒ ATT | [NP_473455.2](https://www.ncbi.nlm.nih.gov/entrez/query.fcgi?cmd=Search&db=protein&term=NP_473455.2) | [672](https://www.ncbi.nlm.nih.gov/sites/protein/NP_001265662.1?report=graph&v=559:659&content=5&m=609%21&mn=rs758060464&dispmax=1&currpage=1) | T [Thr] ⇒ I [Ile] | 0.00003 |
| [rs758251988](https://www.ncbi.nlm.nih.gov/projects/SNP/snp_ref.cgi?rs=758251988) | AGG ⇒ AGC | [NP_473455.2](https://www.ncbi.nlm.nih.gov/entrez/query.fcgi?cmd=Search&db=protein&term=NP_473455.2) | [541](https://www.ncbi.nlm.nih.gov/sites/protein/NP_001265662.1?report=graph&v=428:528&content=5&m=478%21&mn=rs758251988&dispmax=1&currpage=1) | R [Arg] ⇒ S [Ser] | 0.000008 |
| [rs758545398](https://www.ncbi.nlm.nih.gov/projects/SNP/snp_ref.cgi?rs=758545398) | GAT ⇒ AAT | [NP_473455.2](https://www.ncbi.nlm.nih.gov/entrez/query.fcgi?cmd=Search&db=protein&term=NP_473455.2) | [29](https://www.ncbi.nlm.nih.gov/sites/protein/NP_473455.2?report=graph&v=1:79&content=5&m=29%21&mn=rs758545398&dispmax=1&currpage=1) | D [Asp] ⇒ N [Asn] | 0.00002 |
| [rs758731322](https://www.ncbi.nlm.nih.gov/projects/SNP/snp_ref.cgi?rs=758731322) | GCT ⇒ GAT | [NP_473455.2](https://www.ncbi.nlm.nih.gov/entrez/query.fcgi?cmd=Search&db=protein&term=NP_001265662.1) | [339](https://www.ncbi.nlm.nih.gov/sites/protein/NP_001265662.1?report=graph&v=226:326&content=5&m=276%21&mn=rs758731322&dispmax=1&currpage=1) | A [Ala] ⇒ D [Asp] | 0.00002 |
| [rs758932155](https://www.ncbi.nlm.nih.gov/projects/SNP/snp_ref.cgi?rs=758932155) | CGT ⇒ CAT | [NP_473455.2](https://www.ncbi.nlm.nih.gov/entrez/query.fcgi?cmd=Search&db=protein&term=NP_473455.2) | [412](https://www.ncbi.nlm.nih.gov/sites/protein/NP_001265662.1?report=graph&v=299:399&content=5&m=349%21&mn=rs758932155&dispmax=1&currpage=1) | R [Arg] ⇒ H [His] | 0.000008 |
| [rs758985082](https://www.ncbi.nlm.nih.gov/projects/SNP/snp_ref.cgi?rs=758985082) | CTC ⇒ CGC | [NP_473455.2](https://www.ncbi.nlm.nih.gov/entrez/query.fcgi?cmd=Search&db=protein&term=NP_473455.2) | [388](https://www.ncbi.nlm.nih.gov/sites/protein/NP_001265662.1?report=graph&v=275:375&content=5&m=325%21&mn=rs758985082&dispmax=1&currpage=1) | L [Leu] ⇒ R [Arg] | 0.000008 |
| [rs759087022](https://www.ncbi.nlm.nih.gov/projects/SNP/snp_ref.cgi?rs=759087022) | AGT ⇒ AAT | [NP_473455.2](https://www.ncbi.nlm.nih.gov/entrez/query.fcgi?cmd=Search&db=protein&term=NP_473455.2) | [479](https://www.ncbi.nlm.nih.gov/sites/protein/NP_001265662.1?report=graph&v=366:466&content=5&m=416%21&mn=rs759087022&dispmax=1&currpage=1) | S [Ser] ⇒ N [Asn] | 0.00002 |
| [rs759151892](https://www.ncbi.nlm.nih.gov/projects/SNP/snp_ref.cgi?rs=759151892) | CCT ⇒ CGT | [NP_473455.2](https://www.ncbi.nlm.nih.gov/entrez/query.fcgi?cmd=Search&db=protein&term=NP_473455.2) | [515](https://www.ncbi.nlm.nih.gov/sites/protein/NP_001265662.1?report=graph&v=402:502&content=5&m=452%21&mn=rs759151892&dispmax=1&currpage=1) | P [Pro] ⇒ R [Arg] | 0.00002 |
| [rs759308422](https://www.ncbi.nlm.nih.gov/projects/SNP/snp_ref.cgi?rs=759308422) | CGA ⇒ CAA | [NP_473455.2](https://www.ncbi.nlm.nih.gov/entrez/query.fcgi?cmd=Search&db=protein&term=NP_001265662.1) | [712](https://www.ncbi.nlm.nih.gov/sites/protein/NP_001265662.1?report=graph&v=599:699&content=5&m=649%21&mn=rs759308422&dispmax=1&currpage=1) | R [Arg] ⇒ Q [Gln] | 0.000008 |
| [rs759674898](https://www.ncbi.nlm.nih.gov/projects/SNP/snp_ref.cgi?rs=759674898) | CCC ⇒ CTC | [NP_473455.2](https://www.ncbi.nlm.nih.gov/entrez/query.fcgi?cmd=Search&db=protein&term=NP_473455.2) | [166](https://www.ncbi.nlm.nih.gov/sites/protein/NP_001265662.1?report=graph&v=53:153&content=5&m=103%21&mn=rs759674898&dispmax=1&currpage=1) | P [Pro] ⇒ L [Leu] | 0.000008 |
| [rs759841143](https://www.ncbi.nlm.nih.gov/projects/SNP/snp_ref.cgi?rs=759841143) | GCC ⇒ ACC | [NP_473455.2](https://www.ncbi.nlm.nih.gov/entrez/query.fcgi?cmd=Search&db=protein&term=NP_473455.2) | [363](https://www.ncbi.nlm.nih.gov/sites/protein/NP_001265662.1?report=graph&v=250:350&content=5&m=300%21&mn=rs759841143&dispmax=1&currpage=1) | A [Ala] ⇒ T [Thr] | 0.00002 |
| [rs760010198](https://www.ncbi.nlm.nih.gov/projects/SNP/snp_ref.cgi?rs=760010198) | ACT ⇒ TCT | [NP_473455.2](https://www.ncbi.nlm.nih.gov/entrez/query.fcgi?cmd=Search&db=protein&term=NP_473455.2) | [296](https://www.ncbi.nlm.nih.gov/sites/protein/NP_001265662.1?report=graph&v=183:283&content=5&m=233%21&mn=rs760010198&dispmax=1&currpage=1) | T [Thr] ⇒ S [Ser] | 0.000008 |
| [rs760383023](https://www.ncbi.nlm.nih.gov/projects/SNP/snp_ref.cgi?rs=760383023) | CAC ⇒ CAG | [NP_473455.2](https://www.ncbi.nlm.nih.gov/entrez/query.fcgi?cmd=Search&db=protein&term=NP_473455.2) | [562](https://www.ncbi.nlm.nih.gov/sites/protein/NP_001265662.1?report=graph&v=449:549&content=5&m=499%21&mn=rs760383023&dispmax=1&currpage=1) | H [His] ⇒ Q [Gln] | 0.00003 |
| [rs760436084](https://www.ncbi.nlm.nih.gov/projects/SNP/snp_ref.cgi?rs=760436084) | AAC ⇒ AGC | [NP_473455.2](https://www.ncbi.nlm.nih.gov/entrez/query.fcgi?cmd=Search&db=protein&term=NP_473455.2) | [523](https://www.ncbi.nlm.nih.gov/sites/protein/NP_001265662.1?report=graph&v=410:510&content=5&m=460%21&mn=rs760436084&dispmax=1&currpage=1) | N [Asn] ⇒ S [Ser] | 0.000008 |
| [rs760957794](https://www.ncbi.nlm.nih.gov/projects/SNP/snp_ref.cgi?rs=760957794) | GTG ⇒ GGG | [NP_473455.2](https://www.ncbi.nlm.nih.gov/entrez/query.fcgi?cmd=Search&db=protein&term=NP_473455.2) | [430](https://www.ncbi.nlm.nih.gov/sites/protein/NP_001265662.1?report=graph&v=317:417&content=5&m=367%21&mn=rs760957794&dispmax=1&currpage=1) | V [Val] ⇒ G [Gly] | 0.00002 |
| [rs761095052](https://www.ncbi.nlm.nih.gov/projects/SNP/snp_ref.cgi?rs=761095052) | GCT ⇒ CCT | [NP_473455.2](https://www.ncbi.nlm.nih.gov/entrez/query.fcgi?cmd=Search&db=protein&term=NP_473455.2) | [339](https://www.ncbi.nlm.nih.gov/sites/protein/NP_001265662.1?report=graph&v=226:326&content=5&m=276%21&mn=rs761095052&dispmax=1&currpage=1) | A [Ala] ⇒ P [Pro] | 0.000008 |
| [rs761121568](https://www.ncbi.nlm.nih.gov/projects/SNP/snp_ref.cgi?rs=761121568) | TCA ⇒ CCA | [NP_473455.2](https://www.ncbi.nlm.nih.gov/entrez/query.fcgi?cmd=Search&db=protein&term=NP_473455.2) | [308](https://www.ncbi.nlm.nih.gov/sites/protein/NP_001265662.1?report=graph&v=195:295&content=5&m=245%21&mn=rs761121568&dispmax=1&currpage=1) | S [Ser] ⇒ P [Pro] | 0.000008 |
| [rs761171144](https://www.ncbi.nlm.nih.gov/projects/SNP/snp_ref.cgi?rs=761171144) | GAC ⇒ AAC | [NP_473455.2](https://www.ncbi.nlm.nih.gov/entrez/query.fcgi?cmd=Search&db=protein&term=NP_473455.2) | [96](https://www.ncbi.nlm.nih.gov/sites/protein/NP_001265662.1?report=graph&v=1:83&content=5&m=33%21&mn=rs761171144&dispmax=1&currpage=1) | D [Asp] ⇒ N [Asn] | 0.000008 |
| [rs761505429](https://www.ncbi.nlm.nih.gov/projects/SNP/snp_ref.cgi?rs=761505429) | CAT ⇒ CGT | [NP_473455.2](https://www.ncbi.nlm.nih.gov/entrez/query.fcgi?cmd=Search&db=protein&term=NP_473455.2) | [284](https://www.ncbi.nlm.nih.gov/sites/protein/NP_001265662.1?report=graph&v=171:271&content=5&m=221%21&mn=rs761505429&dispmax=1&currpage=1) | H [His] ⇒ R [Arg] | 0.000008 |
| [rs761720466](https://www.ncbi.nlm.nih.gov/projects/SNP/snp_ref.cgi?rs=761720466) | ATC ⇒ GTC | [NP_473455.2](https://www.ncbi.nlm.nih.gov/entrez/query.fcgi?cmd=Search&db=protein&term=NP_473455.2) | [442](https://www.ncbi.nlm.nih.gov/sites/protein/NP_001265662.1?report=graph&v=329:429&content=5&m=379%21&mn=rs761720466&dispmax=1&currpage=1) | I [Ile] ⇒ V [Val] | 0.000008 |
| [rs762424596](https://www.ncbi.nlm.nih.gov/projects/SNP/snp_ref.cgi?rs=762424596) | CCG ⇒ TCG | [NP_473455.2](https://www.ncbi.nlm.nih.gov/entrez/query.fcgi?cmd=Search&db=protein&term=NP_473455.2) | [447](https://www.ncbi.nlm.nih.gov/sites/protein/NP_001265662.1?report=graph&v=334:434&content=5&m=384%21&mn=rs762424596&dispmax=1&currpage=1) | P [Pro] ⇒ S [Ser] | 0.000008 |
| [rs762473635](https://www.ncbi.nlm.nih.gov/projects/SNP/snp_ref.cgi?rs=762473635) | TGC ⇒ CGC | [NP_473455.2](https://www.ncbi.nlm.nih.gov/entrez/query.fcgi?cmd=Search&db=protein&term=NP_473455.2) | [94](https://www.ncbi.nlm.nih.gov/sites/protein/NP_001265662.1?report=graph&v=1:81&content=5&m=31%21&mn=rs762473635&dispmax=1&currpage=1) | C [Cys] ⇒ R [Arg] | 0.000008 |
| [rs762524950](https://www.ncbi.nlm.nih.gov/projects/SNP/snp_ref.cgi?rs=762524950) | TCG ⇒ TTG | [NP_473455.2](https://www.ncbi.nlm.nih.gov/entrez/query.fcgi?cmd=Search&db=protein&term=NP_473455.2) | [302](https://www.ncbi.nlm.nih.gov/sites/protein/NP_001265662.1?report=graph&v=189:289&content=5&m=239%21&mn=rs762524950&dispmax=1&currpage=1) | S [Ser] ⇒ L [Leu] | 0.00008 |
| [rs762723607](https://www.ncbi.nlm.nih.gov/projects/SNP/snp_ref.cgi?rs=762723607) | AAC ⇒ AGC | [NP_473455.2](https://www.ncbi.nlm.nih.gov/entrez/query.fcgi?cmd=Search&db=protein&term=NP_473455.2) | [14](https://www.ncbi.nlm.nih.gov/sites/protein/NP_473455.2?report=graph&v=1:64&content=5&m=14%21&mn=rs762723607&dispmax=1&currpage=1) | N [Asn] ⇒ S [Ser] | 0.00002 |
| [rs762747475](https://www.ncbi.nlm.nih.gov/projects/SNP/snp_ref.cgi?rs=762747475) | TTC ⇒ TCC | [NP_473455.2](https://www.ncbi.nlm.nih.gov/entrez/query.fcgi?cmd=Search&db=protein&term=NP_473455.2) | [511](https://www.ncbi.nlm.nih.gov/sites/protein/NP_001265662.1?report=graph&v=398:498&content=5&m=448%21&mn=rs762747475&dispmax=1&currpage=1) | F [Phe] ⇒ S [Ser] | 0.000008 |
| [rs762949562](https://www.ncbi.nlm.nih.gov/projects/SNP/snp_ref.cgi?rs=762949562) | GTG ⇒ ATG | [NP_473455.2](https://www.ncbi.nlm.nih.gov/entrez/query.fcgi?cmd=Search&db=protein&term=NP_473455.2) | [474](https://www.ncbi.nlm.nih.gov/sites/protein/NP_001265662.1?report=graph&v=361:461&content=5&m=411%21&mn=rs762949562&dispmax=1&currpage=1) | V [Val] ⇒ M [Met] | 0.000008 |
| [rs763294731](https://www.ncbi.nlm.nih.gov/projects/SNP/snp_ref.cgi?rs=763294731) | AGA ⇒ ATA | [NP_473455.2](https://www.ncbi.nlm.nih.gov/entrez/query.fcgi?cmd=Search&db=protein&term=NP_473455.2) | [688](https://www.ncbi.nlm.nih.gov/sites/protein/NP_001265662.1?report=graph&v=575:675&content=5&m=625%21&mn=rs763294731&dispmax=1&currpage=1) | R [Arg] ⇒ I [Ile] | 0.000008 |
| [rs763380333](https://www.ncbi.nlm.nih.gov/projects/SNP/snp_ref.cgi?rs=763380333) | TTC ⇒ CTC | [NP_473455.2](https://www.ncbi.nlm.nih.gov/entrez/query.fcgi?cmd=Search&db=protein&term=NP_473455.2) | [122](https://www.ncbi.nlm.nih.gov/sites/protein/NP_001265662.1?report=graph&v=9:109&content=5&m=59%21&mn=rs763380333&dispmax=1&currpage=1) | F [Phe] ⇒ L [Leu] | 0.000008 |
| [rs763437025](https://www.ncbi.nlm.nih.gov/projects/SNP/snp_ref.cgi?rs=763437025) | TGC ⇒ CGC | [NP_473455.2](https://www.ncbi.nlm.nih.gov/entrez/query.fcgi?cmd=Search&db=protein&term=NP_473455.2) | [570](https://www.ncbi.nlm.nih.gov/sites/protein/NP_001265662.1?report=graph&v=457:557&content=5&m=507%21&mn=rs763437025&dispmax=1&currpage=1) | C [Cys] ⇒ R [Arg] | 0.00002 |
| [rs763905521](https://www.ncbi.nlm.nih.gov/projects/SNP/snp_ref.cgi?rs=763905521) | TCT ⇒ TTT | [NP_473455.2](https://www.ncbi.nlm.nih.gov/entrez/query.fcgi?cmd=Search&db=protein&term=NP_473455.2) | [510](https://www.ncbi.nlm.nih.gov/sites/protein/NP_001265662.1?report=graph&v=397:497&content=5&m=447%21&mn=rs763905521&dispmax=1&currpage=1) | S [Ser] ⇒ F [Phe] | 0.000008 |
| [rs764199475](https://www.ncbi.nlm.nih.gov/projects/SNP/snp_ref.cgi?rs=764199475) | CCC ⇒ ACC | [NP_473455.2](https://www.ncbi.nlm.nih.gov/entrez/query.fcgi?cmd=Search&db=protein&term=NP_001265662.1) | [357](https://www.ncbi.nlm.nih.gov/sites/protein/NP_001265662.1?report=graph&v=244:344&content=5&m=294%21&mn=rs764199475&dispmax=1&currpage=1) | P [Pro] ⇒ T [Thr] | 0.00003 |
| [rs764244240](https://www.ncbi.nlm.nih.gov/projects/SNP/snp_ref.cgi?rs=764244240) | GGT ⇒ GTT | [NP_473455.2](https://www.ncbi.nlm.nih.gov/entrez/query.fcgi?cmd=Search&db=protein&term=NP_473455.2) | [28](https://www.ncbi.nlm.nih.gov/sites/protein/NP_473455.2?report=graph&v=1:78&content=5&m=28%21&mn=rs764244240&dispmax=1&currpage=1) | G [Gly] ⇒ V [Val] | 0.000008 |
| rs764341891 | GGG ⇒ GCG | [NP_473455.2](https://www.ncbi.nlm.nih.gov/entrez/query.fcgi?cmd=Search&db=protein&term=NP_473455.2) | [687](https://www.ncbi.nlm.nih.gov/sites/protein/NP_001265662.1?report=graph&v=574:674&content=5&m=624%21&mn=rs764341891&dispmax=1&currpage=1) | G [Gly] ⇒ A [Ala] | 0.000008 |
| [rs764365414](https://www.ncbi.nlm.nih.gov/projects/SNP/snp_ref.cgi?rs=764365414) | CGC ⇒ CAC | [NP_473455.2](https://www.ncbi.nlm.nih.gov/entrez/query.fcgi?cmd=Search&db=protein&term=NP_473455.2) | [567](https://www.ncbi.nlm.nih.gov/sites/protein/NP_001265662.1?report=graph&v=454:554&content=5&m=504%21&mn=rs764365414&dispmax=1&currpage=1) | R [Arg] ⇒ H [His] | 0.00005 |
| [rs764717611](https://www.ncbi.nlm.nih.gov/projects/SNP/snp_ref.cgi?rs=764717611) | GGG ⇒ GAG | [NP_473455.2](https://www.ncbi.nlm.nih.gov/entrez/query.fcgi?cmd=Search&db=protein&term=NP_473455.2) | [120](https://www.ncbi.nlm.nih.gov/sites/protein/NP_001265662.1?report=graph&v=7:107&content=5&m=57%21&mn=rs764717611&dispmax=1&currpage=1) | G [Gly] ⇒ E [Glu] | 0.00002 |
| [rs764849840](https://www.ncbi.nlm.nih.gov/projects/SNP/snp_ref.cgi?rs=764849840) | AGG ⇒ ACG | [NP_473455.2](https://www.ncbi.nlm.nih.gov/entrez/query.fcgi?cmd=Search&db=protein&term=NP_473455.2) | [227](https://www.ncbi.nlm.nih.gov/sites/protein/NP_001265662.1?report=graph&v=114:214&content=5&m=164%21&mn=rs764849840&dispmax=1&currpage=1) | R [Arg] ⇒ T [Thr] | 0.00004 |
| [rs764960822](https://www.ncbi.nlm.nih.gov/projects/SNP/snp_ref.cgi?rs=764960822) | GAA ⇒ GCA | [NP_473455.2](https://www.ncbi.nlm.nih.gov/entrez/query.fcgi?cmd=Search&db=protein&term=NP_473455.2) | [32](https://www.ncbi.nlm.nih.gov/sites/protein/NP_473455.2?report=graph&v=1:82&content=5&m=32%21&mn=rs764960822&dispmax=1&currpage=1) | E [Glu] ⇒ A [Ala] | 0.000008 |
| [rs765083633](https://www.ncbi.nlm.nih.gov/projects/SNP/snp_ref.cgi?rs=765083633) | AAC ⇒ AAG | [NP_473455.2](https://www.ncbi.nlm.nih.gov/entrez/query.fcgi?cmd=Search&db=protein&term=NP_473455.2) | [260](https://www.ncbi.nlm.nih.gov/sites/protein/NP_001265662.1?report=graph&v=147:247&content=5&m=197%21&mn=rs765083633&dispmax=1&currpage=1) | N [Asn] ⇒ K [Lys] | 0.000008 |
| [rs765146154](https://www.ncbi.nlm.nih.gov/projects/SNP/snp_ref.cgi?rs=765146154) | AAC ⇒ AGC | [NP_473455.2](https://www.ncbi.nlm.nih.gov/entrez/query.fcgi?cmd=Search&db=protein&term=NP_473455.2) | [205](https://www.ncbi.nlm.nih.gov/sites/protein/NP_001265662.1?report=graph&v=92:192&content=5&m=142%21&mn=rs765146154&dispmax=1&currpage=1) | N [Asn] ⇒ S [Ser] | 0.000008 |
| [rs765384721](https://www.ncbi.nlm.nih.gov/projects/SNP/snp_ref.cgi?rs=765384721) | GAA ⇒ GAC | [NP_473455.2](https://www.ncbi.nlm.nih.gov/entrez/query.fcgi?cmd=Search&db=protein&term=NP_473455.2) | [414](https://www.ncbi.nlm.nih.gov/sites/protein/NP_001265662.1?report=graph&v=301:401&content=5&m=351%21&mn=rs765384721&dispmax=1&currpage=1) | E [Glu] ⇒ D [Asp] | 0.000008 |
| [rs765909071](https://www.ncbi.nlm.nih.gov/projects/SNP/snp_ref.cgi?rs=765909071) | TTC ⇒ GTC | [NP_473455.2](https://www.ncbi.nlm.nih.gov/entrez/query.fcgi?cmd=Search&db=protein&term=NP_473455.2) | [161](https://www.ncbi.nlm.nih.gov/sites/protein/NP_001265662.1?report=graph&v=48:148&content=5&m=98%21&mn=rs765909071&dispmax=1&currpage=1) | F [Phe] ⇒ V [Val] | 0.000008 |
| [rs766011091](https://www.ncbi.nlm.nih.gov/projects/SNP/snp_ref.cgi?rs=766011091) | GAA ⇒ AAA | [NP_473455.2](https://www.ncbi.nlm.nih.gov/entrez/query.fcgi?cmd=Search&db=protein&term=NP_473455.2) | [556](https://www.ncbi.nlm.nih.gov/sites/protein/NP_001265662.1?report=graph&v=443:543&content=5&m=493%21&mn=rs766011091&dispmax=1&currpage=1) | E [Glu] ⇒ K [Lys] | 0.00003 |
| [rs766166426](https://www.ncbi.nlm.nih.gov/projects/SNP/snp_ref.cgi?rs=766166426) | ACA ⇒ CCA | [NP_473455.2](https://www.ncbi.nlm.nih.gov/entrez/query.fcgi?cmd=Search&db=protein&term=NP_473455.2) | [398](https://www.ncbi.nlm.nih.gov/sites/protein/NP_001265662.1?report=graph&v=285:385&content=5&m=335%21&mn=rs766166426&dispmax=1&currpage=1) | T [Thr] ⇒ P [Pro] | 0.000008 |
| [rs766288589](https://www.ncbi.nlm.nih.gov/projects/SNP/snp_ref.cgi?rs=766288589) | GAC ⇒ GGC | [NP_473455.2](https://www.ncbi.nlm.nih.gov/entrez/query.fcgi?cmd=Search&db=protein&term=NP_473455.2) | [43](https://www.ncbi.nlm.nih.gov/sites/protein/NP_473455.2?report=graph&v=1:93&content=5&m=43%21&mn=rs766288589&dispmax=1&currpage=1) | D [Asp] ⇒ G [Gly] | 0.000008 |
| [rs766699345](https://www.ncbi.nlm.nih.gov/projects/SNP/snp_ref.cgi?rs=766699345) | ATG ⇒ AGG | [NP_473455.2](https://www.ncbi.nlm.nih.gov/entrez/query.fcgi?cmd=Search&db=protein&term=NP_473455.2) | [336](https://www.ncbi.nlm.nih.gov/sites/protein/NP_001265662.1?report=graph&v=223:323&content=5&m=273%21&mn=rs766699345&dispmax=1&currpage=1) | M [Met] ⇒ R [Arg] | 0.000008 |
| [rs766760704](https://www.ncbi.nlm.nih.gov/projects/SNP/snp_ref.cgi?rs=766760704) | CAG ⇒ CCG | [NP_473455.2](https://www.ncbi.nlm.nih.gov/entrez/query.fcgi?cmd=Search&db=protein&term=NP_473455.2) | [305](https://www.ncbi.nlm.nih.gov/sites/protein/NP_001265662.1?report=graph&v=192:292&content=5&m=242%21&mn=rs766760704&dispmax=1&currpage=1) | Q [Gln] ⇒ P [Pro] | 0.00002 |
| [rs767135924](https://www.ncbi.nlm.nih.gov/projects/SNP/snp_ref.cgi?rs=767135924) | CCG ⇒ GCG | [NP_473455.2](https://www.ncbi.nlm.nih.gov/entrez/query.fcgi?cmd=Search&db=protein&term=NP_473455.2) | [603](https://www.ncbi.nlm.nih.gov/sites/protein/NP_001265662.1?report=graph&v=490:590&content=5&m=540%21&mn=rs767135924&dispmax=1&currpage=1) | P [Pro] ⇒ A [Ala] | 0.00003 |
| [rs767627653](https://www.ncbi.nlm.nih.gov/projects/SNP/snp_ref.cgi?rs=767627653) | GCA ⇒ GTA | [NP_473455.2](https://www.ncbi.nlm.nih.gov/entrez/query.fcgi?cmd=Search&db=protein&term=NP_473455.2) | [83](https://www.ncbi.nlm.nih.gov/sites/protein/NP_001265662.1?report=graph&v=1:70&content=5&m=20%21&mn=rs767627653&dispmax=1&currpage=1) | A [Ala] ⇒ V [Val] | 0.000008 |
| [rs767628374](https://www.ncbi.nlm.nih.gov/projects/SNP/snp_ref.cgi?rs=767628374) | GGG ⇒ AGG | [NP_473455.2](https://www.ncbi.nlm.nih.gov/entrez/query.fcgi?cmd=Search&db=protein&term=NP_473455.2) | [681](https://www.ncbi.nlm.nih.gov/sites/protein/NP_001265662.1?report=graph&v=568:668&content=5&m=618%21&mn=rs767628374&dispmax=1&currpage=1) | G [Gly] ⇒ R [Arg] | 0.000008 |
| [rs767642705](https://www.ncbi.nlm.nih.gov/projects/SNP/snp_ref.cgi?rs=767642705) | GAT ⇒ AAT | [NP_473455.2](https://www.ncbi.nlm.nih.gov/entrez/query.fcgi?cmd=Search&db=protein&term=NP_473455.2) | [199](https://www.ncbi.nlm.nih.gov/sites/protein/NP_001265662.1?report=graph&v=86:186&content=5&m=136%21&mn=rs767642705&dispmax=1&currpage=1) | D [Asp] ⇒ N [Asn] | 0.000008 |
| [rs767852044](https://www.ncbi.nlm.nih.gov/projects/SNP/snp_ref.cgi?rs=767852044) | GAC ⇒ AAC | [NP_473455.2](https://www.ncbi.nlm.nih.gov/entrez/query.fcgi?cmd=Search&db=protein&term=NP_473455.2) | [707](https://www.ncbi.nlm.nih.gov/sites/protein/NP_001265662.1?report=graph&v=594:694&content=5&m=644%21&mn=rs767852044&dispmax=1&currpage=1) | D [Asp] ⇒ N [Asn] | 0.000008 |
| [rs768555725](https://www.ncbi.nlm.nih.gov/projects/SNP/snp_ref.cgi?rs=768555725) | GGC ⇒ AGC | [NP_473455.2](https://www.ncbi.nlm.nih.gov/entrez/query.fcgi?cmd=Search&db=protein&term=NP_473455.2) | [657](https://www.ncbi.nlm.nih.gov/sites/protein/NP_001265662.1?report=graph&v=544:644&content=5&m=594%21&mn=rs768555725&dispmax=1&currpage=1) | G [Gly] ⇒ S [Ser] | 0.00002 |
| [rs768645169](https://www.ncbi.nlm.nih.gov/projects/SNP/snp_ref.cgi?rs=768645169) | TTG ⇒ TTC | [NP_473455.2](https://www.ncbi.nlm.nih.gov/entrez/query.fcgi?cmd=Search&db=protein&term=NP_001265662.1) | [342](https://www.ncbi.nlm.nih.gov/sites/protein/NP_001265662.1?report=graph&v=229:329&content=5&m=279%21&mn=rs768645169&dispmax=1&currpage=1) | L [Leu] ⇒ F [Phe] | 0.000008 |
| [rs769005032](https://www.ncbi.nlm.nih.gov/projects/SNP/snp_ref.cgi?rs=769005032) | TCT ⇒ CCT | [NP_473455.2](https://www.ncbi.nlm.nih.gov/entrez/query.fcgi?cmd=Search&db=protein&term=NP_473455.2) | [490](https://www.ncbi.nlm.nih.gov/sites/protein/NP_001265662.1?report=graph&v=377:477&content=5&m=427%21&mn=rs769005032&dispmax=1&currpage=1) | S [Ser] ⇒ P [Pro] | 0.000008 |
| [rs769115087](https://www.ncbi.nlm.nih.gov/projects/SNP/snp_ref.cgi?rs=769115087) | GCG ⇒ ACG | [NP_473455.2](https://www.ncbi.nlm.nih.gov/entrez/query.fcgi?cmd=Search&db=protein&term=NP_473455.2) | [526](https://www.ncbi.nlm.nih.gov/sites/protein/NP_001265662.1?report=graph&v=413:513&content=5&m=463%21&mn=rs769115087&dispmax=1&currpage=1) | A [Ala] ⇒ T [Thr] | 0.00002 |
| [rs769704785](https://www.ncbi.nlm.nih.gov/projects/SNP/snp_ref.cgi?rs=769704785) | AGC ⇒ AGA | [NP_473455.2](https://www.ncbi.nlm.nih.gov/entrez/query.fcgi?cmd=Search&db=protein&term=NP_001265662.1) | [344](https://www.ncbi.nlm.nih.gov/sites/protein/NP_001265662.1?report=graph&v=231:331&content=5&m=281%21&mn=rs769704785&dispmax=1&currpage=1) | S [Ser] ⇒ R [Arg] | 0.0003 |
| [rs769776999](https://www.ncbi.nlm.nih.gov/projects/SNP/snp_ref.cgi?rs=769776999) | AGA ⇒ AAA | [NP_473455.2](https://www.ncbi.nlm.nih.gov/entrez/query.fcgi?cmd=Search&db=protein&term=NP_473455.2) | [4](https://www.ncbi.nlm.nih.gov/sites/protein/NP_473455.2?report=graph&v=1:54&content=5&m=4%21&mn=rs769776999&dispmax=1&currpage=1) | R [Arg] ⇒ K [Lys] | 0.000008 |
| [rs770272903](https://www.ncbi.nlm.nih.gov/projects/SNP/snp_ref.cgi?rs=770272903) | CAC ⇒ AAC | [NP_473455.2](https://www.ncbi.nlm.nih.gov/entrez/query.fcgi?cmd=Search&db=protein&term=NP_473455.2) | [574](https://www.ncbi.nlm.nih.gov/sites/protein/NP_001265662.1?report=graph&v=461:561&content=5&m=511%21&mn=rs770272903&dispmax=1&currpage=1) | H [His] ⇒ N [Asn] | 0.000008 |
| [rs770463047](https://www.ncbi.nlm.nih.gov/projects/SNP/snp_ref.cgi?rs=770463047) | TCC ⇒ TTC | [NP_473455.2](https://www.ncbi.nlm.nih.gov/entrez/query.fcgi?cmd=Search&db=protein&term=NP_473455.2) | [698](https://www.ncbi.nlm.nih.gov/sites/protein/NP_001265662.1?report=graph&v=585:685&content=5&m=635%21&mn=rs770463047&dispmax=1&currpage=1) | S [Ser] ⇒ F [Phe] | 0.000008 |
| [rs770707646](https://www.ncbi.nlm.nih.gov/projects/SNP/snp_ref.cgi?rs=770707646) | TGT ⇒ CGT | [NP_473455.2](https://www.ncbi.nlm.nih.gov/entrez/query.fcgi?cmd=Search&db=protein&term=NP_001265662.1) | [713](https://www.ncbi.nlm.nih.gov/sites/protein/NP_001265662.1?report=graph&v=600:700&content=5&m=650%21&mn=rs770707646&dispmax=1&currpage=1) | C [Cys] ⇒ R [Arg] | 0.0001 |
| [rs770816984](https://www.ncbi.nlm.nih.gov/projects/SNP/snp_ref.cgi?rs=770816984) | GGG ⇒ GCG | [NP_473455.2](https://www.ncbi.nlm.nih.gov/entrez/query.fcgi?cmd=Search&db=protein&term=NP_473455.2) | [141](https://www.ncbi.nlm.nih.gov/sites/protein/NP_001265662.1?report=graph&v=28:128&content=5&m=78%21&mn=rs770816984&dispmax=1&currpage=1) | G [Gly] ⇒ A [Ala] | 0.00003 |
| [rs771175774](https://www.ncbi.nlm.nih.gov/projects/SNP/snp_ref.cgi?rs=771175774) | GGT ⇒ AGT | [NP_473455.2](https://www.ncbi.nlm.nih.gov/entrez/query.fcgi?cmd=Search&db=protein&term=NP_473455.2) | [95](https://www.ncbi.nlm.nih.gov/sites/protein/NP_001265662.1?report=graph&v=1:82&content=5&m=32%21&mn=rs771175774&dispmax=1&currpage=1) | G [Gly] ⇒ S [Ser] | 0.00007 |
| [rs771357920](https://www.ncbi.nlm.nih.gov/projects/SNP/snp_ref.cgi?rs=771357920) | GTG ⇒ ATG | [NP_473455.2](https://www.ncbi.nlm.nih.gov/entrez/query.fcgi?cmd=Search&db=protein&term=NP_473455.2) | [57](https://www.ncbi.nlm.nih.gov/sites/protein/NP_473455.2?report=graph&v=7:107&content=5&m=57%21&mn=rs771357920&dispmax=1&currpage=1) | V [Val] ⇒ M [Met] | 0.000008 |
| [rs772135673](https://www.ncbi.nlm.nih.gov/projects/SNP/snp_ref.cgi?rs=772135673) | GAC ⇒ AAC | [NP_473455.2](https://www.ncbi.nlm.nih.gov/entrez/query.fcgi?cmd=Search&db=protein&term=NP_473455.2) | [311](https://www.ncbi.nlm.nih.gov/sites/protein/NP_001265662.1?report=graph&v=198:298&content=5&m=248%21&mn=rs772135673&dispmax=1&currpage=1) | D [Asp] ⇒ N [Asn] | 0.00002 |
| [rs772136081](https://www.ncbi.nlm.nih.gov/projects/SNP/snp_ref.cgi?rs=772136081) | CTC ⇒ CAC | [NP_473455.2](https://www.ncbi.nlm.nih.gov/entrez/query.fcgi?cmd=Search&db=protein&term=NP_473455.2) | [67](https://www.ncbi.nlm.nih.gov/sites/protein/NP_001265662.1?report=graph&v=1:54&content=5&m=4%21&mn=rs772136081&dispmax=1&currpage=1) | L [Leu] ⇒ H [His] | 0.000008 |
| [rs772560830](https://www.ncbi.nlm.nih.gov/projects/SNP/snp_ref.cgi?rs=772560830) | AAT ⇒ CAT | [NP_473455.2](https://www.ncbi.nlm.nih.gov/entrez/query.fcgi?cmd=Search&db=protein&term=NP_473455.2) | [483](https://www.ncbi.nlm.nih.gov/sites/protein/NP_001265662.1?report=graph&v=370:470&content=5&m=420%21&mn=rs772560830&dispmax=1&currpage=1) | N [Asn] ⇒ H [His] | 0.000008 |
| [rs773039880](https://www.ncbi.nlm.nih.gov/projects/SNP/snp_ref.cgi?rs=773039880) | AGC ⇒ GGC | [NP_473455.2](https://www.ncbi.nlm.nih.gov/entrez/query.fcgi?cmd=Search&db=protein&term=NP_473455.2) | [220](https://www.ncbi.nlm.nih.gov/sites/protein/NP_001265662.1?report=graph&v=107:207&content=5&m=157%21&mn=rs773039880&dispmax=1&currpage=1) | S [Ser] ⇒ G [Gly] | 0.000008 |
| [rs773091324](https://www.ncbi.nlm.nih.gov/projects/SNP/snp_ref.cgi?rs=773091324) | GCA ⇒ ACA | [NP_473455.2](https://www.ncbi.nlm.nih.gov/entrez/query.fcgi?cmd=Search&db=protein&term=NP_473455.2) | [370](https://www.ncbi.nlm.nih.gov/sites/protein/NP_001265662.1?report=graph&v=257:357&content=5&m=307%21&mn=rs773091324&dispmax=1&currpage=1) | A [Ala] ⇒ T [Thr] | 0.000008 |
| [rs773179199](https://www.ncbi.nlm.nih.gov/projects/SNP/snp_ref.cgi?rs=773179199) | ACG ⇒ ATG | [NP_473455.2](https://www.ncbi.nlm.nih.gov/entrez/query.fcgi?cmd=Search&db=protein&term=NP_473455.2) | [619](https://www.ncbi.nlm.nih.gov/sites/protein/NP_001265662.1?report=graph&v=506:606&content=5&m=556%21&mn=rs773179199&dispmax=1&currpage=1) | T [Thr] ⇒ M [Met] | 0.000008 |
| [rs773293336](https://www.ncbi.nlm.nih.gov/projects/SNP/snp_ref.cgi?rs=773293336) | GAC ⇒ GGC | [NP_473455.2](https://www.ncbi.nlm.nih.gov/entrez/query.fcgi?cmd=Search&db=protein&term=NP_001265662.1) | [645](https://www.ncbi.nlm.nih.gov/sites/protein/NP_001265662.1?report=graph&v=532:632&content=5&m=582%21&mn=rs773293336&dispmax=1&currpage=1) | D [Asp] ⇒ G [Gly] | 0.00002 |
| [rs773697806](https://www.ncbi.nlm.nih.gov/projects/SNP/snp_ref.cgi?rs=773697806) | GAC ⇒ AAC | [NP_473455.2](https://www.ncbi.nlm.nih.gov/entrez/query.fcgi?cmd=Search&db=protein&term=NP_473455.2) | [98](https://www.ncbi.nlm.nih.gov/sites/protein/NP_001265662.1?report=graph&v=1:85&content=5&m=35%21&mn=rs773697806&dispmax=1&currpage=1) | D [Asp] ⇒ N [Asn] | 0.000008 |
| [rs773804288](https://www.ncbi.nlm.nih.gov/projects/SNP/snp_ref.cgi?rs=773804288) | GTA ⇒ GCA | [NP_473455.2](https://www.ncbi.nlm.nih.gov/entrez/query.fcgi?cmd=Search&db=protein&term=NP_473455.2) | [409](https://www.ncbi.nlm.nih.gov/sites/protein/NP_001265662.1?report=graph&v=296:396&content=5&m=346%21&mn=rs773804288&dispmax=1&currpage=1) | V [Val] ⇒ A [Ala] | 0.000008 |
| [rs774098883](https://www.ncbi.nlm.nih.gov/projects/SNP/snp_ref.cgi?rs=774098883) | ACT ⇒ AGT | [NP_473455.2](https://www.ncbi.nlm.nih.gov/entrez/query.fcgi?cmd=Search&db=protein&term=NP_473455.2) | [288](https://www.ncbi.nlm.nih.gov/sites/protein/NP_001265662.1?report=graph&v=175:275&content=5&m=225%21&mn=rs774098883&dispmax=1&currpage=1) | T [Thr] ⇒ S [Ser] | 0.00003 |
| [rs774261461](https://www.ncbi.nlm.nih.gov/projects/SNP/snp_ref.cgi?rs=774261461) | GAC ⇒ AAC | [NP_473455.2](https://www.ncbi.nlm.nih.gov/entrez/query.fcgi?cmd=Search&db=protein&term=NP_473455.2) | [186](https://www.ncbi.nlm.nih.gov/sites/protein/NP_001265662.1?report=graph&v=73:173&content=5&m=123%21&mn=rs774261461&dispmax=1&currpage=1) | D [Asp] ⇒ N [Asn] | 0.00003 |
| [rs774310085](https://www.ncbi.nlm.nih.gov/projects/SNP/snp_ref.cgi?rs=774310085) | GAC ⇒ GAA | [NP_473455.2](https://www.ncbi.nlm.nih.gov/entrez/query.fcgi?cmd=Search&db=protein&term=NP_473455.2) | [314](https://www.ncbi.nlm.nih.gov/sites/protein/NP_001265662.1?report=graph&v=201:301&content=5&m=251%21&mn=rs774310085&dispmax=1&currpage=1) | D [Asp] ⇒ E [Glu] | 0.000008 |
| [rs774501392](https://www.ncbi.nlm.nih.gov/projects/SNP/snp_ref.cgi?rs=774501392) | CCT ⇒ TCT | [NP_473455.2](https://www.ncbi.nlm.nih.gov/entrez/query.fcgi?cmd=Search&db=protein&term=NP_473455.2) | [656](https://www.ncbi.nlm.nih.gov/sites/protein/NP_001265662.1?report=graph&v=543:643&content=5&m=593%21&mn=rs774501392&dispmax=1&currpage=1) | P [Pro] ⇒ S [Ser] | 0.000008 |
| [rs774820177](https://www.ncbi.nlm.nih.gov/projects/SNP/snp_ref.cgi?rs=774820177) | TCT ⇒ TTT | [NP_473455.2](https://www.ncbi.nlm.nih.gov/entrez/query.fcgi?cmd=Search&db=protein&term=NP_473455.2) | [476](https://www.ncbi.nlm.nih.gov/sites/protein/NP_001265662.1?report=graph&v=363:463&content=5&m=413%21&mn=rs774820177&dispmax=1&currpage=1) | S [Ser] ⇒ F [Phe] | 0.00003 |
| [rs775839075](https://www.ncbi.nlm.nih.gov/projects/SNP/snp_ref.cgi?rs=775839075) | AGC ⇒ CGC | [NP_473455.2](https://www.ncbi.nlm.nih.gov/entrez/query.fcgi?cmd=Search&db=protein&term=NP_473455.2) | [360](https://www.ncbi.nlm.nih.gov/sites/protein/NP_001265662.1?report=graph&v=247:347&content=5&m=297%21&mn=rs775839075&dispmax=1&currpage=1) | S [Ser] ⇒ R [Arg] | 0.000008 |
| [rs775898458](https://www.ncbi.nlm.nih.gov/projects/SNP/snp_ref.cgi?rs=775898458) | CGA ⇒ CAA | [NP_473455.2](https://www.ncbi.nlm.nih.gov/entrez/query.fcgi?cmd=Search&db=protein&term=NP_001265662.1) | [711](https://www.ncbi.nlm.nih.gov/sites/protein/NP_001265662.1?report=graph&v=598:698&content=5&m=648%21&mn=rs775898458&dispmax=1&currpage=1) | R [Arg] ⇒ Q [Gln] | 0.00002 |
| [rs776501479](https://www.ncbi.nlm.nih.gov/projects/SNP/snp_ref.cgi?rs=776501479) | AGG ⇒ AGT | [NP_473455.2](https://www.ncbi.nlm.nih.gov/entrez/query.fcgi?cmd=Search&db=protein&term=NP_473455.2) | [695](https://www.ncbi.nlm.nih.gov/sites/protein/NP_001265662.1?report=graph&v=582:682&content=5&m=632%21&mn=rs776501479&dispmax=1&currpage=1) | R [Arg] ⇒ S [Ser] | 0.00003 |
| [rs776525307](https://www.ncbi.nlm.nih.gov/projects/SNP/snp_ref.cgi?rs=776525307) | AGC ⇒ ATC | [NP_473455.2](https://www.ncbi.nlm.nih.gov/entrez/query.fcgi?cmd=Search&db=protein&term=NP_473455.2) | [367](https://www.ncbi.nlm.nih.gov/sites/protein/NP_001265662.1?report=graph&v=254:354&content=5&m=304%21&mn=rs776525307&dispmax=1&currpage=1) | S [Ser] ⇒ I [Ile] | 0.00002 |
| [rs776629257](https://www.ncbi.nlm.nih.gov/projects/SNP/snp_ref.cgi?rs=776629257) | GCA ⇒ GTA | [NP_473455.2](https://www.ncbi.nlm.nih.gov/entrez/query.fcgi?cmd=Search&db=protein&term=NP_473455.2) | [37](https://www.ncbi.nlm.nih.gov/sites/protein/NP_473455.2?report=graph&v=1:87&content=5&m=37%21&mn=rs776629257&dispmax=1&currpage=1) | A [Ala] ⇒ V [Val] | 0.000008 |
| [rs777042268](https://www.ncbi.nlm.nih.gov/projects/SNP/snp_ref.cgi?rs=777042268) | GGG ⇒ TGG | [NP_473455.2](https://www.ncbi.nlm.nih.gov/entrez/query.fcgi?cmd=Search&db=protein&term=NP_473455.2) | [141](https://www.ncbi.nlm.nih.gov/sites/protein/NP_001265662.1?report=graph&v=28:128&content=5&m=78%21&mn=rs777042268&dispmax=1&currpage=1) | G [Gly] ⇒ W [Trp] | 0.000008 |
| [rs777150394](https://www.ncbi.nlm.nih.gov/projects/SNP/snp_ref.cgi?rs=777150394) | GTG ⇒ ATG | [NP_473455.2](https://www.ncbi.nlm.nih.gov/entrez/query.fcgi?cmd=Search&db=protein&term=NP_473455.2) | [334](https://www.ncbi.nlm.nih.gov/sites/protein/NP_001265662.1?report=graph&v=221:321&content=5&m=271%21&mn=rs777150394&dispmax=1&currpage=1) | V [Val] ⇒ M [Met] | 0.00002 |
| [rs777164310](https://www.ncbi.nlm.nih.gov/projects/SNP/snp_ref.cgi?rs=777164310) | CAG ⇒ CAC | [NP_473455.2](https://www.ncbi.nlm.nih.gov/entrez/query.fcgi?cmd=Search&db=protein&term=NP_473455.2) | [613](https://www.ncbi.nlm.nih.gov/sites/protein/NP_001265662.1?report=graph&v=500:600&content=5&m=550%21&mn=rs777164310&dispmax=1&currpage=1) | Q [Gln] ⇒ H [His] | 0.000008 |
| [rs777270446](https://www.ncbi.nlm.nih.gov/projects/SNP/snp_ref.cgi?rs=777270446) | GAG ⇒ AAG | [NP_473455.2](https://www.ncbi.nlm.nih.gov/entrez/query.fcgi?cmd=Search&db=protein&term=NP_473455.2) | [699](https://www.ncbi.nlm.nih.gov/sites/protein/NP_001265662.1?report=graph&v=586:686&content=5&m=636%21&mn=rs777270446&dispmax=1&currpage=1) | E [Glu] ⇒ K [Lys] | 0.000008 |
| [rs777519361](https://www.ncbi.nlm.nih.gov/projects/SNP/snp_ref.cgi?rs=777519361) | GTC ⇒ ATC | [NP_473455.2](https://www.ncbi.nlm.nih.gov/entrez/query.fcgi?cmd=Search&db=protein&term=NP_473455.2) | [539](https://www.ncbi.nlm.nih.gov/sites/protein/NP_001265662.1?report=graph&v=426:526&content=5&m=476%21&mn=rs777519361&dispmax=1&currpage=1) | V [Val] ⇒ I [Ile] | 0.000008 |
| [rs778035400](https://www.ncbi.nlm.nih.gov/projects/SNP/snp_ref.cgi?rs=778035400) | GAC ⇒ AAC | [NP_473455.2](https://www.ncbi.nlm.nih.gov/entrez/query.fcgi?cmd=Search&db=protein&term=NP_473455.2) | [247](https://www.ncbi.nlm.nih.gov/sites/protein/NP_001265662.1?report=graph&v=134:234&content=5&m=184%21&mn=rs778035400&dispmax=1&currpage=1) | D [Asp] ⇒ N [Asn] | 0.000008 |
| [rs778299145](https://www.ncbi.nlm.nih.gov/projects/SNP/snp_ref.cgi?rs=778299145) | CTC ⇒ ATC | [NP_473455.2](https://www.ncbi.nlm.nih.gov/entrez/query.fcgi?cmd=Search&db=protein&term=NP_473455.2) | [67](https://www.ncbi.nlm.nih.gov/sites/protein/NP_001265662.1?report=graph&v=1:54&content=5&m=4%21&mn=rs778299145&dispmax=1&currpage=1) | L [Leu] ⇒ I [Ile] | 0.00003 |
| [rs778438807](https://www.ncbi.nlm.nih.gov/projects/SNP/snp_ref.cgi?rs=778438807) | GTG ⇒ ATG | [NP_473455.2](https://www.ncbi.nlm.nih.gov/entrez/query.fcgi?cmd=Search&db=protein&term=NP_473455.2) | [151](https://www.ncbi.nlm.nih.gov/sites/protein/NP_001265662.1?report=graph&v=38:138&content=5&m=88%21&mn=rs778438807&dispmax=1&currpage=1) | V [Val] ⇒ M [Met] | 0.00003 |
| [rs778593207](https://www.ncbi.nlm.nih.gov/projects/SNP/snp_ref.cgi?rs=778593207) | TTT ⇒ TCT | [NP_473455.2](https://www.ncbi.nlm.nih.gov/entrez/query.fcgi?cmd=Search&db=protein&term=NP_473455.2) | [718](https://www.ncbi.nlm.nih.gov/sites/protein/NP_001265662.1?report=graph&v=605:705&content=5&m=655%21&mn=rs778593207&dispmax=1&currpage=1) | F [Phe] ⇒ S [Ser] | 0.00002 |
| [rs778595389](https://www.ncbi.nlm.nih.gov/projects/SNP/snp_ref.cgi?rs=778595389) | GAT ⇒ GAA | [NP_473455.2](https://www.ncbi.nlm.nih.gov/entrez/query.fcgi?cmd=Search&db=protein&term=NP_473455.2) | [349](https://www.ncbi.nlm.nih.gov/sites/protein/NP_001265662.1?report=graph&v=236:336&content=5&m=286%21&mn=rs778595389&dispmax=1&currpage=1) | D [Asp] ⇒ E [Glu] | 0.00004 |
| [rs779425721](https://www.ncbi.nlm.nih.gov/projects/SNP/snp_ref.cgi?rs=779425721) | CAT ⇒ CAA | [NP_473455.2](https://www.ncbi.nlm.nih.gov/entrez/query.fcgi?cmd=Search&db=protein&term=NP_473455.2) | [488](https://www.ncbi.nlm.nih.gov/sites/protein/NP_001265662.1?report=graph&v=375:475&content=5&m=425%21&mn=rs779425721&dispmax=1&currpage=1) | H [His] ⇒ Q [Gln] | 0.000008 |
| [rs779630199](https://www.ncbi.nlm.nih.gov/projects/SNP/snp_ref.cgi?rs=779630199) | CTG ⇒ CCG | [NP_473455.2](https://www.ncbi.nlm.nih.gov/entrez/query.fcgi?cmd=Search&db=protein&term=NP_473455.2) | [455](https://www.ncbi.nlm.nih.gov/sites/protein/NP_001265662.1?report=graph&v=342:442&content=5&m=392%21&mn=rs779630199&dispmax=1&currpage=1) | L [Leu] ⇒ P [Pro] | 0.000008 |
| [rs779668504](https://www.ncbi.nlm.nih.gov/projects/SNP/snp_ref.cgi?rs=779668504) | GCT ⇒ ACT | [NP_473455.2](https://www.ncbi.nlm.nih.gov/entrez/query.fcgi?cmd=Search&db=protein&term=NP_473455.2) | [76](https://www.ncbi.nlm.nih.gov/sites/protein/NP_001265662.1?report=graph&v=1:63&content=5&m=13%21&mn=rs779668504&dispmax=1&currpage=1) | A [Ala] ⇒ T [Thr] | 0.00002 |
| [rs779949611](https://www.ncbi.nlm.nih.gov/projects/SNP/snp_ref.cgi?rs=779949611) | ATC ⇒ ACC | [NP_473455.2](https://www.ncbi.nlm.nih.gov/entrez/query.fcgi?cmd=Search&db=protein&term=NP_473455.2) | [325](https://www.ncbi.nlm.nih.gov/sites/protein/NP_001265662.1?report=graph&v=212:312&content=5&m=262%21&mn=rs779949611&dispmax=1&currpage=1) | I [Ile] ⇒ T [Thr] | 0.000008 |
| [rs779977381](https://www.ncbi.nlm.nih.gov/projects/SNP/snp_ref.cgi?rs=779977381) | ACA ⇒ ATA | [NP_473455.2](https://www.ncbi.nlm.nih.gov/entrez/query.fcgi?cmd=Search&db=protein&term=NP_473455.2) | [495](https://www.ncbi.nlm.nih.gov/sites/protein/NP_001265662.1?report=graph&v=382:482&content=5&m=432%21&mn=rs779977381&dispmax=1&currpage=1) | T [Thr] ⇒ I [Ile] | 0.000008 |
| [rs779996502](https://www.ncbi.nlm.nih.gov/projects/SNP/snp_ref.cgi?rs=779996502) | CTG ⇒ GTG | [NP_473455.2](https://www.ncbi.nlm.nih.gov/entrez/query.fcgi?cmd=Search&db=protein&term=NP_473455.2) | [663](https://www.ncbi.nlm.nih.gov/sites/protein/NP_001265662.1?report=graph&v=550:650&content=5&m=600%21&mn=rs779996502&dispmax=1&currpage=1) | L [Leu] ⇒ V [Val] | 0.000008 |
| [rs780364584](https://www.ncbi.nlm.nih.gov/projects/SNP/snp_ref.cgi?rs=780364584) | AGG ⇒ AGT | [NP_473455.2](https://www.ncbi.nlm.nih.gov/entrez/query.fcgi?cmd=Search&db=protein&term=NP_473455.2) | [703](https://www.ncbi.nlm.nih.gov/sites/protein/NP_001265662.1?report=graph&v=590:690&content=5&m=640%21&mn=rs780364584&dispmax=1&currpage=1) | R [Arg] ⇒ S [Ser] | 0.00002 |
| [rs780482642](https://www.ncbi.nlm.nih.gov/projects/SNP/snp_ref.cgi?rs=780482642) | GTG ⇒ ATG | [NP_473455.2](https://www.ncbi.nlm.nih.gov/entrez/query.fcgi?cmd=Search&db=protein&term=NP_473455.2) | [225](https://www.ncbi.nlm.nih.gov/sites/protein/NP_001265662.1?report=graph&v=112:212&content=5&m=162%21&mn=rs780482642&dispmax=1&currpage=1) | V [Val] ⇒ M [Met] | 0.00004 |
| [rs781016202](https://www.ncbi.nlm.nih.gov/projects/SNP/snp_ref.cgi?rs=781016202) | AGC ⇒ AAC | [NP_473455.2](https://www.ncbi.nlm.nih.gov/entrez/query.fcgi?cmd=Search&db=protein&term=NP_473455.2) | [172](https://www.ncbi.nlm.nih.gov/sites/protein/NP_001265662.1?report=graph&v=59:159&content=5&m=109%21&mn=rs781016202&dispmax=1&currpage=1) | S [Ser] ⇒ N [Asn] | 0.00005 |
| [rs781080965](https://www.ncbi.nlm.nih.gov/projects/SNP/snp_ref.cgi?rs=781080965) | ATG ⇒ AAG | [NP_473455.2](https://www.ncbi.nlm.nih.gov/entrez/query.fcgi?cmd=Search&db=protein&term=NP_473455.2) | [228](https://www.ncbi.nlm.nih.gov/sites/protein/NP_001265662.1?report=graph&v=115:215&content=5&m=165%21&mn=rs781080965&dispmax=1&currpage=1) | M [Met] ⇒ K [Lys] | 0.000008 |
| [rs781249991](https://www.ncbi.nlm.nih.gov/projects/SNP/snp_ref.cgi?rs=781249991) | ACC ⇒ ATC | [NP_473455.2](https://www.ncbi.nlm.nih.gov/entrez/query.fcgi?cmd=Search&db=protein&term=NP_473455.2) | [535](https://www.ncbi.nlm.nih.gov/sites/protein/NP_001265662.1?report=graph&v=422:522&content=5&m=472%21&mn=rs781249991&dispmax=1&currpage=1) | T [Thr] ⇒ I [Ile] | 0.000008 |
| [rs781342205](https://www.ncbi.nlm.nih.gov/projects/SNP/snp_ref.cgi?rs=781342205) | AGC ⇒ AAC | [NP_473455.2](https://www.ncbi.nlm.nih.gov/entrez/query.fcgi?cmd=Search&db=protein&term=NP_001265662.1) | [329](https://www.ncbi.nlm.nih.gov/sites/protein/NP_001265662.1?report=graph&v=216:316&content=5&m=266%21&mn=rs781342205&dispmax=1&currpage=1) | S [Ser] ⇒ N [Asn] | 0.000008 |
| [rs781602265](https://www.ncbi.nlm.nih.gov/projects/SNP/snp_ref.cgi?rs=781602265) | AGC ⇒ ACC | NP_473455.2 | [380](https://www.ncbi.nlm.nih.gov/sites/protein/NP_001265662.1?report=graph&v=267:367&content=5&m=317%21&mn=rs781602265&dispmax=1&currpage=1) | S [Ser] ⇒ T [Thr] | 0.000008 |
| rs905326257 | GA**C** ⇒ GA**G** | NP_473455.3 | [160](https://www.ncbi.nlm.nih.gov/sites/protein/NP_473455.2?report=graph&v=110:210&content=5&m=160!&mn=rs905326257&dispmax=1&currpage=1) | **D [Asp]**⇒ **E [Glu]** | 0.00003 |
| rs908964894 | AT**G** ⇒ AT**C** | NP_473455.4 | [627](https://www.ncbi.nlm.nih.gov/sites/protein/NP_473455.2?report=graph&v=577:677&content=5&m=627!&mn=rs908964894&dispmax=1&currpage=1) | **M [Met]**⇒ **I [Ile]** | 0.00003 |
| rs922983390 | C**A**G ⇒ C**G**G | NP_473455.5 | [715](https://www.ncbi.nlm.nih.gov/sites/protein/NP_473455.2?report=graph&v=665:765&content=5&m=715!&mn=rs922983390&dispmax=1&currpage=1) | **Q [Gln]**⇒ **R [Arg]** | 0.0001 |
| rs935154014 | **A**AC ⇒ **G**AC | NP_473455.6 | [444](https://www.ncbi.nlm.nih.gov/sites/protein/NP_473455.2?report=graph&v=394:494&content=5&m=444!&mn=rs935154014&dispmax=1&currpage=1) | **N [Asn]**⇒ **D [Asp]** | 0.00003 |
| rs943019544 | **C**CC ⇒ **A**CC | NP_473455.7 | [61](https://www.ncbi.nlm.nih.gov/sites/protein/NP_473455.2?report=graph&v=11:111&content=5&m=61!&mn=rs943019544&dispmax=1&currpage=1) | **P [Pro]**⇒ **T [Thr]** | 0.00003 |
| rs948731632 | **G**GG ⇒ **A**GG | NP_473455.8 | [278](https://www.ncbi.nlm.nih.gov/sites/protein/NP_473455.2?report=graph&v=228:328&content=5&m=278!&mn=rs948731632&dispmax=1&currpage=1) | **G [Gly]**⇒ **R [Arg]** | 0.0001 |
| rs967036625 | T**T**G ⇒ T**C**G | NP_473455.9 | [77](https://www.ncbi.nlm.nih.gov/sites/protein/NP_473455.2?report=graph&v=27:127&content=5&m=77!&mn=rs967036625&dispmax=1&currpage=1) | **L [Leu]**⇒ **S [Ser]** | 0.0002 |
| rs970379221 | A**T**T ⇒ A**C**T | NP_473455.10 | [358](https://www.ncbi.nlm.nih.gov/sites/protein/NP_473455.2?report=graph&v=308:408&content=5&m=358!&mn=rs970379221&dispmax=1&currpage=1) | **I [Ile]**⇒ **T [Thr]** | 0.00003 |
| rs976905679 | G**C**T ⇒ G**A**T | NP_473455.11 | [720](https://www.ncbi.nlm.nih.gov/sites/protein/NP_473455.2?report=graph&v=670:770&content=5&m=720!&mn=rs976905679&dispmax=1&currpage=1) | **A [Ala]**⇒ **D [Asp]** | 0.00003 |
| rs984699796 | C**G**A ⇒ C**A**A | NP_473455.12 | [502](https://www.ncbi.nlm.nih.gov/sites/protein/NP_473455.2?report=graph&v=452:552&content=5&m=502!&mn=rs984699796&dispmax=1&currpage=1) | **R [Arg]**⇒ **Q [Gln]** | 0.00003 |
| rs1005691567 | **G**AG ⇒ **A**AG | NP_473455.13 | [389](https://www.ncbi.nlm.nih.gov/sites/protein/NP_473455.2?report=graph&v=339:439&content=5&m=389!&mn=rs1005691567&dispmax=1&currpage=1) | **E [Glu]**⇒ **K [Lys]** | 0.0001 |
| rs1006817176 | **C**CG ⇒ **T**CG | NP_473455.14 | [540](https://www.ncbi.nlm.nih.gov/sites/protein/NP_473455.2?report=graph&v=490:590&content=5&m=540!&mn=rs1006817176&dispmax=1&currpage=1) | **P [Pro]**⇒ **S [Ser]** | 0.00003 |
| rs1054040936 | **G**TG ⇒ **A**TG | NP_473455.15 | [438](https://www.ncbi.nlm.nih.gov/sites/protein/NP_473455.2?report=graph&v=388:488&content=5&m=438!&mn=rs1054040936&dispmax=1&currpage=1) | **V [Val]**⇒ **M [Met]** | 0.00003 |
| rs61753626 | AGC ⇒ AAC | NP_473455.2 | [344](https://www.ncbi.nlm.nih.gov/sites/protein/NP_001265662.1?report=graph&v=231:331&content=5&m=281%21&mn=rs61753626&dispmax=1&currpage=1) | S [Ser] ⇒ N [Asn] | 0.007 |
| rs199797374 | **C**CC ⇒ **T**CC | NP_473455.3 | 573 | **P [Pro]**⇒ **S [Ser]** | no info |
| rs201454621 | **G**AA ⇒ **A**AA | NP_473455.4 | 503 | **E [Glu]**⇒ **K [Lys]** | no info |
| rs369625327 | A**C**C ⇒ A**T**C | NP_473455.5 | 614 | **T [Thr]**⇒ **I [Ile]** | no info |
| rs372364101 | T**T**G ⇒ T**C**G | NP_473455.6 | 36 | **L [Leu]**⇒ **S [Ser]** | no info |
| rs560184096 | A**G**C ⇒ A**C**C | NP_473455.7 | 612 | **S [Ser]**⇒ **T [Thr]** | no info |
| rs561705660 | GA**G** ⇒ GA**C** | NP_473455.8 | 665 | **E [Glu]**⇒ **D [Asp]** | no info |
| rs573855523 | A**G**C ⇒ A**A**C | NP_473455.9 | 462 | **S [Ser]**⇒ **N [Asn]** | no info |
| rs759336227 | **T**TC ⇒ **A**TC | NP_473455.9 | 569 | **F [Phe]**⇒ **I [Ile]** | no info |
| rs760323405 | **G**CC ⇒ **A**CC | NP_473455.9 | 446 | **A [Ala]**⇒ **T [Thr]** | no info |
| rs762848425 | **G**AG ⇒ **A**AG | NP_473455.9 | 665 | **E [Glu]**⇒ **K [Lys]** | no info |
| rs768423624 | **C**TC ⇒ **T**TC | NP_473455.9 | 242 | **L [Leu]**⇒ **F [Phe]** | no info |
| rs780953963 | **G**CC ⇒ **A**CC | NP_473455.9 | 126 | **A [Ala]**⇒ **T [Thr]** | no info |
| rs866271825 | **G**TC ⇒ **A**TC | NP_473455.9 | 213 | **V [Val]**⇒ **I [Ile]** | no info |
| rs866358245 | C**A**C ⇒ C**C**C | NP_473455.9 | 648 | **H [His]**⇒ **P [Pro]** | no info |
| rs866845381 | **G**AA ⇒ **A**AA | NP_473455.9 | 546 | **E [Glu]**⇒ **K [Lys]** | no info |
| rs866898464 | **G**AG ⇒ **A**AG | NP_473455.9 | 136 | **E [Glu]**⇒ **K [Lys]** | no info |
| rs879010658 | C**A**C ⇒ C**C**C | NP_473455.9 | 507 | **H [His]**⇒ **P [Pro]** | no info |
| rs902567706 | T**G**T ⇒ T**A**T | NP_473455.9 | 559 | **C [Cys]**⇒ **Y [Tyr]** | no info |
| rs931540102 | A**G**C ⇒ A**T**C | NP_473455.9 | 592 | **S [Ser]**⇒ **I [Ile]** | no info |
| rs934379119 | C**C**A ⇒ C**G**A | NP_473455.9 | 282 | **P [Pro]**⇒ **R [Arg]** | no info |
| rs935383311 | **G**TC ⇒ **A**TC | NP_473455.9 | 426 | **V [Val]**⇒ **I [Ile]** | no info |
| rs935456871 | CA**G** ⇒ CA**C** | NP_473455.9 | 305 | **Q [Gln]**⇒ **H [His]** | no info |
| rs944281508 | **C**TG ⇒ **A**TG | NP_473455.9 | 455 | **L [Leu]**⇒ **M [Met]** | no info |
| rs953953119 | G**A**G ⇒ G**G**G | NP_473455.9 | 147 | **E [Glu]**⇒ **G [Gly]** | no info |
| rs973151982 | A**A**G ⇒ A**G**G | NP_473455.9 | 494 | **K [Lys]**⇒ **R [Arg]** | no info |
| rs981870176 | **G**TG ⇒ **A**TG | NP_473455.9 | 519 | **V [Val]**⇒ **M [Met]** | no info |
| rs990556535 | **A**TG ⇒ **T**TG | NP_473455.9 | 64 | **M [Met]**⇒ **L [Leu]** | no info |
| rs999358627 | G**C**A ⇒ G**T**A | NP_473455.9 | 70 | **A [Ala]**⇒ **V [Val]** | no info |
| rs999647038 | A**A**G ⇒ A**G**G | NP_473455.9 | 545 | **K [Lys]**⇒ **R [Arg]** | no info |
| rs1012223670 | **G**TG ⇒ **C**TG | NP_473455.9 | 406 | **V [Val]**⇒ **L [Leu]** | no info |
| rs1014706694 | **C**TG ⇒ **A**TG | NP_473455.9 | 365 | **L [Leu]**⇒ **M [Met]** | no info |
| rs1031210695 | C**T**A ⇒ C**C**A | NP_473455.9 | 13 | **L [Leu]**⇒ **P [Pro]** | no info |
| rs1011620196 | G**T**G ⇒ G**C**G | NP_473455.9 | 262 | **V [Val]**⇒ **A [Ala]** | no info |
| rs778745747 | G**C**G ⇒ G**A**G | NP_473455.9 | [625](https://www.ncbi.nlm.nih.gov/sites/protein/NP_473455.2?report=graph&v=575:675&content=5&m=625!&mn=rs778745747&dispmax=1&currpage=1) | **A [Ala]**⇒ **E [Glu]** | 0.000008 |
| rs879182307 | C**T**G ⇒ C**G**G | NP_473455.9 | 520 | **L [Leu]**⇒ **R [Arg]** | no info |
| rs754842183 | **T**TC ⇒ **C**TC | NP_473455.9 | 268 | **F [Phe]**⇒ **L [Leu]** | 0.000008 |
| rs992444254 | G**C**T ⇒ G**T**T | [NP_620165.1](https://www.ncbi.nlm.nih.gov/entrez/query.fcgi?cmd=Search&db=protein&term=NP_620165.1) | 265 | **A [Ala]**⇒ **V [Val]** | no info |
| rs386443412 | merged into rs61753626 | | | | 0.0066 |
