# Supplementary material for: Identification and in silico analysis of functional SNPs of human TAGAP protein: A comprehensive study
Source: PLoS One. 2018 Jan 12;13(1):e0188143. doi: 10.1371/journal.pone.0188143 (PMC5766082; doi:10.1371/journal.pone.0188143)
Supplement: S2 Table — (DOCX) [file pone.0188143.s003.docx]

| **The results for all 275 nsSNPs by five *in silico* tools.** | **SIFT** | | **PROVEAN** | | **Polyphen2 (HumDiv)** | | **PhD-SNP** | | | **SNPs&GO** | | |
| --- | --- | --- | --- | --- | --- | --- | --- | --- | --- | --- | --- | --- |
| **Amino acid Change** | **Prediction** | **Tolerance index** | **Score** | **Prediction (cutoff= -2.5)** | **Effect** | **Score** | **Prediction** | **RI** | **Probability** | **Prediction** | **RI** | **Probability** |
| G346D | Intolerated | 0 | -1.126 | Neutral | Benign |  | Disease | 3 | 0.651 | Neutral | 5 | 0.259 |
| E147K | tolerated | 1 | -1.507 | Neutral | Benign |  | Disease | 1 | 0.545 | Neutral | 7 | 0.142 |
| T396S | Intolerated | 0.02 | 0.482 | Neutral | Benign |  | Neutral |  |  | Neutral |  |  |
| P347T | tolerated | 0.24 | -0.935 | Neutral | Benign |  | Neutral |  |  | Neutral |  |  |
| H284Y | tolerated | 0.17 | -1.368 | Neutral | Possibly damaging | 0.867 | Neutral |  |  | Neutral |  |  |
| G542D | tolerated | 0.96 | 0.073 | Neutral | Benign |  | Neutral |  |  | Neutral |  |  |
| P428S | tolerated | 0.46 | -2.625 | Deleterious | Benign |  | Neutral |  |  | Neutral |  |  |
| A345T | tolerated | 0.34 | 0.055 | Neutral | Benign |  | Neutral |  |  | Neutral |  |  |
| E192K | Intolerated | 0.02 | -1.502 | Neutral | Possibly damaging | 0.935 | Neutral |  |  | Neutral |  |  |
| C632G | tolerated | 0.16 | -0.023 | Neutral | Benign |  | Disease | 3 | 0.639 | Neutral | 5 | 0.264 |
| K52T | Intolerated | 0 | -3.401 | Deleterious | Probably damaging | 0.999 | Disease | 1 | 0.57 | Neutral | 8 | 0.112 |
| S44R | Tolerated | 0.24 | -1.427 | Neutral | Benign |  | Neutral |  |  | Neutral |  |  |
| A604V | tolerated | 1 | -1.132 | Neutral | Benign |  | Neutral |  |  | Neutral |  |  |
| E187D | tolerated | 0.09 | -0.66 | Neutral | Benign |  | Neutral |  |  | Neutral |  |  |
| G410A | tolerated | 1 | -0.504 | Neutral | Benign |  | Neutral |  |  | Neutral |  |  |
| T118A | Intolerated | 0.05 | -4.644 | Deleterious | Probably damaging | 1 | Disease | 1 | 0.571 | Neutral | 5 | 0.249 |
| K518T | Intolerated | 0.01 | -2.831 | Deleterious | Benign |  | Disease | 6 | 0.785 | Neutral | 1 | 0.435 |
| Y725C | tolerated | 0.71 | -3.981 | Deleterious | Benign |  | Disease | 1 | 0.558 | Neutral | 4 | 0.278 |
| V359G | tolerated | 0.13 | -1.596 | Neutral | Possibly damaging | 0.483 | Disease | 3 | 0.657 | Neutral | 7 | 0.13 |
| R706Q | tolerated | 0.06 | 0.809 | Neutral | Benign |  | Neutral | 6 | 0.186 | Neutral | 9 | 0.068 |
| V554M | Intolerated | 0.02 | -1.247 | Neutral | Probably damaging | 0.993 | Disease | 3 | 0.647 | Neutral | 7 | 0.174 |
| P315A | Intolerated | 0 | -4.301 | Deleterious | Probably damaging | 0.976 | Neutral |  |  | Neutral |  |  |
| V215A | Intolerated | 0.01 | -3.643 | Deleterious | Possibly damaging | 0.891 | Neutral |  |  | Neutral |  |  |
| A350D | tolerated | 0.08 | -0.209 | Neutral | Possibly damaging | 0.454 | Neutral |  |  | Neutral |  |  |
| H674R | tolerated | 0.68 | -0.013 | Neutral | Benign |  | Neutral |  |  | Neutral |  |  |
| E588K | tolerated | 0.44 | 0.999 | Neutral | Benign |  | Neutral | 3 | 0.368 | Disease | 0 | 0.515 |
| R131H | tolerated | 0.22 | -2.439 | Neutral | Probably damaging | 1 | Neutral |  |  | Neutral |  |  |
| A459P | tolerated | 0.17 | -1.928 | Neutral | Probably damaging | 1 | Disease | 6 | 0.775 | Neutral | 3 | 0.333 |
| L81S | Tolerated | 0.22 | -1.195 | Neutral | Possibly damaging | 0.675 | Disease | 0 | 0.506 | Neutral | 7 | 0.139 |
| S26P | Tolerated | 0.12 | -2.382 | Neutral | Benign |  | Disease | 4 | 0.723 | Neutral | 4 | 0.295 |
| S411C | Intolerated | 0.04 | -0.858 | Neutral | Benign |  | Neutral |  |  | Neutral |  |  |
| V673A | tolerated | 0.62 | -0.397 | Neutral | Benign |  | Neutral |  |  | Neutral |  |  |
| P636A | Intolerated | 0 | -1.563 | Neutral | Benign |  | Neutral |  |  | Neutral |  |  |
| F158L | tolerated | 1 | 0.038 | Neutral | Benign |  | Neutral |  |  | Neutral |  |  |
| E187K | tolerated | 0.42 | -1.802 | Neutral | Possibly damaging | 0.87 | Neutral |  |  | Neutral |  |  |
| V362M | Intolerated | 0.04 | -1.055 | Neutral | Possibly damaging | 0.918 | Neutral |  |  | Neutral |  |  |
| R167Q | tolerated | 0.39 | 0.253 | Neutral | Benign |  | Neutral |  |  | Neutral |  |  |
| L709P | Intolerated | 0.01 | -2.815 | Deleterious | Probably damaging | 0.996 | Disease | 8 | 0.9 | Disease | 1 | 0.561 |
| P103A | Intolerated | 0.04 | -7.31 | Deleterious | Probably damaging | 1 | Neutral |  |  | Neutral |  |  |
| T118M | Intolerated | 0 | -5.67 | Deleterious | Probably damaging | 1 | Disease | 4 | 0.678 | Neutral | 4 | 0.29 |
| S479G | Intolerated | 0.04 | -1.573 | Neutral | Possibly damaging | 0.89 | Neutral |  |  | Neutral |  |  |
| D257N | tolerated | 0.17 | -1.927 | Neutral | Benign |  | Neutral |  |  | Neutral |  |  |
| Q583R | Intolerated | 0 | -1.506 | Neutral | Benign |  | Neutral |  |  | Neutral |  |  |
| I92T | tolerated | 0.11 | 0.471 | Neutral | Benign |  | Neutral |  |  | Neutral |  |  |
| V643I | tolerated | 0.34 | -0.337 | Neutral | Benign |  | Neutral |  |  | Neutral |  |  |
| E23K | Intolerated | 0 | -2.235 | Neutral | Possibly damaging | 0.647 | Disease | 6 | 0.815 | Neutral | 2 | 0.404 |
| D173N | Intolerated | 0.04 | -3.194 | Deleterious | Probably damaging | 0.967 | Neutral |  |  | Neutral |  |  |
| A15T | Tolerated | 0.13 | -1.14 | Neutral | Benign |  | Disease | 5 | 0.748 | Neutral | 5 | 0.263 |
| K82N | Intolerated | 0 | -2.944 | Deleterious | Probably damaging | 0.998 | Neutral |  |  | Neutral |  |  |
| H152Y | Intolerated | 0 | -1.22 | Neutral | Probably damaging | 0.999 | Disease | 7 | 0.849 | Disease | 0 | 0.521 |
| A630V | tolerated | 0.28 | -0.548 | Neutral | Benign |  | Neutral |  |  | Neutral |  |  |
| H648Y | Intolerated | 0 | -0.613 | Neutral | Possibly damaging | 0.539 | Neutral |  |  | Neutral |  |  |
| T672S | Intolerated | 0 | -0.891 | Neutral | Possibly damaging | 0.58 | Neutral |  |  | Neutral |  |  |
| V608E | tolerated | 0.74 | -0.237 | Neutral | Benign |  | Disease | 4 | 0.683 | Neutral | 4 | 0.322 |
| N8S | Intolerated | 0.04 | -1.222 | Neutral | Benign |  | Disease | 1 | 0.526 | Neutral | 7 | 0.136 |
| Y725H | Intolerated | 0.02 | -2.615 | Deleterious | Probably damaging | 0.999 | Disease | 4 | 0.69 | Neutral | 4 | 0.312 |
| I270T | Intolerated | 0 | -4.249 | Deleterious | Probably damaging | 0.968 | Disease | 8 | 0.888 | Neutral | 3 | 0.347 |
| S5R | Intolerated | 0 | -3.256 | Deleterious | Probably damaging | 1 | Neutral | 1 | 0.474 | Neutral | 7 | 0.143 |
| N563S | tolerated | 0.92 | 0.377 | Neutral | Benign |  | Neutral |  |  | Neutral |  |  |
| R131C | Intolerated | 0 | 0.307 | Neutral | Probably damaging | 1 | Neutral |  |  | Neutral |  |  |
| S469C | tolerated | 0.13 | -2.161 | Neutral | Probably damaging | 0.993 | Neutral |  |  | Neutral |  |  |
| G659R | tolerated | 0.17 | 0.352 | Neutral | Benign |  | Neutral |  |  | Neutral |  |  |
| V266M | Tolerated | 0.1 | -2.273 | Neutral | Probably damaging | 0.963 | Disease | 4 | 0.718 | Neutral | 4 | 0.287 |
| P636H | Intolerated | 0.01 | -2.198 | Neutral | Probably damaging | 0.983 | Disease | 2 | 0.596 | Neutral | 8 | 0.117 |
| R351Q | tolerated | 0.29 | 0.087 | Neutral | Benign |  | Neutral |  |  | Neutral |  |  |
| Q583K | tolerated | 0.41 | -1.42 | Neutral | Benign |  | Disease | 4 | 0.687 | Neutral | 2 | 0.417 |
| A9G | Tolerated | 0.34 | -2.002 | Neutral | Benign |  | Neutral | 5 | 0.268 | Neutral | 9 | 0.067 |
| M18V | Intolerated | 0 | -2.593 | Deleterious | Probably damaging | 0.997 | Neutral |  |  | Neutral |  |  |
| P315L | Intolerated | 0.01 | -5.397 | Deleterious | Probably damaging | 0.999 | Neutral |  |  | Neutral |  |  |
| K114E | Intolerated | 0.01 | -2.136 | Neutral | Probably damaging | 0.987 | Disease | 3 | 0.636 | Neutral | 4 | 0.297 |
| S464L | Intolerated | 0.01 | -3.838 | Deleterious | Probably damaging | 0.998 | Neutral |  |  | Neutral |  |  |
| P480S | Tolerated | 0.08 | -2.806 | Deleterious | Benign |  | Neutral |  |  | Neutral |  |  |
| D297G | Intolerated | 0.02 | -4.586 | Deleterious | Probably damaging | 0.997 | Neutral |  |  | Neutral |  |  |
| L194M | tolerated | 0.14 | -1.173 | Neutral | Probably damaging | 0.994 | Neutral |  |  | Neutral |  |  |
| K668T | tolerated | 0.73 | -0.115 | Neutral | Benign |  | Neutral |  |  | Neutral |  |  |
| S577L | tolerated | 0.09 | -4.36 | Deleterious | Benign |  | Disease | 7 | 0.868 | Neutral | 3 | 0.371 |
| A143V | tolerated | 0.35 | -0.466 | Neutral | Benign |  | Neutral |  |  | Neutral |  |  |
| G346S | tolerated | 0.56 | -0.413 | Neutral | Benign |  | Neutral |  |  | Neutral |  |  |
| F582L | Intolerated | 0.02 | -3.281 | Deleterious | Probably damaging | 1 | Disease | 5 | 0.766 | Neutral | 9 | 0.053 |
| D537N | tolerated | 0.06 | -1.709 | Neutral | Possibly damaging | 0.911 | Neutral |  |  | Neutral |  |  |
| D373N | Intolerated | 0.01 | -1.641 | Neutral | Probably damaging | 0.992 | Neutral |  |  | Neutral |  |  |
| P716Q | Intolerated | 0.02 | -5.043 | Deleterious | Probably damaging | 1 | Disease | 7 | 0.84 | Neutral | 1 | 0.428 |
| Q702R | Intolerated | 0.03 | -1.914 | Neutral | Possibly damaging | 0.949 | Disease | 2 | 0.603 | Neutral | 7 | 0.144 |
| R148S | tolerated | 1 | 1.634 | Neutral | Benign |  | Neutral |  |  | Neutral |  |  |
| R408W | Intolerated | 0 | -3.024 | Deleterious | Probably damaging | 0.996 | Disease | 3 | 0.648 | Neutral | 4 | 0.318 |
| D314N | Intolerated | 0.03 | -3.158 | Deleterious | Probably damaging | 1 | Neutral |  |  | Neutral |  |  |
| H488Y | Intolerated | 0 | -3.005 | Deleterious | Probably damaging | 0.999 | Disease | 5 | 0.739 | Neutral | 2 | 0.377 |
| R453Q | tolerated | 0.65 | -0.244 | Neutral | Benign |  | Neutral |  |  | Neutral |  |  |
| L100F | Intolerated | 0 | -3.769 | Deleterious | Probably damaging | 1 | Disease | 2 | 0.594 | Neutral | 7 | 0.166 |
| Y376H | tolerated | 0.1 | -1.679 | Neutral | Benign |  | Neutral |  |  | Neutral |  |  |
| A526G | tolerated | 0.07 | -1.327 | Neutral | Benign |  | Neutral |  |  | Neutral |  |  |
| F491C | Tolerated | 0.17 | -3.492 | Deleterious | Possibly damaging | 0.799 | Disease | 4 | 0.698 | Neutral | 4 | 0.303 |
| G602C | Tolerated | 0.19 | 0.898 | Neutral | Benign |  | Neutral |  |  | Neutral |  |  |
| G498D | tolerated | 0.07 | -0.604 | Neutral | Benign |  | Neutral |  |  | Neutral |  |  |
| Q333L | tolerated | 0.22 | -1.432 | Neutral | Benign |  | Neutral |  |  | Neutral |  |  |
| S84T | tolerated | 0.82 | -1.076 | Neutral | Benign |  | Neutral |  |  | Neutral |  |  |
| S501R | tolerated | 0.23 | -1.101 | Neutral | Benign |  | Neutral |  |  | Neutral |  |  |
| V710M | tolerated | 0.05 | -0.076 | Neutral | Benign |  | Neutral | 5 | 0.247 | Neutral | 9 | 0.065 |
| S10P | Tolerated | 0.26 | -1.007 | Neutral | Benign |  | Disease | 6 | 0.801 | Neutral | 4 | 0.281 |
| V157I | Tolerated | 0.08 | -0.546 | Neutral | Benign |  | Neutral |  |  | Neutral |  |  |
| I110V | Tolerated | 0.05 | -0.776 | Neutral | Probably damaging | 0.958 | Neutral |  |  | Neutral |  |  |
| V450M | Tolerated | 0.06 | -0.42 | Neutral | Possibly damaging | 0.744 | Disease | 2 | 0.614 | Neutral | 8 | 0.119 |
| F268L | Intolerated | 0 | -5.912 | Deleterious | Probably damaging | 1 | Neutral |  |  | Neutral |  |  |
| E597K | Intolerated | 0 | -2.752 | Deleterious | Possibly damaging | 0.802 | Disease | 3 | 0.633 | Neutral | 6 | 0.207 |
| R167W | Tolerated | 0.08 | -0.901 | Neutral | Probably damaging | 0.994 | Neutral |  |  | Neutral |  |  |
| H642Y | tolerated | 0.98 | -1.128 | Neutral | Benign |  | Neutral |  |  | Neutral |  |  |
| R552Q | Intolerated | 0 | -1.373 | Neutral | Probably damaging | 1 | Disease | 7 | 0.839 | Neutral | 4 | 0.299 |
| P69A | Tolerated | 0.36 | -0.851 | Neutral | Benign |  | Neutral |  |  | Neutral |  |  |
| P421T | Tolerated | 0.63 | -1.414 | Neutral | Possibly damaging | 0.894 | Neutral |  |  | Neutral |  |  |
| A599T | Intolerated | 0.01 | -2.398 | Neutral | Probably damaging | 0.994 | Disease | 3 | 0.627 | Neutral | 7 | 0.164 |
| H631D | tolerated | 1 | 1.989 | Neutral | Benign |  | Neutral |  |  | Neutral |  |  |
| R706W | tolerated | 0.16 | -2.04 | Neutral | Benign |  | Disease | 0 | 0.513 | Neutral | 6 | 0.188 |
| P332S | tolerated | 0.35 | -0.598 | Neutral | Benign |  | Neutral |  |  | Neutral |  |  |
| S670N | tolerated | 0.38 | -0.591 | Neutral | Benign |  | Neutral |  |  | Neutral |  |  |
| C708R | tolerated | 0.34 | -1.459 | Neutral | Benign |  | Disease | 1 | 0.554 | Neutral | 6 | 0.202 |
| V701L | tolerated | 0.68 | -0.4 | Neutral | Benign |  | Neutral | 2 | 0.425 | Neutral |  |  |
| P335L | Intolerated | 0.04 | -1.908 | Neutral | Benign |  | Neutral |  |  | Neutral |  |  |
| T672I | Intolerated | 0 | -1.128 | Neutral | Possibly damaging | 0.906 | Disease | 2 | 0.595 | Neutral | 7 | 0.161 |
| R541S | Tolerated | 0.17 | -0.825 | Neutral | Benign |  | Neutral |  |  | Neutral |  |  |
| D29N | Tolerated | 0.23 | -1.421 | Neutral | Benign |  | Neutral |  |  | Neutral |  |  |
| A339D | Intolerated | 0.02 | -1.074 | Neutral | Possibly damaging | 0.868 | Disease | 2 | 0.593 | Neutral | 4 | 0.277 |
| R412H | tolerated | 0.57 | -0.129 | Neutral | Benign |  | Neutral |  |  | Neutral |  |  |
| L388R | Intolerated | 0.01 | -2.153 | Neutral | Possibly damaging | 0.933 | Disease | 4 | 0.681 | Neutral | 8 | 0.125 |
| S479N | Intolerated | 0.03 | -1.533 | Neutral | Probably damaging | 0.989 | Neutral |  |  | Neutral |  |  |
| P515R | Intolerated | 0.02 | -0.894 | Neutral | Benign |  | Neutral |  |  | Neutral |  |  |
| R712Q | tolerated | 1 | -2.505 | Deleterious | Possibly damaging | 0.887 | Disease | 3 | 0.66 | Neutral | 5 | 0.259 |
| P166L | Intolerated | 0.01 | -9.589 | Deleterious | Probably damaging | 1 | Disease | 7 | 0.828 | Neutral | 8 | 0.097 |
| A363T | tolerated | 1 | -0.138 | Neutral | Benign |  | Neutral |  |  | Neutral |  |  |
| T296S | tolerated | 0.07 | -1.118 | Neutral | Possibly damaging | 0.675 | Neutral |  |  | Neutral |  |  |
| H562Q | tolerated | 0.45 | -1.082 | Neutral | Benign |  | Neutral |  |  | Neutral |  |  |
| N523S | tolerated | 0.15 | 0.561 | Neutral | Benign |  | Neutral |  |  | Neutral |  |  |
| V430G | tolerated | 0.38 | -0.592 | Neutral | Benign |  | Disease | 3 | 0.63 | Neutral | 7 | 0.161 |
| A339P | tolerated | 0.29 | -1.577 | Neutral | Possibly damaging | 0.93 | Disease | 2 | 0.609 | Neutral | 4 | 0.321 |
| S308P | Intolerated | 0 | -4.827 | Deleterious | Probably damaging | 1 | Neutral |  |  | Neutral |  |  |
| D96N | Intolerated | 0.01 | -2.276 | Neutral | Possibly damaging | 0.799 | Disease | 1 | 0.525 | Neutral | 7 | 0.164 |
| H284R | tolerated | 0.87 | -1.799 | Neutral | Benign |  | Neutral |  |  | Neutral |  |  |
| I442V | tolerated | 0.66 | -0.167 | Neutral | Benign |  | Neutral |  |  | Neutral |  |  |
| P447S | tolerated | 0.62 | 1.068 | Neutral | Benign |  | Neutral |  |  | Neutral |  |  |
| C94R | Intolerated | 0 | -11.107 | Deleterious | Probably damaging | 1 | Disease | 7 | 0.857 | Neutral | 1 | 0.44 |
| S302L | Intolerated | 0.03 | -3.893 | Deleterious | Probably damaging | 0.979 | Neutral |  |  | Neutral |  |  |
| N14S | Intolerated | 0 | -1.82 | Neutral | Possibly damaging | 0.647 | Disease | 3 | 0.655 | Neutral | 5 | 0.235 |
| F511S | Intolerated | 0 | -3.352 | Deleterious | Probably damaging | 1 | Disease | 7 | 0.874 | Neutral | 2 | 0.389 |
| V474M | Tolerated | 0.19 | -0.043 | Neutral | Benign |  | Neutral |  |  | Neutral |  |  |
| R688I | Intolerated | 0.04 | -3.089 | Deleterious | Possibly damaging | 0.868 | Disease | 1 | 0.527 | Neutral | 7 | 0.142 |
| F122L | Intolerated | 0 | -5.753 | Deleterious | Probably damaging | 1 | Disease | 7 | 0.848 | Disease | 1 | 0.534 |
| C570R | Tolerated | 0.56 | 0.445 | Neutral | Benign |  | Disease | 4 | 0.711 | Neutral | 4 | 0.301 |
| S510F | Intolerated | 0.01 | -4.205 | Deleterious | Probably damaging | 0.999 | Disease | 5 | 0.757 | Neutral | 2 | 0.399 |
| P357T | Intolerated | 0.04 | -1.825 | Neutral | Probably damaging | 0.972 | Neutral |  |  | Neutral |  |  |
| G28V | Intolerated | 0.02 | -3.423 | Deleterious | Possibly damaging | 0.802 | Disease | 3 | 0.633 | Neutral | 5 | 0.232 |
| G687A | tolerated | 0.98 | -0.605 | Neutral | Benign |  | Neutral |  |  | Neutral |  |  |
| R567H | Intolerated | 0.03 | -1.085 | Neutral | Possibly damaging | 0.893 | Neutral |  |  | Neutral |  |  |
| G120E | Intolerated | 0 | -7.671 | Deleterious | Probably damaging | 1 | Disease | 8 | 0.925 | Disease | 4 | 0.708 |
| R227T | Intolerated | 0.01 | -2.818 | Deleterious | Benign |  | Disease | 1 | 0.527 | Neutral | 8 | 0.103 |
| E32A | Tolearted | 0.62 | -1.671 | Neutral | Benign |  | Neutral |  |  | Neutral |  |  |
| N260K | Intolerated | 0.04 | -0.326 | Neutral | Benign |  | Neutral |  |  | Neutral |  |  |
| N205S | Intolerated | 0 | -4.878 | Deleterious | Probably damaging | 1 | Disease | 5 | 0.775 | Disease | 3 | 0.654 |
| E414D | tolerated | 0.41 | -1.427 | Neutral | Benign |  | Neutral |  |  | Neutral |  |  |
| F161V | Intolerated | 0.02 | -6.712 | Deleterious | Probably damaging | 0.999 | Disease | 9 | 0.943 | Disease | 8 | 0.881 |
| E556K | Intolerated | 0.01 | -1.674 | Neutral | Probably damaging | 0.995 | Disease | 8 | 0.882 | Neutral | 0 | 0.492 |
| T398P | Intolerated | 0.01 | -3.072 | Deleterious | Probably damaging | 0.966 | Disease | 4 | 0.683 | Neutral | 8 | 0.088 |
| D43G | Intolerated | 0.01 | -4.146 | Deleterious | Probably damaging | 0.993 | Disease | 5 | 0.727 | Neutral | 5 | 0.237 |
| M336R | Tolerated | 0.54 | -0.698 | Neutral | Benign |  | Neutral |  |  | Neutral |  |  |
| Q305P | Intolerated | 0 | -4.319 | Deleterious | Probably damaging | 0.989 | Disease | 1 | 0.538 | Neutral | 8 | 0.112 |
| P603A | Tolerated | 0.41 | -0.06 | Neutral | Benign |  | Neutral |  |  | Neutral |  |  |
| A83V | tolerated | 1 | -1.285 | Neutral | Benign |  | Neutral |  |  | Neutral |  |  |
| G681R | tolerated | 0.14 | -0.984 | Neutral | Benign |  | Disease | 1 | 0.566 | Neutral | 7 | 0.143 |
| D199N | tolerated | 0.25 | -2.367 | Neutral | Benign |  | Neutral |  |  | Neutral |  |  |
| D707N | Intolerated | 0.02 | -2.631 | Deleterious | Probably damaging | 0.993 | Neutral | 1 | 0.454 | Neutral | 8 | 0.082 |
| G657S | tolerated | 0.5 | -0.46 | Neutral | Benign |  | Neutral |  |  | Neutral |  |  |
| L342F | tolerated | 0.35 | -0.53 | Neutral | Possibly damaging | 0.498 | Neutral |  |  | Neutral |  |  |
| S490P | Intolerated | 0 | -2.6 | Deleterious | Probably damaging | 1 | Disease | 1 | 0.575 | Disease | 2 | 0.595 |
| A526T | tolerated | 0.42 | 0.094 | Neutral | Benign |  | Neutral |  |  | Neutral |  |  |
| S344R | tolerated | 0.07 | -0.769 | Neutral | Benign |  | Neutral |  |  | Neutral |  |  |
| R4K | Tolerated | 0.07 | 0.209 | Neutral | Benign |  | Neutral | 8 | 0.09 | Neutral | 9 | 0.042 |
| H574N | tolerated | 0.31 | -1.835 | Neutral | Benign |  | Disease | 3 | 0.628 | Neutral | 7 | 0.156 |
| S698F | Intolerated | 0.01 | -3.737 | Deleterious | Probably damaging | 1 | Disease | 1 | 0.564 | Neutral | 7 | 0.151 |
| C713R | tolerated | 0.29 | -7.542 | Deleterious | Benign |  | Disease | 8 | 0.904 | Disease | 3 | 0.647 |
| G141A | tolerated | 0.05 | -5.753 | Deleterious | Probably damaging | 1 | Disease | 0 | 0.508 | Neutral | 5 | 0.231 |
| G95S | tolerated | 0.28 | -0.45 | Neutral | Benign |  | Neutral |  |  | Neutral |  |  |
| V57M | Intolerated | 0.01 | -1.626 | Neutral | Probably damaging | 0.998 | Neutral |  |  | Neutral |  |  |
| D311N | Intolerated | 0.02 | -4.194 | Deleterious | Probably damaging | 1 | Neutral |  |  | Neutral |  |  |
| L67H | Intolerated | 0 | -1.418 | Neutral | Possibly damaging | 0.855 | Disease | 1 | 0.566 | Neutral | 7 | 0.147 |
| N483H | tolerated | 0.14 | -2.343 | Neutral | Benign |  | Neutral |  |  | Neutral |  |  |
| S220G | Intolerated | 0 | -3.036 | Deleterious | Possibly damaging | 0.923 | Disease | 0 | 0.501 | Neutral | 7 | 0.125 |
| A370T | Tolerated | 0.24 | 0.192 | Neutral | Benign |  | Neutral |  |  | Neutral |  |  |
| T619M | Intolerated | 0.01 | -2.997 | Deleterious | Probably damaging | 1 | Disease | 4 | 0.722 | Neutral | 4 | 0.306 |
| D645G | Intolerated | 0 | -1.077 | Neutral | Benign |  | Neutral |  |  | Neutral |  |  |
| D98N | tolerated | 0.14 | -2.071 | Neutral | Benign |  | Neutral |  |  | Neutral |  |  |
| V409A | Tolerated | 1 | 1.338 | Neutral | Benign |  | Neutral |  |  | Neutral |  |  |
| T288S | tolerated | 0.68 | -0.492 | Neutral | Benign |  | Neutral |  |  | Neutral |  |  |
| D186N | tolerated | 0.54 | 1.269 | Neutral | Benign |  | Neutral |  |  | Neutral |  |  |
| D314E | Intolerated | 0 | -1.858 | Neutral | Probably damaging | 0.999 | Neutral |  |  | Neutral |  |  |
| P656S | Tolerated | 0.58 | 0.394 | Neutral | Benign |  | Neutral |  |  | Neutral |  |  |
| S476F | Intolerated | 0 | -4.967 | Deleterious | Probably damaging | 1 | Disease | 1 | 0.535 | Neutral | 8 | 0.098 |
| S360R | Intolerated | 0 | -2.398 | Neutral | Probably damaging | 0.986 | Neutral |  |  | Neutral |  |  |
| R711Q | Tolerated | 0.1 | -1.644 | Neutral | Benign |  | Disease | 3 | 0.657 | Neutral | 5 | 0.259 |
| R695S | Tolerated | 0.12 | -2.878 | Deleterious | Possibly damaging | 0.921 | Disease | 5 | 0.758 | Neutral | 3 | 0.343 |
| S367I | Intolerated | 0.02 | -2.606 | Deleterious | Possibly damaging | 0.766 | Disease | 4 | 0.7 | Disease | 1 | 0.558 |
| A37V | Tolearted | 0.14 | -1.758 | Neutral | Benign |  | Neutral |  |  | Neutral |  |  |
| G141W | Intolerated | 0 | -7.671 | Deleterious | Probably damaging | 1 | Disease | 6 | 0.807 | Disease | 0 | 0.503 |
| V334M | tolerated | 0.13 | -0.262 | Neutral | Benign |  | Neutral |  |  | Neutral |  |  |
| Q613H | Tolerated | 0.19 | -0.733 | Neutral | Probably damaging | 0.989 | Neutral |  |  | Neutral |  |  |
| E699K | Intolerated | 0 | -2.517 | Deleterious | Probably damaging | 0.999 | Disease | 7 | 0.857 | Neutral | 1 | 0.443 |
| V539I | Tolerated | 0.46 | -0.188 | Neutral | Possibly damaging | 0.701 | Neutral |  |  | Neutral |  |  |
| D247N | Intolerated | 0.05 | -2.22 | Neutral | Probably damaging | 0.968 | Neutral |  |  | Neutral |  |  |
| L67I | tolearated | 0.18 | -0.536 | Neutral | Benign |  | Neutral |  |  | Neutral |  |  |
| V151M | Intolerated | 0 | -2.596 | Deleterious | Probably damaging | 1 | Disease | 2 | 0.585 | Neutral | 3 | 0.338 |
| F718S | Tolerated | 0.07 | -5.07 | Deleterious | Probably damaging | 1 | Disease | 8 | 0.909 | Disease | 3 | 0.638 |
| D349E | Tolerated | 1 | -0.501 | Neutral | Benign |  | Neutral |  |  | Neutral |  |  |
| H488Q | Tolerated | 0.06 | -3.761 | Deleterious | Possibly damaging | 0.787 | Disease | 3 | 0.672 | Neutral | 7 | 0.17 |
| L455P | tolerated | 0.39 | -0.597 | Neutral | Benign |  | Disease | 1 | 0.561 | Disease | 1 | 0.543 |
| A76T | tolerated | 0.1 | -0.729 | Neutral | Benign |  | Neutral |  |  | Neutral |  |  |
| I325T | tolerated | 0.92 | -0.288 | Neutral | Benign |  | Neutral |  |  | Neutral |  |  |
| T495I | Intolerated | 0.01 | -2.37 | Neutral | Benign |  | Neutral |  |  | Neutral |  |  |
| L663V | Tolerated | 0.61 | 0.36 | Neutral | Benign |  | Neutral |  |  | Neutral |  |  |
| R703S | Intolerated | 0.01 | -1.145 | Neutral | Benign |  | Disease | 4 | 0.709 | Neutral | 4 | 0.302 |
| V225M | Intolerated | 0.02 | -1.338 | Neutral | Possibly damaging | 0.845 | Neutral |  |  | Neutral |  |  |
| S172N | tolerated | 0.19 | -2.613 | Deleterious | Possibly damaging | 0.693 | Neutral |  |  | Neutral |  |  |
| M228K | Intolerated | 0 | -5.817 | Deleterious | Probably damaging | 0.983 | Disease | 9 | 0.949 | Disease | 5 | 0.767 |
| T535I | Tolerated | 0.06 | -1.585 | Neutral | Benign |  | Neutral |  |  | Neutral |  |  |
| S329N | Tolerated | 1 | -0.326 | Neutral | Benign |  | Neutral |  |  | Neutral |  |  |
| S380T | Tolerated | 0.17 | -0.933 | Neutral | Possibly damaging | 0.651 | Neutral |  |  | Neutral |  |  |
| D160E | Tolerated | 1 | -3.169 | Deleterious | Possibly damaging | 0.910 | Neutral | 5 |  | Neutral | 9 |  |
| M627I | Intolerated | 0 | -1.436 | Neutral | Benign | 0.024 | Neutral | 7 |  | Neutral | 4 |  |
| Q715R | Intolerated | 0.03 | -2.745 | Deleterious | Probably damaging | 0.986 | Neutral | 3 |  | Neutral | 3 |  |
| N444D | Intolerated | 0 | -1.382 | Neutral | Benign | 0.003 | Neutral | 6 |  | Neutral | 8 |  |
| P61T | Tolerated | 0.15 | -2.214 | Neutral | Probably damaging | 0.993 | Neutral | 8 |  | Neutral | 9 |  |
| G278R | Intolerated | 0.02 | -7.105 | Deleterious | Probably damaging | 1 | Neutral | 8 |  | Disease | 4 | 0.711 |
| L77S | Tolerated | 0.3 | -0.117 | Neutral | Benign | 0.01 | Neutral | 5 |  | Neutral | 8 |  |
| I358T | Tolerated | 0.69 | -0.739 | Neutral | Possibly damaging | 0.483 | Neutral | 7 |  | Neutral | 9 |  |
| A720D | Intolerated | 0 | -1.972 | Neutral | Benign | 0.144 | Neutral | 2 |  | Disease | 0 | 0.515 |
| R502Q | Intolerated | 0 | -1.464 | Neutral | Probably damaging | 1 | Neutral | 5 |  | Neutral | 5 |  |
| E389K | Tolerated | 0.24 | -1.076 | Neutral | Benign | 0.246 | Neutral | 7 |  | Neutral | 9 |  |
| P540S | Intolerated | 0.05 | -0.172 | Neutral | Benign | 0.009 | Neutral | 9 |  | Neutral | 9 |  |
| V438M | Intolerated | 0 | 0.29 | Neutral | Benign | 0.081 | Neutral | 8 |  | Neutral | 8 |  |
| S344N | Intolerated | 0.02 | -0.28 | Neutral | Benign | 0.001 | Neutral | 8 |  | Neutral | 8 |  |
| P573S | tolerated | 1 | -0.478 | Neutral | Benign |  | Neutral |  |  | Neutral |  |  |
| E503K | Intolerated | 0 | -2.834 | Deleterious | Probably damaging | 0.986 | Disease | 5 | 0.763 | Neutral | 2 | 0.38 |
| T614I | tolerated | 0.06 | -0.961 | Neutral | Possibly damaging | 0.531 | Disease | 2 | 0.589 | Neutral | 5 | 0.227 |
| L36S | Tolerated | 0.11 | -1.901 | Neutral | Possibly damaging | 0.615 | Disease | 2 | 0.606 | Neutral | 7 | 0.164 |
| S612T | tolerated | 0.87 | 0.11 | Neutral | Possibly damaging | 0.596 | Neutral |  |  | Neutral |  |  |
| E665D | Intolerated | 0 | -0.686 | Neutral | Possibly damaging | 0.608 | Neutral |  |  | Neutral |  |  |
| S462N | tolerated | 0.26 | -1.352 | Neutral | Benign |  | Neutral |  |  | Neutral |  |  |
| F569I | tolerated | 0.39 | -0.616 | Neutral | Benign |  | Neutral |  |  | Neutral |  |  |
| A446T | tolerated | 1 | 1.16 | Neutral | Benign |  | Neutral |  |  | Neutral |  |  |
| R203Q | Intolerated | 0.04 | -1.95 | Neutral | Benign |  | Neutral |  |  | Neutral |  |  |
| E665K | Tolerated | 0.22 | -1.219 | Neutral | Benign |  | Neutral |  |  | Neutral |  |  |
| L242F | Intolerated | 0 | -3.159 | Deleterious | Probably damaging | 0.999 | Disease | 3 | 0.669 | Neutral | 2 | 0.422 |
| A126T | Intolerated | 0 | -3.269 | Deleterious | Probably damaging | 1 | Disease | 2 | 0.599 | Neutral | 8 | 0.109 |
| V213I | tolerated | 0.45 | 0.398 | Neutral | Benign |  | Neutral |  |  | Neutral |  |  |
| H648P | Tolerated | 0.19 | -0.751 | Neutral | Benign |  | Disease | 3 | 0.639 | Neutral | 7 | 0.136 |
| E546K | Intolerated | 0.02 | -2.391 | Neutral | Probably damaging | 0.966 | Disease | 7 | 0.843 | Neutral | 2 | 0.393 |
| E136K | Intolerated | 0 | -3.702 | Deleterious | Probably damaging | 1 | Disease | 0 | 0.52 | Neutral | 7 | 0.137 |
| H507P | Intolerated | 0 | -5.336 | Deleterious | Probably damaging | 1 | Neutral | 4 |  | Neutral | 1 |  |
| C559Y | Intolerated | 0 | -0.752 | Neutral | Benign | 0.149 | Neutral | 7 |  | Neutral | 0 |  |
| S592I | Intolerated | 0 | -3.017 | Deleterious | Possibly damaging | 0.8 | Neutral | 7 |  | Neutral | 7 |  |
| P282R | Tolerated | 0.68 | -3.671 | Deleterious | Benign | 0.038 | Neutral | 8 |  | Neutral | 8 |  |
| V426I | Intolerated | 0 | -0.209 | Neutral | Benign | 0.055 | Neutral | 8 |  | Neutral | 9 |  |
| Q305H | Intolerated | 0 | -2.037 | Neutral | Benign | 0.134 | Neutral | 7 |  | Neutral | 7 |  |
| L455M | Tolerated | 0.2 | -0.45 | Neutral | Probably damaging | 0.976 | Neutral | 6 |  | Neutral | 6 |  |
| E147G | Tolerated | 0.06 | -3.288 | Deleterious | Benign | 0.036 | Neutral | 6 |  | Neutral | 7 |  |
| K494R | Intolerated | 0 | -1.735 | Neutral | Possibly damaging | 0.890 | Neutral | 7 |  | Neutral | 8 |  |
| V519M | Intolerated | 0 | -0.485 | Neutral | Benign | 0.104 | Neutral | 9 |  | Neutral | 8 |  |
| M64L | Tolerated | 0.19 | -0.709 | Neutral | Benign | 0 | Neutral | 9 |  | Neutral | 9 |  |
| A70V | Tolerated | 0.25 | -0.797 | Neutral | Benign | 0.001 | Neutral | 1 |  | Neutral | 9 |  |
| K545R | Intolerated | 0 | -1.854 | Neutral | Benign | 0.058 | Neutral | 9 |  | Neutral | 7 |  |
| V406L | Tolerated | 0.88 | -1.047 | Neutral | Benign | 0.003 | Neutral | 8 |  | Neutral | 9 |  |
| L365M | Intolerated | 0 | -0.662 | Neutral | Probably damaging | 0.999 | Neutral | 8 |  | Neutral | 8 |  |
| L13P | Intolerated | 0.03 | -3.303 | Deleterious | Probably damaging | 1 | Neutral | 2 |  | Disease | 1 | 0.527 |
| V262A | Tolerated | 0.07 | -3.951 | Deleterious | Probably damaging | 0.999 | Neutral | 6 |  | Neutral | 9 |  |
| A625E | Intolerated | 0 | -1.309 | Neutral | Possibly damaging | 0.947 | Disease | 7 | 0.825 | Neutral | 2 |  |
| L520R | Intolerated | 0 | -1.507 | Neutral | Possibly damaging | 0.766 | Disease | 8 | 0.918 | Disease | 7 | 0.845 |
| F268L | Intolerated | 0 | -5.912 | Deleterious | Probably damaging | 1 | Neutral |  |  | Neutral |  |  |
| A265V | tolerated | 0.11 | 1.927 | Neutral | Benign | 0 | Neutral | 7 |  | Neutral | 9 |  |
