# Supplementary material for: Identification and in silico analysis of functional SNPs of human TAGAP protein: A comprehensive study
Source: PLoS One. 2018 Jan 12;13(1):e0188143. doi: 10.1371/journal.pone.0188143 (PMC5766082; doi:10.1371/journal.pone.0188143)
Supplement: S3 Table — (DOCX) [file pone.0188143.s004.docx]

**Effects of nsSNPs on structural & functional properties of *TAGAP* by MutPred server.**

| **Mutation** | **Probability of deleterious mutation** | **Top 5 features** |
| --- | --- | --- |
|  |  |  |
| T118M | 0.618 | Gain of sheet (P = 0.0827) |
|  |  | Loss of methylation at K114 (P = 0.1228) |
|  |  | Loss of helix (P = 0.1299) |
|  |  | Loss of disorder (P = 0.1404) |
|  |  | Gain of MoRF binding (P = 0.1441) |
| L100F | 0.573 | Loss of catalytic residue at L100 (P = 0.0139) |
|  |  | Loss of stability (P = 0.1672) |
|  |  | Gain of phosphorylation at T99 (P = 0.1797) |
|  |  | Loss of disorder (P = 0.1994) |
|  |  | Loss of helix (P = 0.3949) |
| F122L | 0.846 | Gain of disorder (P = 0.0569) |
|  |  | Loss of sheet (P = 0.0817) |
|  |  | Gain of MoRF binding (P = 0.1919) |
|  |  | Gain of loop (P = 0.2045) |
|  |  | Gain of phosphorylation at S117 (P = 0.3138) |
| G120E | 0.902 | Gain of disorder (P = 0.0547) |
|  |  | Loss of catalytic residue at P116 (P = 0.0762) |
|  |  | Loss of MoRF binding (P = 0.1167) |
|  |  | Gain of phosphorylation at S117 (P = 0.1311) |
|  |  | Gain of sheet (P = 0.1451) |
| N205S | 0.896 | Gain of phosphorylation at N205 (P = 0.0499) |
|  |  | Gain of methylation at K210 (P = 0.0937) |
|  |  | Gain of disorder (P = 0.0966) |
|  |  | Gain of ubiquitination at K210 (P = 0.1278) |
|  |  | Gain of glycosylation at P204 (P = 0.1353) |
| G141W | 0.663 | Loss of disorder (P = 0.0073) |
|  |  | Gain of solvent accessibility (P = 0.0421) |
|  |  | Loss of glycosylation at S140 (P = 0.0445) |
|  |  | Loss of phosphorylation at S140 (P = 0.1358) |
|  |  | Loss of stability (P = 0.1642) |
| V151M | 0.676 | Gain of disorder (P = 0.1376) |
|  |  | Gain of MoRF binding (P = 0.1725) |
|  |  | Loss of catalytic residue at E147 (P = 0.4164) |
|  |  | Loss of helix (P = 0.4763) |
|  |  | Gain of sheet (P = 0.6509) |
| A126T | 0.804 | Gain of phosphorylation at A126 (P = 0.0033) |
|  |  | Loss of ubiquitination at K129 (P = 0.1322) |
|  |  | Gain of disorder (P = 0.1481) |
|  |  | Gain of MoRF binding (P = 0.2351) |
|  |  | Loss of sheet (P = 0.302) |
| E136K | 0.498 | Gain of ubiquitination at E136 (P = 0.0238) |
|  |  | Gain of MoRF binding (P = 0.0269) |
|  |  | Loss of helix (P = 0.1706) |
|  |  | Gain of phosphorylation at S140 (P = 0.1932) |
|  |  | Loss of glycosylation at S140 (P = 0.219) |
