# Supplementary material for: Identification and in silico analysis of functional SNPs of human TAGAP protein: A comprehensive study
Source: PLoS One. 2018 Jan 12;13(1):e0188143. doi: 10.1371/journal.pone.0188143 (PMC5766082; doi:10.1371/journal.pone.0188143)
Supplement: S4 Table — (DOCX) [file pone.0188143.s005.docx]

| **Putative methylation sites predicted by PSSMe and BPB-PPMS** | | | | | |
| --- | --- | --- | --- | --- | --- |
| **PSSMe** | | | **BPB-PPMS** | | |
| **Position** | **Flanking residues** | **SVM Probability** | **Position** | **Flanking residues** | **Score** |
| 11 | KLRSSHNAS-K-TLNANNMET | 0.71965 | 168 | RSIPRKLLSSD | 0.533436 |
| 52 | DSICQLIEV-K-KRKKVLSWP | 0.58973 | 195* | RIEALKQVADK | 0.554706 |
| 82 | FSGALETDL-K-ASLFDQPLS | 0.88132 | 210 | NLLLLKHLVYV | 0.537371 |
| 114 | QDILTILCL-K-GPSTEGIFR | 0.54832 | 256* | SFEAQKDLNNK | 0.786528 |
| 129 | GIFRRAANE-K-ARKELKEEL | 0.89086 | 517* | TFAPHKKVLTK | 0.681053 |
| 132 | RRAANEKAR-K-ELKEELNSG | 0.88599 | 652 | RHRGSKEPLPG | 0.52379 |
| 135 | ANEKARKEL-K-EELNSGDAV | 0.82145 |  |  |  |
| 159 | PVHLLAVVF-K-DFLRSIPRK | 0.62921 |  |  |  |
| 195* | DEEDRIEAL-K-QVADKLPRP | 0.85082 |  |  |  |
| 200 | IEALKQVAD-K-LPRPNLLLL | 0.55768 |  |  |  |
| 256* | DQSLSFEAQ-K-DLNNKVKTL | 0.71764 |  |  |  |
| 261 | FEAQKDLNN-K-VKTLVEFLI | 0.77301 |  |  |  |
| 441 | GKTKRPVDL-K-IKNLAPGSV | 0.77142 |  |  |  |
| 458 | SVLPRALVL-K-AFSSSSLDA | 0.92755 |  |  |  |
| 494 | FSRHQSFTT-K-TEKGKPSRE | 0.75014 |  |  |  |
| 499 | SFTTKTEKG-K-PSREIKKHS | 0.64829 |  |  |  |
| 505 | EKGKPSREI-K-KHSMSFTFA | 0.91731 |  |  |  |
| 517* | SMSFTFAPH-K-KVLTKNLSA | 0.8835 |  |  |  |
| 522 | FAPHKKVLT-K-NLSAGSGKS | 0.77659 |  |  |  |
| 545 | RDHVPRGVR-K-ESQLAGRIV | 0.74167 |  |  |  |
| 705 | TVSESVQRN-K-RDCLVRRCS | 0.54317 |  |  |  |
| Where; *are methylation sites predicted by both PSSMe and BPB-PPMS | | | |  |  |

**Post translational modifications**

| **Putative phosphorylation sites predicted by NetPhos 3.1 and GPS 3.0** | | | | | | | |
| --- | --- | --- | --- | --- | --- | --- | --- |
|  | **NetPhos 3.1** | | | **GPS 3.0** | | | |
|  | **Position** | **Score** | **Kinase** | **Position** | **Score** | **Cutoff** | **Kinase** |
| **Serine (S)** | 6 | 0.994 | unsp | 5 | 1.692 | 1.597 | AGC/AKT/AKT1 |
|  | 26 | 0.898 | unsp | 59* | 1.635 | 1.597 | AGC/AKT/AKT1 |
|  | 41 | 0.556 | CKI | 230* | 2.048 | 1.597 | AGC/AKT/AKT1 |
|  | 44 | 0.552 | CKI | 323* | 1.837 | 1.597 | AGC/AKT/AKT1 |
|  | 59* | 0.87 | unsp | 354* | 2.846 | 1.597 | AGC/AKT/AKT1 |
|  | 68 | 0.996 | unsp | 367* | 1.942 | 1.597 | AGC/AKT/AKT1 |
|  | 71 | 0.908 | unsp | 377* | 3.808 | 1.597 | AGC/AKT/AKT1 |
|  | 74 | 0.506 | CKII | 411* | 2.462 | 1.597 | AGC/AKT/AKT1 |
|  | 84 | 0.994 | unsp | 415* | 2.298 | 1.597 | AGC/AKT/AKT1 |
|  | 91 | 0.92 | unsp |  |  |  |  |
|  | 117 | 0.843 | unsp |  |  |  |  |
|  | 140 | 0.57 | CKII |  |  |  |  |
|  | 164 | 0.705 | PKC |  |  |  |  |
|  | 172 | 0.624 | CKII |  |  |  |  |
|  | 223 | 0.534 | cdc2 |  |  |  |  |
|  | 230* | 0.649 | PKA |  |  |  |  |
|  | 249 | 0.546 | DNAPK |  |  |  |  |
|  | 251 | 0.941 | Unsp |  |  |  |  |
|  | 286 | 0.693 | Unsp |  |  |  |  |
|  | 289 | 0.608 | CKII |  |  |  |  |
|  | 292 | 0.997 | Unsp |  |  |  |  |
|  | 299 | 0.779 | Unsp |  |  |  |  |
|  | 302 | 0.953 | Unsp |  |  |  |  |
|  | 308 | 0.986 | Unsp |  |  |  |  |
|  | 312 | 0.639 | CKII |  |  |  |  |
|  | 319 | 0.52 | cdc2 |  |  |  |  |
|  | 321 | 0.818 | Unsp |  |  |  |  |
|  | 323* | 0.919 | Unsp |  |  |  |  |
|  | 327 | 0.994 | Unsp |  |  |  |  |
|  | 329 | 0.714 | Unsp |  |  |  |  |
|  | 354* | 0.996 | Unsp |  |  |  |  |
|  | 367* | 0.584 | RSK |  |  |  |  |
|  | 368 | 0.634 | PKA |  |  |  |  |
|  | 377* | 0.995 | Unsp |  |  |  |  |
|  | 380 | 0.853 | Unsp |  |  |  |  |
|  | 383 | 0.539 | cdc2 |  |  |  |  |
|  | 384 | 0.989 | unsp |  |  |  |  |
|  | 400 | 0.985 | Unsp |  |  |  |  |
|  | 411* | 0.974 | Unsp |  |  |  |  |
|  | 415* | 0.997 | Unsp |  |  |  |  |
|  | 461 | 0.831 | Unsp |  |  |  |  |
|  | 462 | 0.514 | cdc2 |  |  |  |  |
|  | 463 | 0.971 | Unsp |  |  |  |  |
|  | 464 | 0.672 | PKC |  |  |  |  |
|  | 468 | 0.93 | Unsp |  |  |  |  |
|  | 469 | 0.959 | Unsp |  |  |  |  |
|  | 471 | 0.616 | cdc2 |  |  |  |  |
|  | 472 | 0.994 | Unsp |  |  |  |  |
|  | 476 | 0.992 | unsp |  |  |  |  |
|  | 478 | 0.531 | PKC |  |  |  |  |
|  | 479 | 0.997 | Unsp |  |  |  |  |
|  | 486 | 0.502 | PKG |  |  |  |  |
|  | 490 | 0.58 | PKA |  |  |  |  |
|  | 501 | 0.955 | Unsp |  |  |  |  |
|  | 508 | 0.99 | Unsp |  |  |  |  |
|  | 510 | 0.949 | Unsp |  |  |  |  |
|  | 525 | 0.721 | Unsp |  |  |  |  |
|  | 528 | 0.991 | unsp |  |  |  |  |
|  | 531 | 0.944 | unsp |  |  |  |  |
|  | 547 | 0.987 | unsp |  |  |  |  |
|  | 577 | 0.992 | unsp |  |  |  |  |
|  | 592 | 0.97 | unsp |  |  |  |  |
|  | 595 | 0.997 | unsp |  |  |  |  |
|  | 610 | 0.848 | unsp |  |  |  |  |
|  | 612 | 0.63 | DNAPK |  |  |  |  |
|  | 617 | 0.553 | unsp |  |  |  |  |
|  | 622 | 0.85 | PKC |  |  |  |  |
|  | 646 | 0.728 | unsp |  |  |  |  |
|  | 651 | 0.997 | unsp |  |  |  |  |
|  | 661 | 0.519 | unsp |  |  |  |  |
|  | 670 | 0.613 | PKA |  |  |  |  |
|  | 679 | 0.523 | CKI |  |  |  |  |
|  | 698 | 0.977 | unsp |  |  |  |  |
|  | 700 | 0.987 | unsp |  |  |  |  |
|  | 714 | 0.888 | unsp |  |  |  |  |
|  | 729 | 0.585 | PKC |  |  |  |  |
| **Threonine (T)** | 12 | 0.67 | PKC | 264* | 2.394 | 1.597 | AGC/AKT/AKT1 |
|  | 20 | 0.505 | CKII |  |  |  |  |
|  | 79 | 0.676 | PKC |  |  |  |  |
|  | 99 | 0.561 | cdc2 |  |  |  |  |
|  | 264* | 0.869 | unsp |  |  |  |  |
|  | 288 | 0.561 | unsp |  |  |  |  |
|  | 296 | 0.561 | CKII |  |  |  |  |
|  | 361 | 0.823 | PKC |  |  |  |  |
|  | 396 | 0.765 | PKC |  |  |  |  |
|  | 398 | 0.635 | unsp |  |  |  |  |
|  | 434 | 0.914 | unsp |  |  |  |  |
|  | 492 | 0.918 | PKC |  |  |  |  |
|  | 493 | 0.742 | unsp |  |  |  |  |
|  | 495 | 0.886 | unsp |  |  |  |  |
|  | 512 | 0.825 | PKC |  |  |  |  |
|  | 565 | 0.663 | unsp |  |  |  |  |
|  | 614 | 0.719 | PKC |  |  |  |  |
|  | 672 | 0.86 | PKC |  |  |  |  |
| **Tyrosine (Y)** | 310 | 0.964 | unsp |  |  |  |  |
|  | 596 | 0.943 | unsp |  |  |  |  |
|  | 730 | 0.938 | unsp |  |  |  |  |
| Where; *are phosphorylation sites predicted by both NetPhos 3.1 and GPS 3.0 | | | | | | |  |

| **Putative uiquitylation sites predicted by Ub-Pred and BDM-PUB** | | | | | |  |  |  |  |  |  |  |  |  |
| --- | --- | --- | --- | --- | --- | --- | --- | --- | --- | --- | --- | --- | --- | --- |
| **BDM-PUB** | | | **Ub-Pred** | | |  |  |  |  |  |  |  |  |  |
| **Position** | **Score** | **Threshold** | **Position** | **Score** | **Ubiquitinated** |  |  |  |  |  |  |  |  |  |
| 2 | 3.27 | 0.3 | 11* | 0.82 | Yes   Medium confidence |  |  |  |  |  |  |  |  |  |
| 11* | 1.96 | 0.3 | 31 | 0.76 | Yes   Medium confidence |  |  |  |  |  |  |  |  |  |
| 52 | 0.75 | 0.3 | 82* | 0.75 | Yes   Medium confidence |  |  |  |  |  |  |  |  |  |
| 55 | 0.59 | 0.3 | 135* | 0.74 | Yes   Medium confidence |  |  |  |  |  |  |  |  |  |
| 56 | 0.9 | 0.3 | 256* | 0.63 | Yes   Low confidence |  |  |  |  |  |  |  |  |  |
| 82* | 0.61 | 0.3 | 366* | 0.72 | Yes   Medium confidence |  |  |  |  |  |  |  |  |  |
| 114 | 0.38 | 0.3 | 399 | 0.95 | Yes   High confidence |  |  |  |  |  |  |  |  |  |
| 129 | 1.64 | 0.3 | 458* | 0.73 | Yes   Medium confidence |  |  |  |  |  |  |  |  |  |
| 132 | 2.65 | 0.3 | 530* | 0.86 | Yes   High confidence |  |  |  |  |  |  |  |  |  |
| 135* | 1.49 | 0.3 | 652* | 0.93 | Yes   High confidence |  |  |  |  |  |  |  |  |  |
| 168 | 2.17 | 0.3 | 668* | 0.69 | Yes   Medium confidence |  |  |  |  |  |  |  |  |  |
| 195 | 1.41 | 0.3 | 727* | 0.65 | Yes   Low confidence |  |  |  |  |  |  |  |  |  |
| 200 | 2.94 | 0.3 |  |  |  |  |  |  |  |  |  |  |  |  |
| 210 | 1.69 | 0.3 |  |  |  |  |  |  |  |  |  |  |  |  |
| 256* | 1.25 | 0.3 |  |  |  |  |  |  |  |  |  |  |  |  |
| 366* | 2.43 | 0.3 |  |  |  |  |  |  |  |  |  |  |  |  |
| 433 | 0.69 | 0.3 |  |  |  |  |  |  |  |  |  |  |  |  |
| 435 | 1.88 | 0.3 |  |  |  |  |  |  |  |  |  |  |  |  |
| 441 | 0.61 | 0.3 |  |  |  |  |  |  |  |  |  |  |  |  |
| 443 | 1.62 | 0.3 |  |  |  |  |  |  |  |  |  |  |  |  |
| 458* | 2.23 | 0.3 |  |  |  |  |  |  |  |  |  |  |  |  |
| 481 | 2.49 | 0.3 |  |  |  |  |  |  |  |  |  |  |  |  |
| 497 | 2.04 | 0.3 |  |  |  |  |  |  |  |  |  |  |  |  |
| 499 | 1.11 | 0.3 |  |  |  |  |  |  |  |  |  |  |  |  |
| 505 | 1.73 | 0.3 |  |  |  |  |  |  |  |  |  |  |  |  |
| 517 | 2.02 | 0.3 |  |  |  |  |  |  |  |  |  |  |  |  |
| 518 | 1.94 | 0.3 |  |  |  |  |  |  |  |  |  |  |  |  |
| 522 | 2.31 | 0.3 |  |  |  |  |  |  |  |  |  |  |  |  |
| 530* | 2.13 | 0.3 |  |  |  |  |  |  |  |  |  |  |  |  |
| 545 | 0.8 | 0.3 |  |  |  |  |  |  |  |  |  |  |  |  |
| 652* | 2.27 | 0.3 |  |  |  |  |  |  |  |  |  |  |  |  |
| 668* | 1.67 | 0.3 |  |  |  |  |  |  |  |  |  |  |  |  |
| 727* | 0.97 | 0.3 |  |  |  |  |  |  |  |  |  |  |  |  |
| Where; Low confidence scores range from 0.62 ≤ s ≤ 0.69, Medium confidence  scores range from 0.69 ≤ s ≤ 0.84 and High Confidence scores range from 0.84 ≤ s ≤ 1.00. | | | | | | | | | | | | | | |
| *are ubiquitylation sites predicted by both Ub-Pred and BDM-PUB. | | | | | | |  |  |  |  |  |  |  |  |
